# Prognostic implications of alcohol dehydrogenases in hepatocellular carcinoma

**DOI:** 10.1186/s12885-020-07689-1

**Published:** 2020-12-07

**Authors:** Xiangye Liu, Tingting Li, Delong Kong, Hongjuan You, Fanyun Kong, Renxian Tang

**Affiliations:** 1 Jiangsu Key Laboratory of Immunity and Metabolism, Department of Pathogenic Biology and Immunology, Xuzhou Medical University, Xuzhou, Jiangsu Province 221004 P. R. China; 2 National Demonstration Center for Experimental Basic Medical Sciences Education, Xuzhou Medical University, Xuzhou, 221004 Jiangsu Province P. R. China

**Keywords:** Alcohol dehydrogenase, Hepatocellular carcinoma, Overall survival, Recurrence free survival, Expression level, Prognostic value

## Abstract

**Background:**

Hepatocellular carcinoma (HCC) is a malignancy with high incidence and mortality rates worldwide. Alcohol dehydrogenases (ADHs) are huge family of dehydrogenase enzymes and associated with the prognosis of various cancers. However, comprehensive analysis of prognostic implications related to ADHs in HCC is still lacking and largely unknown.

**Methods:**

The expression profiles and corresponding clinical information of HCC were obtained from The Cancer Genome Atlas (TCGA). Wilcoxon signed-rank test was employed to evaluate the expression of ADHs. Cox regression and Kaplan-Meier analyses were used to investigate the association between clinicopathological characteristics and survival. GO (Gene Ontology) and KEGG (Kyoto Encyclopedia of Genes and Genomes) enrichment analyses were performed and visualized using R/BiocManager package.

**Results:**

We found that the expression of ADH1A, ADH1B, ADH1C, ADH4, and ADH6 was significantly downregulated in HCC samples compared to normal liver samples. Our univariate and multivariate Cox regression analyses results showed that high expression of ADH1A, ADH1B, ADH1C, ADH4, and ADH6 was considered as an independent factor with an improved prognosis for the survival of HCC patients. Moreover, our Kaplan-Meier analysis results also revealed that high expression of AHD1A, ADH1B, ADH1C, ADH4, and ADH6 was significantly associated with good survival rate in HCC patients. In addition, GO, KEGG, and GSEA analyses unveiled several oncogenic signaling pathways were negatively associated high expression of ADHs in HCC.

**Conclusion:**

In the present study, our results provide the potential prognostic biomarkers or molecular targets for the patients with HCC.

## Background

Liver cancer is the sixth most common diagnosed cancer and the fourth most common cause of cancer-related deaths, which leads to about 841,080 (4.7%) new cases diagnosed and 781,631 (8.2%) deaths according to global cancer statistics 2018 [1]. Hepatocellular carcinoma (HCC) is the primary liver cancers, and accounts for approximately 75–85% of liver cancers worldwide [1]. Over the past few decades, considerable molecules have been developed to diagnose and treat HCC; where these molecules have been applied and shown their efficacy in detection and treatment of the disease [2, 3]. However, most of HCC patients are diagnosed at advanced stages resulting in the increasing mortality and poor prognosis in many parts of the world [4]. Therefore, identification of novel diagnostic biomarkers for early diagnosis of HCC is urgently needed.

Increasing evidence revealed that hepatitis B and C virus infection drove most of the global burden of HCC and accounted for 80% of HCC cases [5]. Moreover, it has been well documented the association between liver cirrhosis and the development of HCC [6]. In Europe and USA, alcoholic cirrhosis is considered as the second most important risk factor for HCC [7]. Therefore, alcohol is recognized as a primary cause or cofactor for the development of HCC in the patients who are heavy alcoholic [8, 9]. In liver, alcohol is primarily metabolized into acetaldehyde, which is also carcinogenic, through alcohol dehydrogenases [10]. Several studies have demonstrated that ADHs are the major rate-limiting factors in the process of alcohol metabolism [11].

Alcohol dehydrogenases (ADHs) are super family of dehydrogenase enzymes located on chromosome 4q22-q24, including class I (ADH1A, ADH1B, and ADH1C), class II (ADH4), class III (ADH5), class IV (ADH6), and class V (ADH7) [12]. ADH family members are widely expressed in several human tissues; interestingly, ADH7 is the only one which is not expressed in human liver [12, 13]. Previous studies have reported that members of ADH gene family are associated with various cancers [14, 15], and the genetic variation of ADHs also affects the risk of cancer for alcohol dependent individuals [16–18]. Recent investigations have shown the prognostic values of ADH family members for non-small cell lung cancer and gastric cancer [19, 20]. However, to the best of our knowledge, the prognostic value of ADH family members in HCC is still unclear. Therefore, the aim of our present study was to explore the potential prognostic value of ADH genes for HCC patients.

## Methods

### Data acquisition

The transcriptomic profiles and clinicopathological information of Liver Hepatocellular Carcinoma in TCGA (The Cancer Genome Atlas) were obtained from GDC (Genomic Data Commons Data Portal) website (https://portal.gdc.cancer.gov/). For transcriptomic data, FPKM (Fragments Per Kilobase of transcript per Million mapped reads) was used as a unit representing the gene expression levels. Tumor samples, which have full clinicopathological characteristics including age, gender, histologic grade, pathologic T stage, vital status, OS (overall survival), alcohol consumption status, recurrence status (new tumor event after initial treatment) and days to new tumor event after initial treatment, were selected for further analyses, resulting in 269 samples. RFS (recurrence free survival) was calculated depending on recurrence status and days to new tumor event after initial treatment. GSEA (Gene Set Enrichment Analysis) was performed with the JAVA program GSEA 4.0 and annotated gene set database including KEGG “c2.cp.kegg.v7.0.symbols.gmt” and PID “c2.cp.pid.v7.0.symbols.gmt” were chosen as the reference gene sets (minimal set size = 15, maximal set size = 500) [21]. The results were shown in the form of multiple-GSEA using R packages including plyr, ggplot2, grid, and gridExtra.

### Statistical analysis

Wilcoxon signed-rank test was employed to compare the statistical significance of ADHs’ expression in different samples. Cases were categorized into high and low expression subgroups based on Youden index calculation according to ADHs’ expression levels [22]. Survival analysis of cases was performed on Kaplan-Meier method with a log-rank test. The association between clinicopathological characteristics and survival was carried out by univariate Cox regression and multivariate Cox regression analyses with HR (Hazard Ratio) and 95% CI (Confidence Intervals). GO (Gene Ontology) and KEGG (Kyoto Encyclopedia of Genes and Genomes) enrichment analyses were performed and visualized using R/BiocManager package [23, 24]. Analyses were performed with R version 3.6.2 [25] and Graphpad Prism 5.0 (GraphPad Software, Inc., USA). All statistical analyses with a *p*-value < 0.05 were considered to be significant, and GSEA gene sets with *p*-value < 0.05 and a false discovery rate (FDR) < 0.25 were considered as a significantly enrichment.

## Results

### Gene expression signatures of ADHs across different HCC samples

**Fig. 1:**
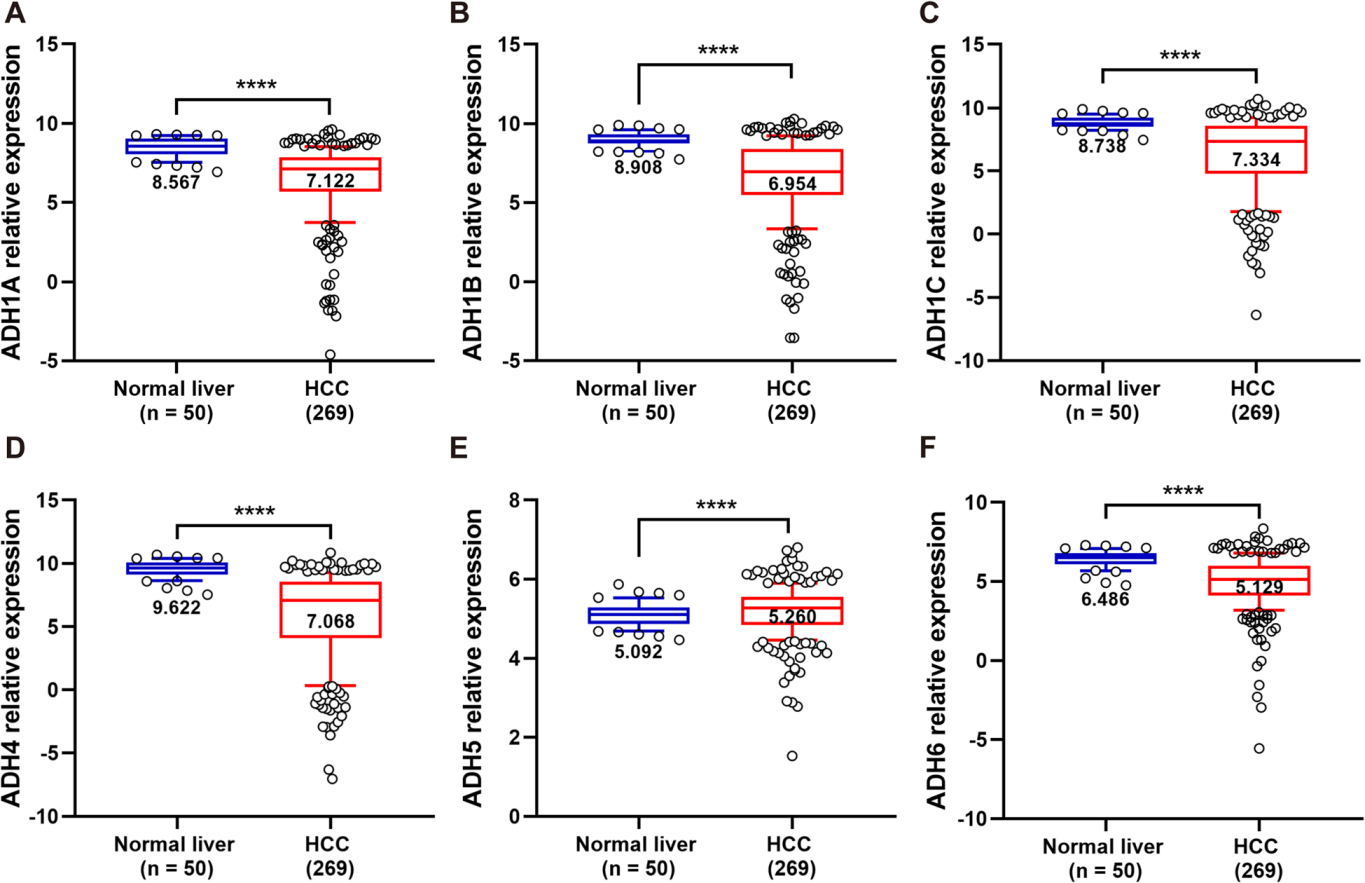
Gene expression level of ADHs in normal liver and HCC tissues. The gene expression level of ADH1A (**a**), AHD1B (**b**), ADH1C (**c**), ADH4 (**d**), ADH5 (**e**), and ADH6 (**f**) in normal liver (*n* = 50) and HCC (*n* = 269) tissue samples. The relative expression level of ADHs in each sample was shown as log2^FPKM^, all data were collected from TCGA. Data shown represent box and whisker plots (whiskers = 10–90 percentile). The significant difference was calculated with Wilcoxon signed-rank test for gene expression in normal liver and HCC tissue samples, *p* < 0.05 was recognized as significant difference, and the significant differences were denoted by **** for *p* < 0.0001

**Fig. 2:**
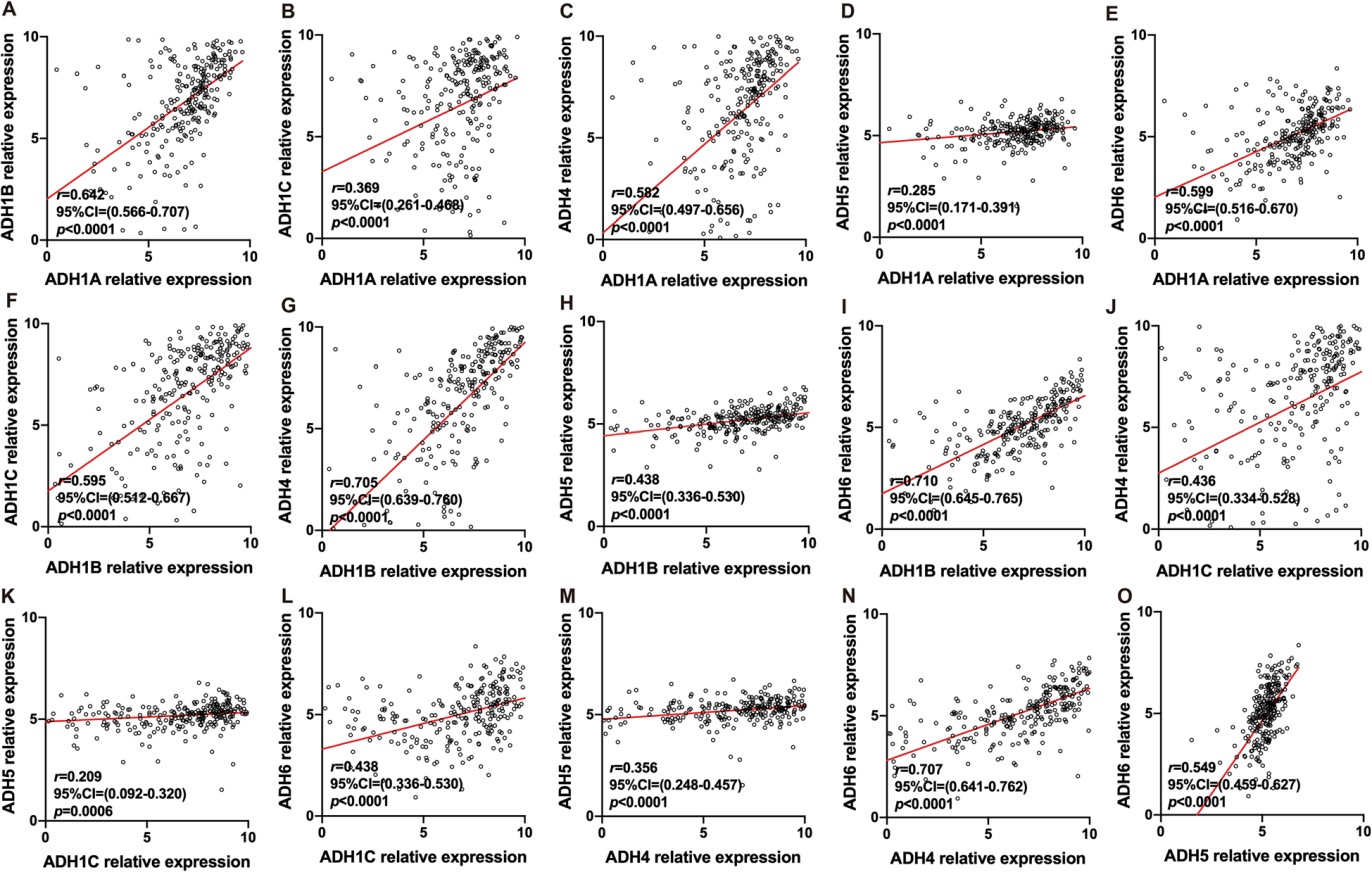
Co-expression of ADHs in HCC patients. Pearson correlation analysis was performed to characterize the co-expression of ADHs in HCC patients (*n* = 269). The relative expression level of ADHs in each sample was shown as log2^FPKM^, all data were collected from TCGA. *p* < 0.05 was recognized as significant difference

In order to distinguish the expression levels of ADHs between normal and tumor liver tissues, the transcriptome of 50 normal liver and 269 HCC samples was identified. As shown in Fig. 1, the expression level of ADH1A, ADH1B, ADH1C, ADH4, and ADH6 was significantly downregulated in HCC samples compared to normal liver samples. Interestingly, the expression level of ADH5 was slightly but significantly upregulated in HCC samples (Fig. 1e). Furthermore, all the ADH genes showed a positive correlation with each other (Fig. 2).

**Fig. 3:**
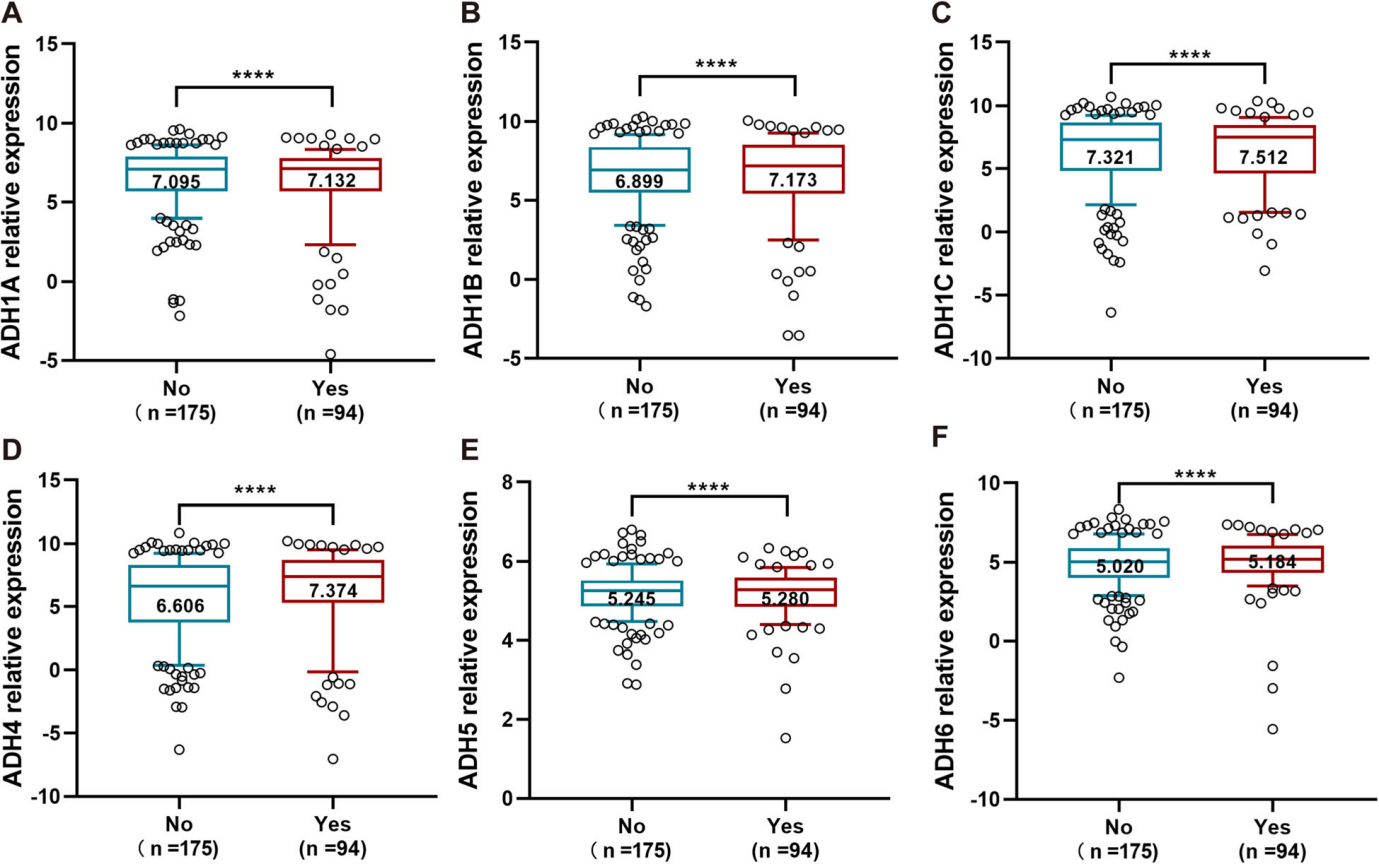
Gene expression level of ADHs in HCC patients with alcohol consumption and non-alcohol consumption. The gene expression level of ADH1A (**a**), AHD1B (**b**), ADH1C (**c**), ADH4 (**d**), ADH5 (**e**), and ADH6 (**f**) in non-alcohol consumption HCC patients (No, *n* = 175) and alcohol consumption HCC patients (Yes, *n* = 94). The relative expression level of ADHs in each sample was shown as log2^FPKM^, all data were collected from TCGA. Data shown represent box and whisker plots (whiskers = 10–90 percentile). The significant difference was calculated with Wilcoxon signed-rank test for gene expression in non- and alcohol consumption HCC patients, *p* < 0.05 was recognized as significant difference, and the significant differences were denoted by **** for *p* < 0.0001

Alcohol consumption increases the risk for liver cancer, and is also considered as a primary cause of HCC through the development of cirrhosis [26]. In the present study, our data showed that the expression level of ADH family members including ADH1A-ADH6 was significantly increased in alcohol consumption HCC patients compared to non-alcohol consumption ones (Fig. 3). Obviously, the expression of ADH1B and ADH4 was strongly upregulated (Fig. 3b and d).

**Fig. 4:**
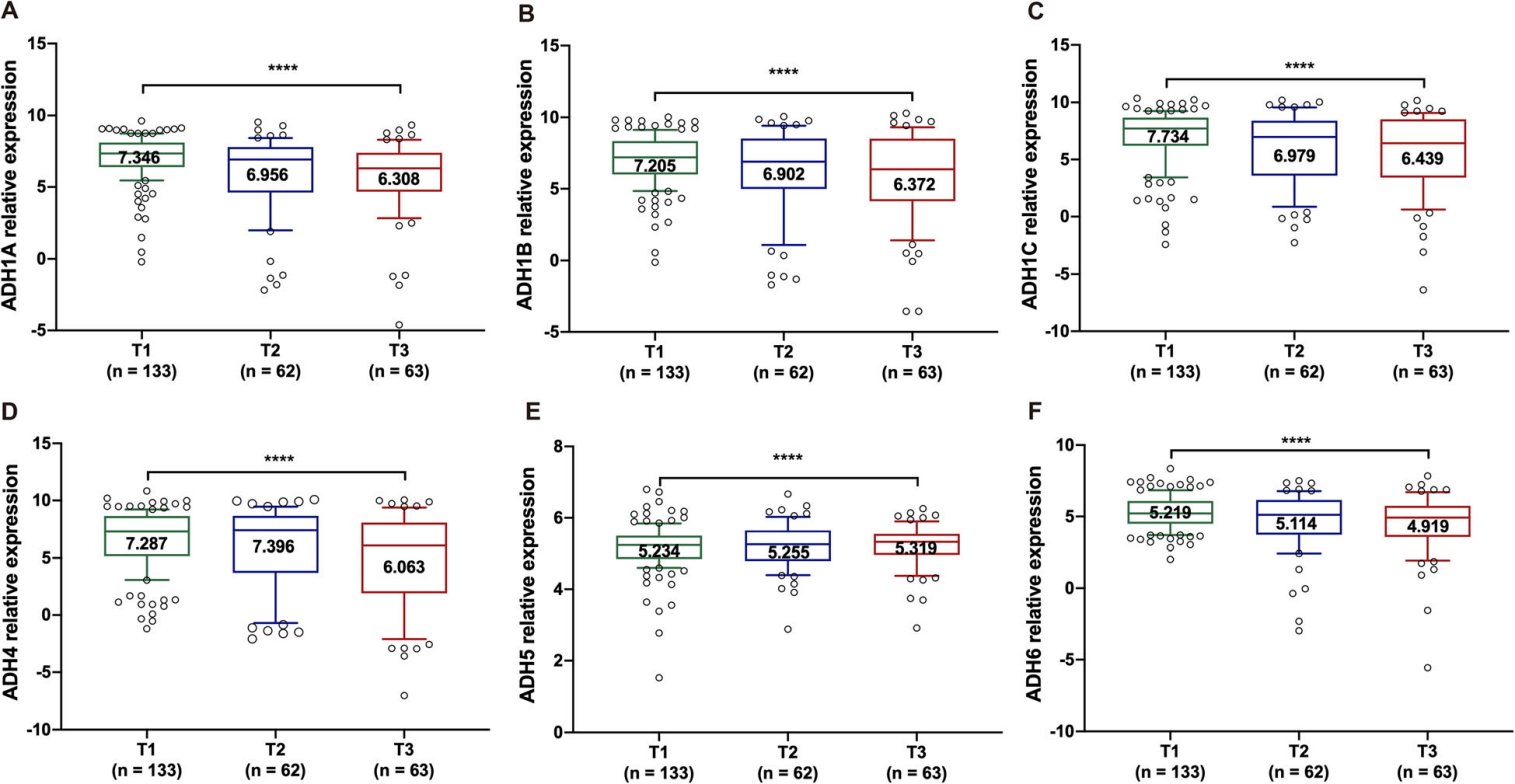
Gene expression level of ADHs in HCC patients with different pathologic T stage. The gene expression level of ADH1A (**a**), AHD1B (**b**), ADH1C (**c**), ADH4 (**d**), ADH5 (**e**), and ADH6 (**f**) in HCC patients with pathologic T1 (*n* = 133), T2 (*n* = 62) and T3 (*n* = 63) stage. The relative expression level of ADHs in each sample was shown as log2^FPKM^, all data were collected from TCGA. Data shown represent box and whisker plots (whiskers = 10–90 percentile). The significant difference was calculated with Wilcoxon signed-rank test for gene expression in different pathologic T stage HCC tissue samples, *p* < 0.05 was recognized as significant difference, and the significant differences were denoted by **** for *p* < 0.0001

In order to characterize the correlation between ADHs and tumor stages, the expression level of ADHs was identified in HCC samples with different pathologic T stages. Our results showed that the expression level of ADHs including ADH1A, ADH1B, ADH1C, and ADH6 was obviously decreased with the progression of tumor malignancy (Fig. 4a-c and f). And the expression level of ADH4 was higher in HCC patients with T2 pathologic stage than T1 and T3 pathologic stage, but it was lowest in HCC samples with T3 pathologic stage (Fig. 4d). While the expression level of ADH5 was remarkably increased with the progression of tumor malignancy (Fig. 4e).

### Association between ADHs expression and survival time of HCC patients

In order to explore the prognostic value of ADHs expression level in the patients with HCC, all the patients were categorized into high and low expression groups based on the expression level of ADHs as described above. Primarily, the association of ADHs expression and clinicopathological characteristics was determined with two-sided *Fisher’s* exact tests. Our results showed that the expression of ADH1B, ADH4, and ADH5 was significantly associated with age. Moreover, the expression of ADH1A, ADH1B, and ADH4 was significantly associated with histologic grade. In addition, ADH1A, ADH1B and ADH4 expressions were significantly associated with pathologic T stage (Table 1).

**Table 1:** Association analysis between ADHs expression and clinicopathological variables in 269 HCC patients

Variables	ADH1A			ADH1B			ADH1C			ADH4			ADH5			ADH6		
	High	Low	***p***	High	Low	***p***	High	Low	***p***	High	Low	***p***	High	Low	***p***	High	Low	***p***
**All patients**	112	157		174	95		87	182		186	83		114	155		143	126	
**Gender**			0.685			0.261			0.391			0.029			0.105			0.003
Males	78	113		128	63		65	126		140	51		87	104		113	78	
Females	34	44		46	32		22	56		46	32		27	51		30	48	
**Age (years)**			0.216			0.015			0.050			0.018			0.007			0.462
< 60	46	77		70	53		32	91		76	47		41	82		62	61	
≥ 60	66	80		104	42		55	91		110	36		73	73		81	65	
**Histologic grade**			0.005			0.0009			0.060			0.001			0.800			0.165
Low (Grade 1 + 2)	82	88		123	47		62	108		130	40		71	99		96	74	
High (Grade 3 + 4)	30	69		51	48		25	74		56	43		43	56		47	52	
**Pathologic T stage**			0.004			0.032			0.466			0.039			0.680			0.274
Low (T 1 + 2)	92	103		134	61		66	129		142	53		81	114		108	87	
High (T 3 + 4)	20	54		40	34		21	53		44	30		33	41		35	39	
**Alcohol consumption**			> 0.999			0.790			0.785			0.054			0.439			
Yes	39	55		62	32		29	65		72	22		43	51		52	42	0.611
No	73	102		112	63		58	117		114	61		71	104		91	84	
**Tumor recurrence**			0.269			0.899			0.118			0.088			0.805			0.903
Yes	50	81		84	47		36	95		84	47		57	74		69	62	
No	62	76		90	48		51	87		102	36		57	81		74	64	

**Fig. 5:**
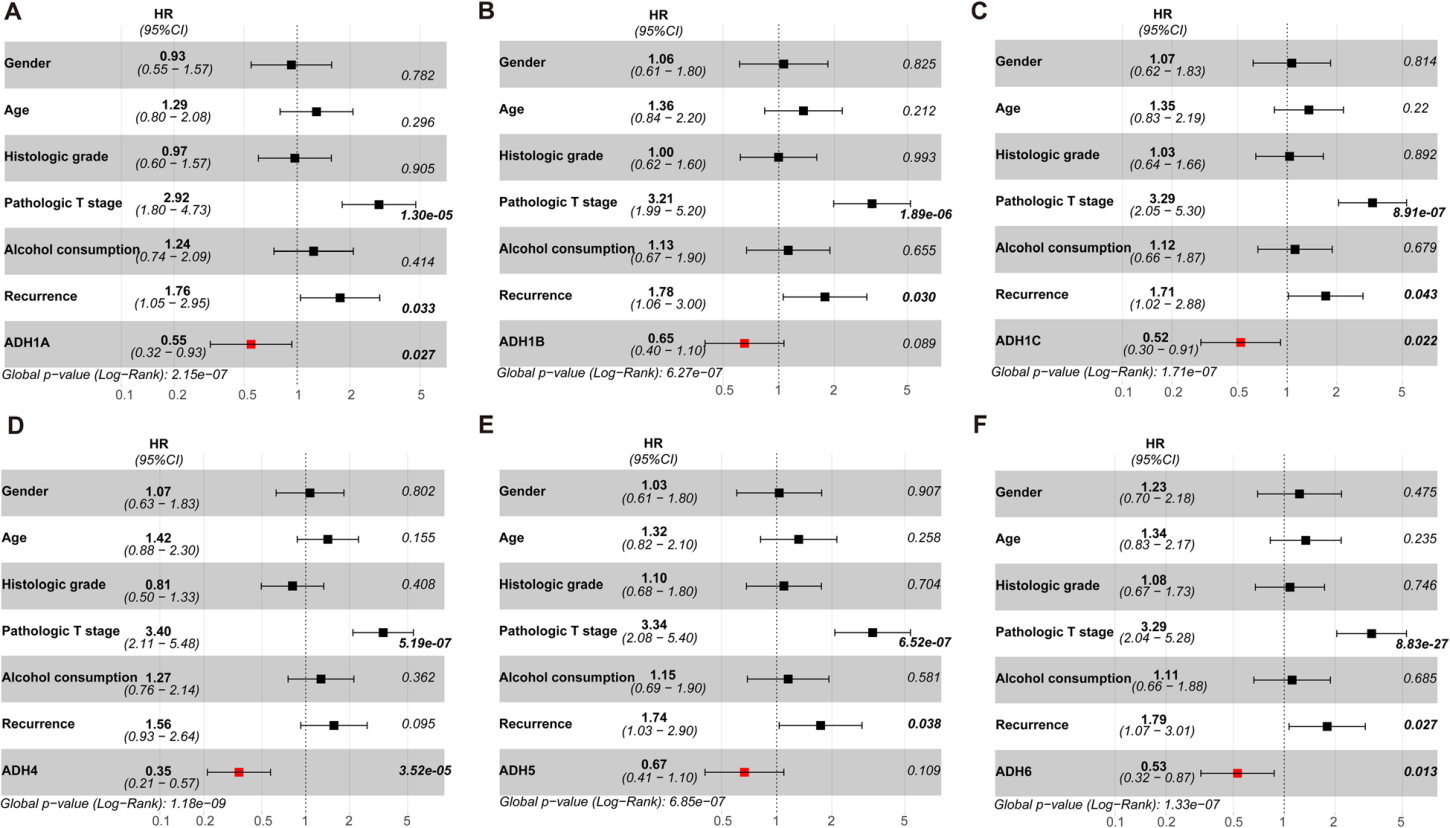
Multivariate Cox regression analysis for overall survival in HCC patients. Forest-plot showing the hazard ratio (HR) with 95% confidence index (CI) and *p* value for overall survival (OS) in patients with HCC (n = 269) based on high versus low expression of ADH family members, including ADH1A (**a**), AHD1B (**b**), ADH1C (**c**), ADH4 (**d**), ADH5 (**e**), and ADH6 (**f**). *p* < 0.05 was considered as significant difference

**Fig. 6:**
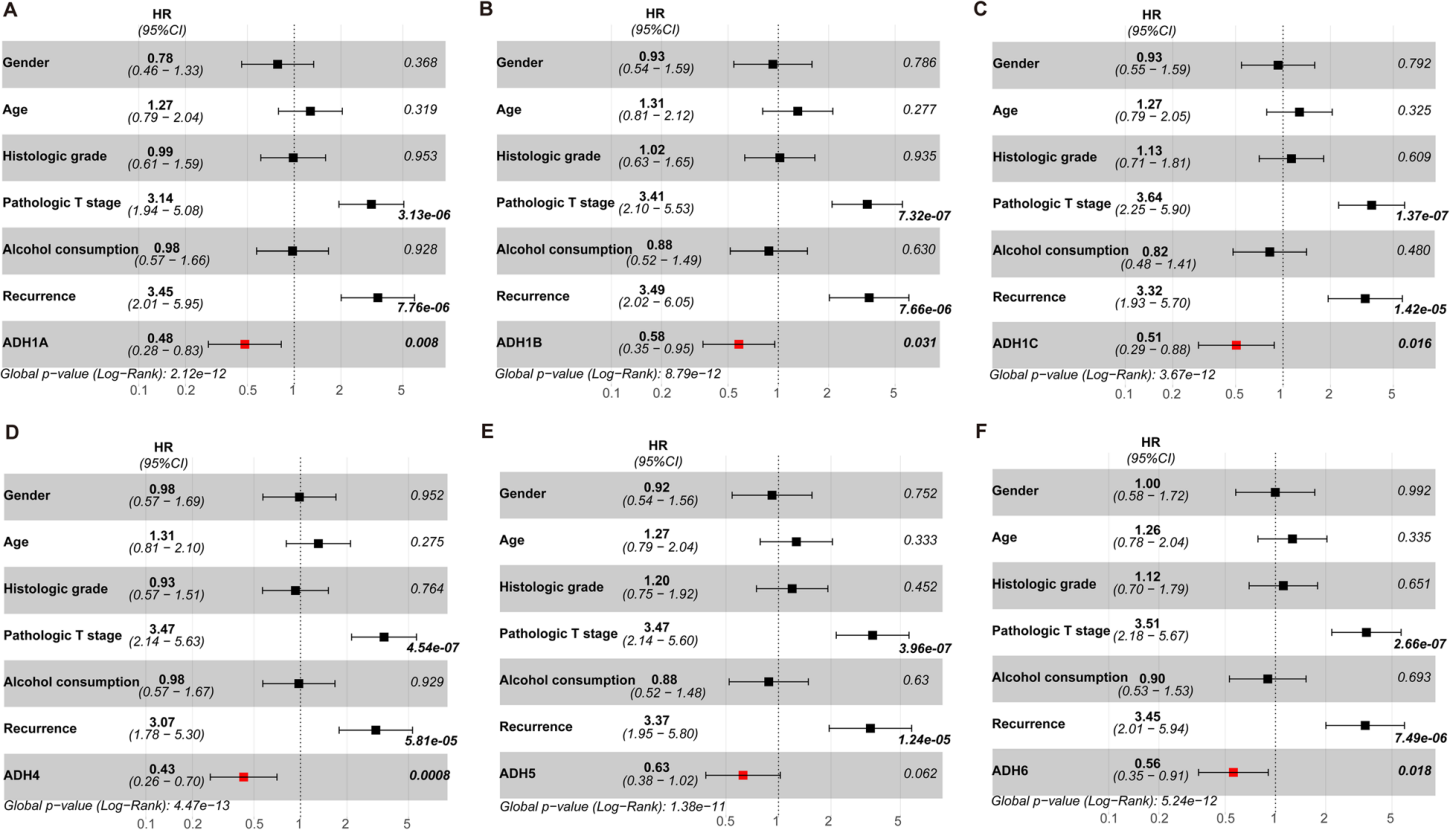
Multivariate Cox regression analysis for recurrence free survival in HCC patients. Forest-plot showing the HR with 95%CI and *p* value for recurrence free survival (RFS) in patients with HCC (n = 269) based on high versus low expression of ADH family members, including ADH1A (**a**), AHD1B (**b**), ADH1C (**c**), ADH4 (**d**), ADH5 (**e**), and ADH6 (**f**). *p* < 0.05 was considered as significant difference

Furthermore, univariate and multivariate Cox regression analyses were also performed to assess the prognostic value of ADHs expression and clinicopathological characteristics. As shown in Table 2, univariate Cox regression analysis results presented that high pathologic T stage, alcohol consumption, tumor recurrence were significantly associated with poor OS for HCC patients. Moreover, higher pathologic T stage and tumor recurrence were also significantly associated with poor RFS. Interestingly, high expression of ADH1A, ADH1C, ADH4, and ADH6 was considered as an independent factor with an improved prognosis for OS and RFS. While high expression of ADH1B was only considered as an independent factor with an improved prognosis for RFS (Table 2). In addition, our multivariate Cox regression analysis results showed that higher pathologic T stage and tumor recurrence were also significantly associated with poor OS and RFS (Figs. 5 and 6). Interestingly, high expression of ADH1A, ADH1C, ADH4, and ADH6 was also considered as an independent factor with an improved prognosis for OS (Fig. 5). And high expression of ADHs without ADH5 was considered as an independent factor with an improved prognosis for RFS (Fig. 6).

**Table 2:** Univariate analysis of the expression of ADHs expression with OS and RFS in 269 patients with HCC

Variables	OS		RFS	
	HR (95%CI)	***p*** value	HR (95%CI)	***p*** value
**Gender** (males vs. females)	0.904 (0.560–1.458)	0.678	0.798 (0.495–1.288)	0.356
**Age** (≤ 60 vs. > 60 years)	1.361 (0.856–2.162)	0.193	1.314 (0.827–2.089)	0.247
**Histologic grade** Low (Grade 1 + 2) vs. High (Grade 3 + 4)	1.051 (0.660–1.674)	0.833	1.144 (0.719–1.821)	0.571
**Pathologic T stage** Low (T1 + 2) vs. High (T3 + 4)	3.819 (2.419–6.030)	8.86e-09	4.386 (2.766–6.952)	3.21e-10
**Alcohol consumption** (yes vs. no)	1.279 (0.795–2.058)	3.10e-01	1.036 (0.646–1.662)	0.884
**Tumor recurrence** (yes vs. no)	2.279 (1.383–3.755)	0.001	4.201 (2.507–7.039)	5.06e-08
**ADH1A expression** (high vs. low)	0.436 (0.261–0.727)	0.001	0.415 (0.248–0.693)	0.0008
**ADH1B expression** (high vs. low)	0.641 (0.405–1.015)	0.058	0.609 (0.385–0.964)	0.034
**ADH1C expression** (high vs. low)	0.516 (0.300–0.888)	0.017	0.518 (0.301–0.892)	0.018
**ADH4 expression** (high vs. low)	0.380 (0.240–0.600)	3.41e-05	0.386 (0.245–0.609)	4.17e-05
**ADH5 expression** (high vs. low)	0.708 (0.436–1.148)	0.161	0.677 (0.418–1.096)	0.112
**ADH6 expression** (high vs. low)	0.544 (0.342–0.864)	0.009	0.558 (0.351–0.886)	0.013

**Fig. 7:**
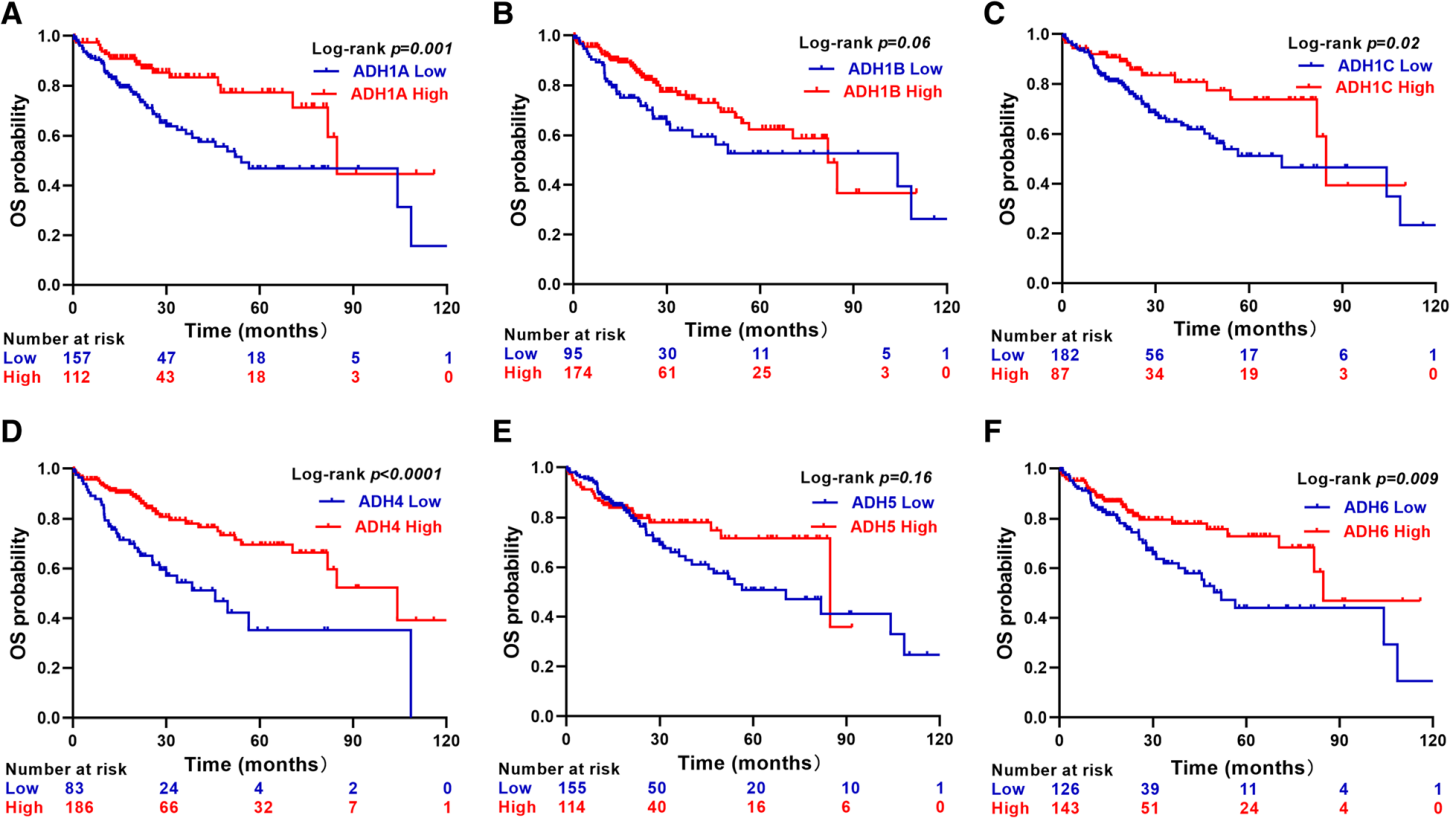
Overall survival analysis in HCC patients. Kaplan-Meier survival plots representing the probability of OS for HCC patients (n = 269) according to the expression level of ADH family members, including ADH1A (**a**), AHD1B (**b**), ADH1C (**c**), ADH4 (**d**), ADH5 (**e**), and ADH6 (**f**). *p* < 0.05 was considered as significant difference

**Fig. 8:**
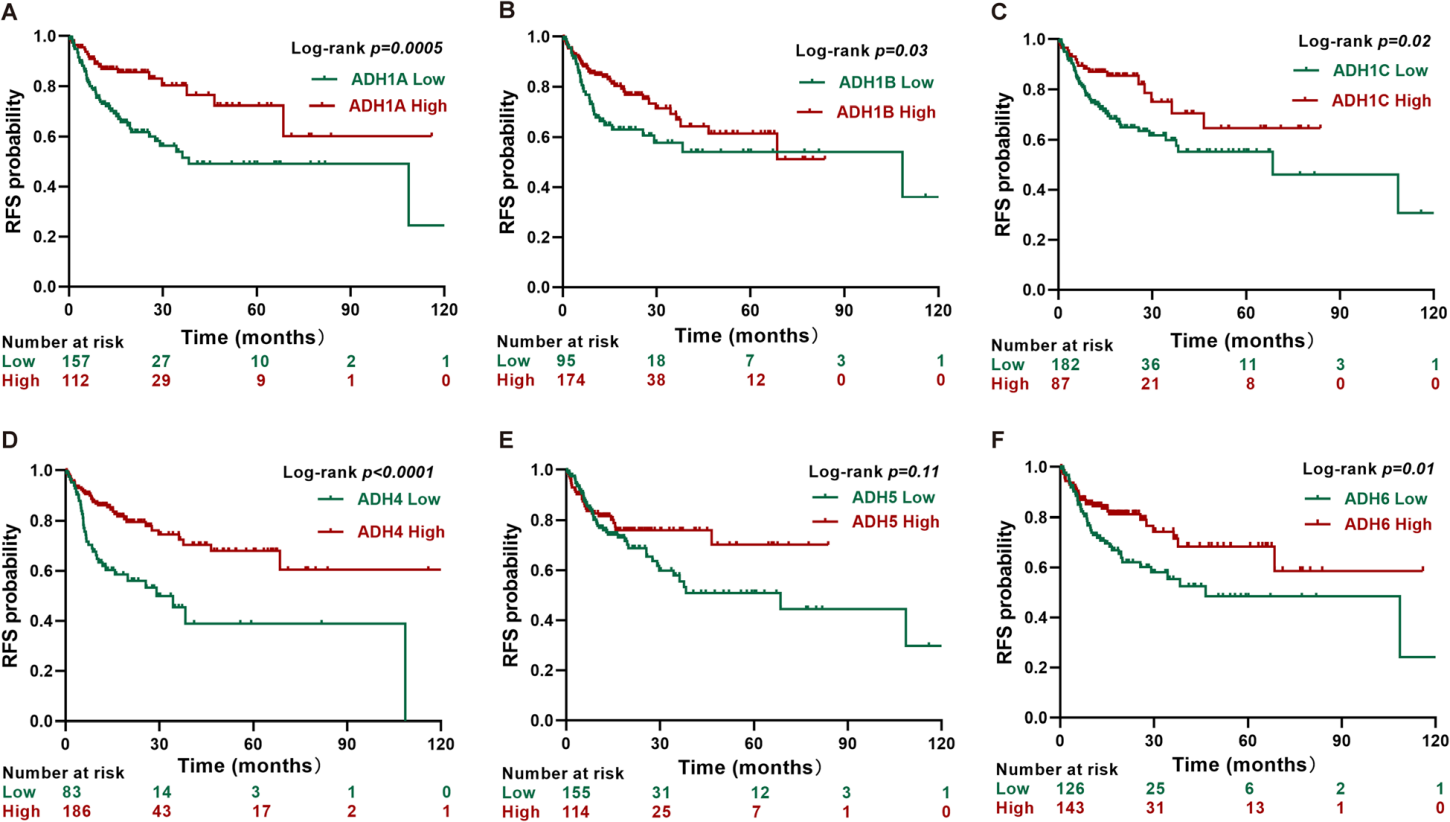
Recurrence free survival analysis in HCC patients. Kaplan-Meier survival plots representing the probability of RFS for HCC patients (n = 269) according to the expression level of ADH family members, including ADH1A (**a**), AHD1B (**b**), ADH1C (**c**), ADH4 (**d**), ADH5 (**e**), and ADH6 (**f**). *p* < 0.05 was considered as significant difference

Next, the correlation between ADHs expression and OS/RFS was evaluated with Kaplan-Meier analysis and log-rank test. Our results showed that high expression of AHD1A, ADH1C, ADH4, and ADH6 was significantly associated with good OS and RFS in HCC patients (Figs. 7 and 8). Interestingly, the RFS rate of HCC patients with high ADH1B expression was significantly better than that of patients with low ADH1B expression (Fig. 8b).

### Identification of involved pathways related to ADHs expression in HCC

**Fig. 9:**
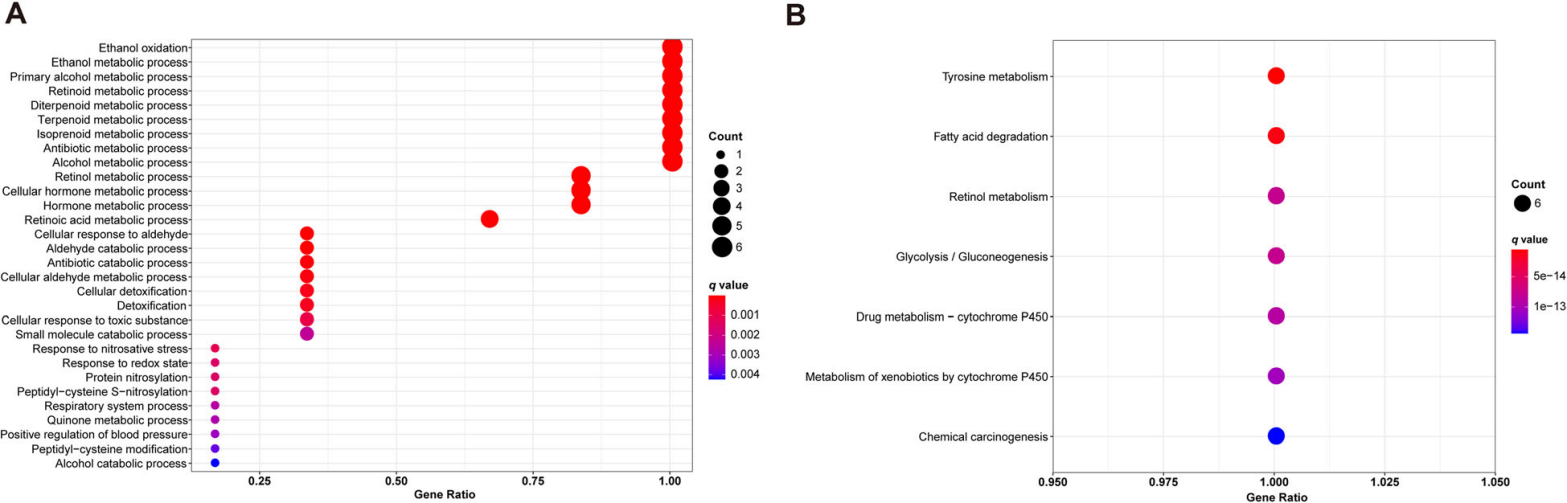
Functional enrichment analysis of ADH family members. **a** Dot-plot showing the significant enrichment terms (*p* < 0.05) in the Gene Ontology of ADH family members. **b** Dot-plot showing the significant enrichment pathways (*p* < 0.05) in the KEGG pathway of ADH family members

In order to identify the potential biological functions of ADHs, GO terms and KEGG pathways enrichment analysis was performed with R project as above description. GO terms enrichment analysis revealed that plenty of pathways were well enriched, and 9 of them including ethanol oxidation, ethanol metabolic process, primary alcohol metabolic process, retinoid metabolic process, diterpenoid metabolic process, terpenoid metabolic process, isoprenoid metabolic process, antibiotic metabolic process, and alcohol metabolic process were enriched for all the members of ADHs family (Fig. 9a and Table S1). Moreover, KEGG pathways enrichment analysis results showed that 7 pathways were significantly enriched, including tyrosine metabolism, fatty acid degradation, retinol metabolism, glycolysis/gluconeogenesis, drug metabolism-cytochrome P450, metabolism of xenobiotics by cytochrome P450, chemical carcinogenesis (Fig. 9b and Table S2).

**Fig. 10:**
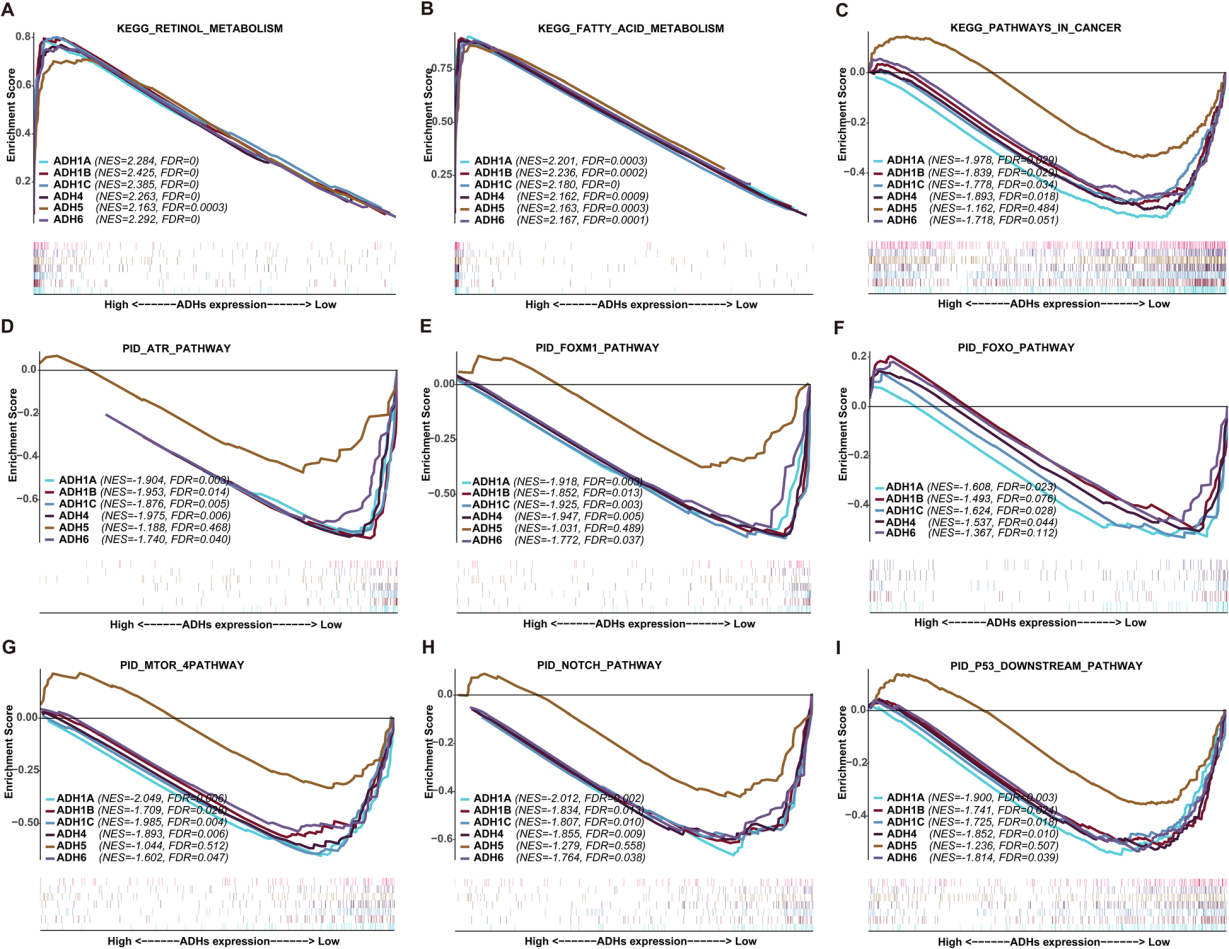
Gene set enrichment analysis of ADHs in HCC patients. Representative multi-GSEA plots showing ADHs high and low expression group in KEGG retinol metabolism (**a**), KEGG fatty acid metabolism (**b**), KEGG pathways in cancer (**c**), PID ATR pathway (**d**), PID FOXM1 pathway (**e**), PID FOXO pathway (**f**), PID MTOR 4pathway (**g**), PID NOTCH pathway (**h**), and PID P53 downstream pathway (**i**). GSEA gene sets with *p* < 0.05 and FDR < 0.25 were considered as a significant difference

In order to investigate the role of ADHs in the pathogenesis of HCC, GSEA was performed between datasets with ADHs high expression and low expression. Our results unveiled that ADHs were enriched in serval signaling pathways (Table S3 and S4). Based on the enrichment with KEGG database, the high expression group of ADHs was positively associated with retinol metabolism, and fatty acid metabolism with the significant difference (Fig. 10a and b). And the low expression group of ADHs family members not including ADH5 was significantly but negatively associated with pathways in cancer (Fig. 10c). Furthermore, we utilized GSEA to enrich in cancer related pathways based on pathway interaction database (PID). As can be seen from Fig. 10d-i, low expression group of ADHs family members without ADH5 was significantly enriched in several cancer related pathways, including ATR, FOXM1, FOXO, MTOR, NOTCH, and P53 downstream pathway.

## Discussion

HCC is a malignant tumor with high incidence and mortality rates worldwide [27]. The occurrence and progression of HCC are complex process, which is modulated through various numbers of oncogenes and anti-tumor genes. ADHs are huge family covering 7 members that are mainly involved in the conversion between alcohol and acetaldehyde, and also correlated to several hepatic diseases [14, 28]. However, the prognostic value of ADHs in the patients with HCC is still unclear. In the present study, we performed comprehensive analysis to investigate ADH genes association with the progression and prognosis of the patients with HCC, and to explore a series of diagnostic biomarkers of HCC.

The ADH family members are widely expressed in human liver without ADH7 [12, 13]. Therefore, ADH1A-ADH6 were selected to evaluate the prognostic value of ADHs in the patients with HCC. Primarily, our results revealed that the expression levels of ADH1A-ADH4, and ADH6 were significantly decreased in HCC tissues compared to normal liver tissues (Fig. 1), which were similar to their expression in non-small cell lung cancer (NSCLC) and pancreatic adenocarcinoma [19, 29]. Therefore, we speculate that ADH1A-ADH4, and ADH6 may serve as tumor suppressors in HCC. Whereas, the expression of ADH5 were significantly upregulated in HCC tissues compared to normal liver tissues (Fig. 1e), consistent with what is already known in NSCLC, gastric cancer, and pancreatic adenocarcinoma [19, 20, 29]. In addition, previous studies have reported the positive correlation of ADHs with each other in numerous cancers [19, 20]; here, our results also confirmed that in HCC (Fig. 2).

Smoking is an important risk factor for lung cancer, a recent study reported that the expression levels of ADHs without ADH1A were significantly associated with smoking status of the NSCLC patients [19]. Interestingly, alcohol consumption status is considered as a primary cause for HCC; here, our results revealed that the expression levels of ADH1A-ADH6 were obviously increased in alcohol consumption HCC patients (Fig. 3). Moreover, accumulating evidences have demonstrated that ADH family members were correlated with clinical stages, pathological grades, and TNM classifications in several cancers [19, 29, 30]. In our present study, it showed that the expression levels of ADH1A-ADH1C, and ADH6 were remarkably downregulated according to the pathologic T stage progression of HCC (Fig. 4). In addition, the expression of ADH1A, ADH1B and ADH4 was significantly associated with pathologic T stage (Table 1). Altogether, ADH family members play an important role in the development process of HCC. It is meaningful to evaluate the prognostic value for HCC patients.

In recent years, large numbers of investigations have reported that gene polymorphism of ADHs was correlated with cancer risk [15, 17, 18, 31, 32] . Moreover, the activity of ADH isoenzymes was significantly higher in liver cancer tissues than in healthy tissues [33], suggested the diagnostic value of ADH for the patients with liver cancer. However, rare studies have been performed to evaluate the prognostic value of ADHs mRNA expression in HCC. In the present study, our univariate and multivariate Cox regression analyses results indicated that high ADH1A, ADH1C, ADH4, and ADH6 levels independently predicted improved OS and RFS in HCC patients; whereas, high ADH1B levels independently predicted improved RFS in HCC patients (Table 2, Figs. 5 and 6). Furthermore, our Kaplan-Meier analysis data also revealed that high ADH1A, ADH1C, ADH4, and ADH6 levels predicted good OS and RFS in HCC patients; while, high ADH1B only predicted good RFS in HCC patients (Figs. 7 and 8). Recently, the expression of ADH1A was measured by using MS/MS and TMA in CHCC-HBV patients, which indicated the robust prognostic value of ADH1A for potential clinical application [34]. Moreover, Chen Q, et al also reported that high expression of ADH1C was associated with a good prognosis for HCC patients by using the TCGA internal and three GEO (GSE76427, GSE15654, and GSE14520) external validation cohorts [35]. In addition, the prognostic value of ADH4 was also confirmed by immunohistochemical analysis with 91 paraffin-embedded HCC specimens [36]. Although, a recent study reported that decreased ADH5 expression in HBV-related HCC tumor tissue predicted earlier recurrence [30]. Surprisingly, ADH5 expression could not play a significant role in prediction of OS and RFS in HCC patients depending on our present data. Therefore, our data supposed that ADH family members without ADH5 might serve as the potential biomarkers for the patients with HCC.

ADHs play a pivotal role in the metabolic process of ethanol [26]. Our GO enrichment analysis showed that ADHs were contributed to ethanol metabolism, such as ethanol oxidation, ethanol metabolic process, and primary alcohol metabolic process. Moreover, KEGG enrichment analysis indicated that ADHs were involved in fatty acid degradation, retinol metabolism, and so on (Fig. 9), constitute with what is investigated using GSEA (Fig. 10a and b). In addition, our GSEA results also showed that high expression group of ADHs was significantly and negatively associated with pathways in cancer without ADH5 (Fig. 10c), which suggested that high expression of ADHs could inhibit cancer related pathways and ADHs presented the tumor suppressor role. Increasing evidences indicated that various numbers of signaling pathways participated in the progression of HCC [37]. In order to determine the signaling pathways related to ADHs in HCC patients, GSEA was performed based on PID dataset [38]. Our results showed that low expression group of ADHs without ADH5 was positively related to various pathways (Fig. 10, Table S3 and S4). Interestingly, most of the signaling pathways contributed to promote tumorigenesis, such as ATR pathway [39], FOXM1 and FOXO pathways [40, 41], MTOR pathway [42], NOTCH pathway [43], and P53 downstream pathway [44]. Accordingly, high expression of ADHs could inhibit the malignant process of HCC, which promote the OS and RFS probability of HCC patients. Although low expression group of ADH5 was positively related to oncogenic signaling pathways, such as ATR, FOXM1, MTOR, NOTCH, and P53 downstream pathways, the statistic difference was not significant. Interestingly, low expression group of ADH5 was not positively related to FOXO signaling pathway, which may be associated with the high expression of ADH5 in HCC. By summarizing all the points, ADHs without ADH5 might act as the tumor suppressor via inhibiting oncogenic signaling pathway in HCC.

There were some limitations in our present study, which should be known. Primarily, the clinicopathological information of HCC patients from TCGA website, such as tumor size, hepatitis virus infection, α-fetoprotein, non-alcoholic fatty liver and cirrhosis, was not comprehensive or lost. Secondly, our present study investigates the prognostic value of ADHs for HCC patients at mRNA levels, which is not consummate. Therefore, their prognostic value for HCC patients at protein levels should be further evaluated in our future studies. Thirdly, ADH1A, ADH1B, ADH1C, ADH4, and ADH6 were all associated with the prognosis of HCC patients. In the future, the in vitro and in vivo experiments should be performed to investigate their roles in the progress of HCC, then the best one of them could be selected as diagnostic and prognostic biomarkers for HCC patients. In addition, further effort is also needed to elucidate the mechanisms, which ADHs contribute to the progress of HCC in the future.

## Conclusions

In the present study, our results were to explore the expression levels of ADHs in different grouping HCC patients, and also evaluated the correlation between prognosis of HCC and the expression patterns of ADHs. We found that the expression of ADH1A, ADH1B, ADH1C, ADH4, and ADH6 was significantly downregulated in HCC samples compared to normal liver samples; but upregulated in alcohol consumption HCC patients. Moreover, high expression of ADH1A, ADH1B, ADH1C, ADH4, and ADH6 was associated with good prognosis for HCC patients. Therefore, our results provide the potential prognostic biomarkers or molecular targets for the patients with HCC.

## Supplementary Information

**Additional file 1: Table S1.** Enrichment analysis of GO terms for ADHs.

**Additional file 2: Table S2.** Enrichment analysis of KEGG pathways for ADHs.

**Additional file 3: Table S3.** Signaling pathways enriched in HCC samples corresponding ADHs expression by GSEA based on KEGG.

**Additional file 4: Table S4.** Signaling pathways enriched in HCC samples corresponding ADHs expression by GSEA based on PID.

## Data Availability

The datasets analyzed in the current study are available in the TCGA repository (https://portal.gdc.cancer.gov/repository).

## Abbreviations

ADHs: Alcohol dehydrogenases
ATR: Ataxia telangiectasia and rad3-related
CHCC-HBV: Chinese HCC patients with HBV infection
CI: Confidence interval
FOX: Forkhead box
GO: Gene ontology
GSEA: Gene set enrichment analysis
HCC: Hepatocellular carcinoma
HR: Hazard ratio
KEGG: Kyoto encyclopedia of genes and genomes
MS: Mass spectrometry
MTOR: Mammalian target of rapamycin
NSCLC: Non-small cell lung cancer
OS: Overall survival
PID: Pathway interaction database
RFS: Recurrence-free survival
TCGA: The Cancer Genome Atlas
TMA: Tissue microarray
