# Supplementary material for: Prognostic implications of alcohol dehydrogenases in hepatocellular carcinoma
Source: BMC Cancer. 2020 Dec 7;20:1204. doi: 10.1186/s12885-020-07689-1 (PMC7720489; doi:10.1186/s12885-020-07689-1)
Supplement: Supplementary file 1 — Additional file 1: Table S1. Enrichment analysis of GO terms for ADHs. [file 12885_2020_7689_MOESM1_ESM.docx]

**Table S1. Enrichment analysis of GO terms for ADHs**

| **GO ID** | **Description** | ***p*-value** | ***q*-value** | **Gene ID** | **Count** |
| --- | --- | --- | --- | --- | --- |
| GO:0006069 | ethanol oxidation | 1.57E-20 | 1.16E-19 | ADH1A/ADH1B/ADH1C/ADH4/ADH5/ADH6 | 6 |
| GO:0006067 | ethanol metabolic process | 9.23E-19 | 3.40E-18 | ADH1A/ADH1B/ADH1C/ADH4/ADH5/ADH6 | 6 |
| GO:0034308 | primary alcohol metabolic process | 7.44E-15 | 1.83E-14 | ADH1A/ADH1B/ADH1C/ADH4/ADH5/ADH6 | 6 |
| GO:0001523 | retinoid metabolic process | 2.58E-14 | 4.76E-14 | ADH1A/ADH1B/ADH1C/ADH4/ADH5/ADH6 | 6 |
| GO:0016101 | diterpenoid metabolic process | 3.64E-14 | 5.37E-14 | ADH1A/ADH1B/ADH1C/ADH4/ADH5/ADH6 | 6 |
| GO:0006721 | terpenoid metabolic process | 6.21E-14 | 7.63E-14 | ADH1A/ADH1B/ADH1C/ADH4/ADH5/ADH6 | 6 |
| GO:0006720 | isoprenoid metabolic process | 1.53E-13 | 1.61E-13 | ADH1A/ADH1B/ADH1C/ADH4/ADH5/ADH6 | 6 |
| GO:0042572 | retinol metabolic process | 2.38E-13 | 2.07E-13 | ADH1A/ADH1B/ADH1C/ADH4/ADH6 | 5 |
| GO:0016999 | antibiotic metabolic process | 2.53E-13 | 2.07E-13 | ADH1A/ADH1B/ADH1C/ADH4/ADH5/ADH6 | 6 |
| GO:0006066 | alcohol metabolic process | 5.27E-11 | 3.89E-11 | ADH1A/ADH1B/ADH1C/ADH4/ADH5/ADH6 | 6 |
| GO:0034754 | cellular hormone metabolic process | 8.69E-11 | 5.82E-11 | ADH1A/ADH1B/ADH1C/ADH4/ADH6 | 5 |
| GO:0042573 | retinoic acid metabolic process | 1.06E-10 | 6.53E-11 | ADH1A/ADH1B/ADH1C/ADH6 | 4 |
| GO:0042445 | hormone metabolic process | 1.69E-09 | 9.56E-10 | ADH1A/ADH1B/ADH1C/ADH4/ADH6 | 5 |
| GO:0110096 | cellular response to aldehyde | 3.87E-06 | 2.04E-06 | ADH4/ADH5 | 2 |
| GO:0046185 | aldehyde catabolic process | 4.73E-06 | 2.32E-06 | ADH4/ADH5 | 2 |
| GO:0017001 | antibiotic catabolic process | 0.000141 | 6.50E-05 | ADH4/ADH5 | 2 |
| GO:0006081 | cellular aldehyde metabolic process | 0.00023 | 9.97E-05 | ADH4/ADH5 | 2 |
| GO:1990748 | cellular detoxification | 0.000527 | 0.000216 | ADH4/ADH5 | 2 |
| GO:0098754 | detoxification | 0.000719 | 0.000279 | ADH4/ADH5 | 2 |
| GO:0097237 | cellular response to toxic substance | 0.002525 | 0.00093 | ADH4/ADH5 | 2 |
| GO:0051409 | response to nitrosative stress | 0.00321 | 0.001126 | ADH5 | 1 |
| GO:0051775 | response to redox state | 0.004491 | 0.001477 | ADH5 | 1 |
| GO:0017014 | protein nitrosylation | 0.004812 | 0.001477 | ADH5 | 1 |
| GO:0018119 | peptidyl-cysteine S-nitrosylation | 0.004812 | 0.001477 | ADH5 | 1 |
| GO:0044282 | small molecule catabolic process | 0.007979 | 0.002352 | ADH4/ADH5 | 2 |
| GO:0003016 | respiratory system process | 0.009285 | 0.002631 | ADH5 | 1 |
| GO:1901661 | quinone metabolic process | 0.010241 | 0.002795 | ADH4 | 1 |
| GO:0045777 | positive regulation of blood pressure | 0.011834 | 0.003114 | ADH5 | 1 |
| GO:0018198 | peptidyl-cysteine modification | 0.015012 | 0.003814 | ADH5 | 1 |
| GO:0046164 | alcohol catabolic process | 0.017231 | 0.004232 | ADH4 | 1 |
| GO:0007585 | respiratory gaseous exchange | 0.019763 | 0.004697 | ADH5 | 1 |
| GO:1901616 | organic hydroxy compound catabolic process | 0.02355 | 0.005423 | ADH4 | 1 |
| GO:0045471 | response to ethanol | 0.03951 | 0.008822 | ADH6 | 1 |
| GO:0004745 | retinol dehydrogenase activity | 6.43E-15 | 2.03E-14 | ADH1A/ADH1B/ADH1C/ADH4/ADH6 | 5 |
| GO:0016616 | oxidoreductase activity, acting on the CH-OH group of donors, NAD or NADP as acceptor | 8.14E-14 | 1.29E-13 | ADH1A/ADH1B/ADH1C/ADH4/ADH5/ADH6 | 6 |
| GO:0016614 | oxidoreductase activity, acting on CH-OH group of donors | 1.27E-13 | 1.34E-13 | ADH1A/ADH1B/ADH1C/ADH4/ADH5/ADH6 | 6 |
| GO:0016620 | oxidoreductase activity, acting on the aldehyde or oxo group of donors, NAD or NADP as acceptor | 5.67E-05 | 4.48E-05 | ADH4/ADH5 | 2 |
| GO:0016903 | oxidoreductase activity, acting on the aldehyde or oxo group of donors | 8.60E-05 | 5.43E-05 | ADH4/ADH5 | 2 |
| GO:0004032 | alditol:NADP+ 1-oxidoreductase activity | 0.0044 | 0.001899 | ADH4 | 1 |
| GO:0004029 | aldehyde dehydrogenase (NAD) activity | 0.005076 | 0.001899 | ADH5 | 1 |
| GO:0019841 | retinol binding | 0.005076 | 0.001899 | ADH4 | 1 |
| GO:0016918 | retinal binding | 0.005413 | 0.001899 | ADH4 | 1 |
| GO:0008106 | alcohol dehydrogenase (NADP+) activity | 0.0071 | 0.002242 | ADH4 | 1 |
| GO:0004033 | aldo-keto reductase (NADP) activity | 0.008784 | 0.002522 | ADH4 | 1 |
| GO:0005504 | fatty acid binding | 0.011474 | 0.00274 | ADH5 | 1 |
| GO:0005501 | retinoid binding | 0.01181 | 0.00274 | ADH4 | 1 |
| GO:0019840 | isoprenoid binding | 0.012145 | 0.00274 | ADH4 | 1 |
| GO:0051287 | NAD binding | 0.018839 | 0.003966 | ADH4 | 1 |
| GO:0016655 | oxidoreductase activity, acting on NAD(P)H, quinone or similar compound as acceptor | 0.020174 | 0.003982 | ADH4 | 1 |
| GO:0033293 | monocarboxylic acid binding | 0.021506 | 0.003995 | ADH5 | 1 |
| GO:0043178 | alcohol binding | 0.028479 | 0.004996 | ADH4 | 1 |
| GO:0016651 | oxidoreductase activity, acting on NAD(P)H | 0.035738 | 0.00594 | ADH4 | 1 |
| GO:0009055 | electron transfer activity | 0.038039 | 0.006006 | ADH5 | 1 |
| GO:0019842 | vitamin binding | 0.045891 | 0.006901 | ADH4 | 1 |

ADH: alcohol dehydrogenase, *q* value: adjusted *p* value.
