# Supplementary material for: Prognostic implications of alcohol dehydrogenases in hepatocellular carcinoma
Source: BMC Cancer. 2020 Dec 7;20:1204. doi: 10.1186/s12885-020-07689-1 (PMC7720489; doi:10.1186/s12885-020-07689-1)
Supplement: Supplementary file 2 — Additional file 2: Table S2. Enrichment analysis of KEGG pathways for ADHs. [file 12885_2020_7689_MOESM2_ESM.docx]

**Table S2. Enrichment analysis of KEGG pathways for ADHs**

| **KEEG ID** | **Description** | ***p*-value** | ***q*-value** | **Gene ID** | **Count** |
| --- | --- | --- | --- | --- | --- |
| hsa00350 | Tyrosine metabolism | 5.28E-15 | 5.56E-15 | ADH1A/ADH1B/ADH1C/ADH4/ADH5/ADH6 | 6 |
| hsa00071 | Fatty acid degradation | 1.92E-14 | 1.01E-14 | ADH1A/ADH1B/ADH1C/ADH4/ADH5/ADH6 | 6 |
| hsa00830 | Retinol metabolism | 2.71E-13 | 7.81E-14 | ADH1A/ADH1B/ADH1C/ADH4/ADH5/ADH6 | 6 |
| hsa00010 | Glycolysis / Gluconeogenesis | 2.97E-13 | 7.81E-14 | ADH1A/ADH1B/ADH1C/ADH4/ADH5/ADH6 | 6 |
| hsa00982 | Drug metabolism - cytochrome P450 | 4.24E-13 | 8.92E-14 | ADH1A/ADH1B/ADH1C/ADH4/ADH5/ADH6 | 6 |
| hsa00980 | Metabolism of xenobiotics by cytochrome P450 | 5.93E-13 | 1.04E-13 | ADH1A/ADH1B/ADH1C/ADH4/ADH5/ADH6 | 6 |
| hsa05204 | Chemical carcinogenesis | 9.50E-13 | 1.43E-13 | ADH1A/ADH1B/ADH1C/ADH4/ADH5/ADH6 | 6 |

ADH: alcohol dehydrogenase, *q* value: adjusted *p* value.
