# Supplementary material for: Prognostic implications of alcohol dehydrogenases in hepatocellular carcinoma
Source: BMC Cancer. 2020 Dec 7;20:1204. doi: 10.1186/s12885-020-07689-1 (PMC7720489; doi:10.1186/s12885-020-07689-1)
Supplement: Supplementary file 3 — Additional file 3: Table S3. Signaling pathways enriched in HCC samples corresponding ADHs expression by GSEA based on KEGG. [file 12885_2020_7689_MOESM3_ESM.docx]

**Table S3. Signaling pathways enriched in HCC samples corresponding ADHs expression by GSEA based on KEGG.**

| **Signaling pathways enriched in HCC samples corresponding ADH1A expression by GSEA based on KEGG** | | | | | |
| --- | --- | --- | --- | --- | --- |
| **KEGG Name** | **Size** | **ES** | **NES** | **NOM p-value** | **FDR q-value** |
| KEGG_RETINOL_METABOLISM | 64 | 0.7890 | 2.2843 | 0.0000 | 0.0000 |
| KEGG_DRUG_METABOLISM_CYTOCHROME_P450 | 71 | 0.7521 | 2.2284 | 0.0000 | 0.0005 |
| KEGG_FATTY_ACID_METABOLISM | 42 | 0.9048 | 2.2009 | 0.0000 | 0.0003 |
| KEGG_VALINE_LEUCINE_AND_ISOLEUCINE_DEGRADATION | 43 | 0.8611 | 2.1217 | 0.0000 | 0.0004 |
| KEGG_METABOLISM_OF_XENOBIOTICS_BY_CYTOCHROME_P450 | 69 | 0.7126 | 2.1194 | 0.0000 | 0.0004 |
| KEGG_PRIMARY_BILE_ACID_BIOSYNTHESIS | 16 | 0.9267 | 2.0511 | 0.0000 | 0.0007 |
| KEGG_STEROID_HORMONE_BIOSYNTHESIS | 55 | 0.6608 | 1.9860 | 0.0020 | 0.0018 |
| KEGG_BUTANOATE_METABOLISM | 34 | 0.7474 | 1.9624 | 0.0000 | 0.0022 |
| KEGG_ASCORBATE_AND_ALDARATE_METABOLISM | 25 | 0.7746 | 1.9522 | 0.0000 | 0.0025 |
| KEGG_PROPANOATE_METABOLISM | 32 | 0.7648 | 1.9505 | 0.0000 | 0.0024 |
| KEGG_TRYPTOPHAN_METABOLISM | 40 | 0.6809 | 1.9091 | 0.0020 | 0.0039 |
| KEGG_PEROXISOME | 78 | 0.6440 | 1.8863 | 0.0041 | 0.0050 |
| KEGG_TYROSINE_METABOLISM | 42 | 0.5887 | 1.8797 | 0.0020 | 0.0049 |
| KEGG_GLYCINE_SERINE_AND_THREONINE_METABOLISM | 31 | 0.7114 | 1.8584 | 0.0119 | 0.0060 |
| KEGG_PPAR_SIGNALING_PATHWAY | 69 | 0.5638 | 1.8055 | 0.0135 | 0.0100 |
| KEGG_BETA_ALANINE_METABOLISM | 22 | 0.7071 | 1.7900 | 0.0040 | 0.0109 |
| KEGG_COMPLEMENT_AND_COAGULATION_CASCADES | 69 | 0.6287 | 1.7758 | 0.0097 | 0.0122 |
| KEGG_DRUG_METABOLISM_OTHER_ENZYMES | 51 | 0.5444 | 1.7532 | 0.0060 | 0.0146 |
| KEGG_PENTOSE_AND_GLUCURONATE_INTERCONVERSIONS | 28 | 0.6189 | 1.7117 | 0.0156 | 0.0213 |
| KEGG_ARGININE_AND_PROLINE_METABOLISM | 54 | 0.4869 | 1.6137 | 0.0173 | 0.0470 |
| KEGG_STARCH_AND_SUCROSE_METABOLISM | 52 | 0.4803 | 1.5513 | 0.0302 | 0.0729 |
| KEGG_HISTIDINE_METABOLISM | 29 | 0.5205 | 1.5470 | 0.0454 | 0.0715 |
| KEGG_GLYCOLYSIS_GLUCONEOGENESIS | 62 | 0.4575 | 1.5406 | 0.0197 | 0.0713 |
| KEGG_PYRUVATE_METABOLISM | 40 | 0.4969 | 1.5217 | 0.0512 | 0.0777 |
| KEGG_TERPENOID_BACKBONE_BIOSYNTHESIS | 15 | 0.5660 | 1.3993 | 0.1359 | 0.1511 |
| KEGG_ALANINE_ASPARTATE_AND_GLUTAMATE_METABOLISM | 32 | 0.4200 | 1.3510 | 0.1228 | 0.1834 |
| KEGG_BIOSYNTHESIS_OF_UNSATURATED_FATTY_ACIDS | 22 | 0.4983 | 1.3364 | 0.1629 | 0.1886 |
| KEGG_PANTOTHENATE_AND_COA_BIOSYNTHESIS | 16 | 0.4519 | 1.2747 | 0.1700 | 0.2361 |
| KEGG_LINOLEIC_ACID_METABOLISM | 29 | 0.4069 | 1.2532 | 0.1753 | 0.2490 |
| KEGG_PORPHYRIN_AND_CHLOROPHYLL_METABOLISM | 41 | 0.4044 | 1.2282 | 0.2431 | 0.2647 |
| KEGG_GLYOXYLATE_AND_DICARBOXYLATE_METABOLISM | 16 | 0.4446 | 1.2118 | 0.2332 | 0.2721 |
| KEGG_LYSINE_DEGRADATION | 44 | 0.3729 | 1.1197 | 0.3145 | 0.3592 |
| KEGG_ARACHIDONIC_ACID_METABOLISM | 58 | 0.2999 | 1.0932 | 0.2955 | 0.3763 |
| KEGG_PHENYLALANINE_METABOLISM | 18 | 0.3992 | 1.0854 | 0.3516 | 0.3734 |
| KEGG_ONE_CARBON_POOL_BY_FOLATE | 17 | 0.3701 | 1.0702 | 0.3690 | 0.3785 |
| KEGG_STEROID_BIOSYNTHESIS | 17 | 0.3964 | 0.9346 | 0.5282 | 0.5242 |
| KEGG_CITRATE_CYCLE_TCA_CYCLE | 31 | 0.3473 | 0.9041 | 0.5525 | 0.5486 |
| KEGG_ABC_TRANSPORTERS | 44 | 0.2780 | 0.8897 | 0.6151 | 0.5524 |
| KEGG_RENAL_CELL_CARCINOMA | 70 | -0.6692 | -2.0238 | 0.0000 | 0.0342 |
| KEGG_PATHWAYS_IN_CANCER | 325 | -0.5819 | -1.9782 | 0.0000 | 0.0291 |
| KEGG_OOCYTE_MEIOSIS | 112 | -0.6153 | -1.9771 | 0.0000 | 0.0194 |
| KEGG_CELL_CYCLE | 124 | -0.7008 | -1.9601 | 0.0000 | 0.0194 |
| KEGG_ENDOCYTOSIS | 181 | -0.5919 | -1.9560 | 0.0000 | 0.0160 |
| KEGG_SMALL_CELL_LUNG_CANCER | 84 | -0.6384 | -1.9516 | 0.0000 | 0.0140 |
| KEGG_LYSOSOME | 121 | -0.6129 | -1.9448 | 0.0000 | 0.0129 |
| KEGG_PURINE_METABOLISM | 158 | -0.5442 | -1.9362 | 0.0000 | 0.0119 |
| KEGG_VASOPRESSIN_REGULATED_WATER_REABSORPTION | 44 | -0.6743 | -1.9294 | 0.0000 | 0.0111 |
| KEGG_VIBRIO_CHOLERAE_INFECTION | 54 | -0.6305 | -1.9197 | 0.0000 | 0.0114 |
| KEGG_EPITHELIAL_CELL_SIGNALING_IN_HELICOBACTER_PYLORI_INFECTION | 68 | -0.6188 | -1.9160 | 0.0000 | 0.0105 |
| KEGG_VEGF_SIGNALING_PATHWAY | 76 | -0.5833 | -1.9116 | 0.0000 | 0.0101 |
| KEGG_PATHOGENIC_ESCHERICHIA_COLI_INFECTION | 56 | -0.6709 | -1.8980 | 0.0019 | 0.0111 |
| KEGG_FC_GAMMA_R_MEDIATED_PHAGOCYTOSIS | 96 | -0.6601 | -1.8964 | 0.0000 | 0.0105 |
| KEGG_NOTCH_SIGNALING_PATHWAY | 47 | -0.6650 | -1.8954 | 0.0000 | 0.0101 |
| KEGG_AXON_GUIDANCE | 129 | -0.5916 | -1.8921 | 0.0000 | 0.0101 |
| KEGG_REGULATION_OF_ACTIN_CYTOSKELETON | 213 | -0.5794 | -1.8845 | 0.0000 | 0.0101 |
| KEGG_LONG_TERM_DEPRESSION | 70 | -0.5745 | -1.8728 | 0.0000 | 0.0103 |
| KEGG_UBIQUITIN_MEDIATED_PROTEOLYSIS | 134 | -0.6179 | -1.8700 | 0.0020 | 0.0103 |
| KEGG_PANCREATIC_CANCER | 70 | -0.6391 | -1.8638 | 0.0000 | 0.0109 |
| KEGG_AMINOACYL_TRNA_BIOSYNTHESIS | 41 | -0.6797 | -1.8555 | 0.0019 | 0.0122 |
| KEGG_WNT_SIGNALING_PATHWAY | 150 | -0.5574 | -1.8543 | 0.0000 | 0.0118 |
| KEGG_BLADDER_CANCER | 42 | -0.6138 | -1.8524 | 0.0000 | 0.0116 |
| KEGG_CHRONIC_MYELOID_LEUKEMIA | 73 | -0.6192 | -1.8523 | 0.0000 | 0.0111 |
| KEGG_PYRIMIDINE_METABOLISM | 98 | -0.5641 | -1.8519 | 0.0019 | 0.0106 |
| KEGG_INOSITOL_PHOSPHATE_METABOLISM | 54 | -0.6424 | -1.8440 | 0.0020 | 0.0119 |
| KEGG_PHOSPHATIDYLINOSITOL_SIGNALING_SYSTEM | 76 | -0.6201 | -1.8330 | 0.0000 | 0.0138 |
| KEGG_NOD_LIKE_RECEPTOR_SIGNALING_PATHWAY | 62 | -0.6216 | -1.8235 | 0.0038 | 0.0149 |
| KEGG_SNARE_INTERACTIONS_IN_VESICULAR_TRANSPORT | 38 | -0.6344 | -1.8235 | 0.0000 | 0.0144 |
| KEGG_PROGESTERONE_MEDIATED_OOCYTE_MATURATION | 85 | -0.5872 | -1.8203 | 0.0020 | 0.0145 |
| KEGG_NEUROTROPHIN_SIGNALING_PATHWAY | 126 | -0.5988 | -1.8199 | 0.0000 | 0.0140 |
| KEGG_ADHERENS_JUNCTION | 73 | -0.6131 | -1.8150 | 0.0040 | 0.0143 |
| KEGG_GAP_JUNCTION | 90 | -0.5864 | -1.8118 | 0.0000 | 0.0142 |
| KEGG_PROSTATE_CANCER | 89 | -0.5669 | -1.8099 | 0.0000 | 0.0140 |
| KEGG_GLYCEROPHOSPHOLIPID_METABOLISM | 77 | -0.5157 | -1.8073 | 0.0000 | 0.0141 |
| KEGG_FRUCTOSE_AND_MANNOSE_METABOLISM | 33 | -0.5678 | -1.8062 | 0.0020 | 0.0139 |
| KEGG_MTOR_SIGNALING_PATHWAY | 52 | -0.5938 | -1.8050 | 0.0020 | 0.0137 |
| KEGG_ETHER_LIPID_METABOLISM | 33 | -0.5817 | -1.8005 | 0.0000 | 0.0138 |
| KEGG_FOCAL_ADHESION | 199 | -0.5651 | -1.7974 | 0.0041 | 0.0138 |
| KEGG_HOMOLOGOUS_RECOMBINATION | 28 | -0.7286 | -1.7946 | 0.0020 | 0.0139 |
| KEGG_MELANOGENESIS | 101 | -0.5364 | -1.7903 | 0.0000 | 0.0141 |
| KEGG_GLIOMA | 65 | -0.5588 | -1.7892 | 0.0000 | 0.0140 |
| KEGG_THYROID_CANCER | 29 | -0.6451 | -1.7837 | 0.0040 | 0.0146 |
| KEGG_COLORECTAL_CANCER | 62 | -0.5982 | -1.7824 | 0.0059 | 0.0145 |
| KEGG_APOPTOSIS | 87 | -0.5629 | -1.7771 | 0.0039 | 0.0155 |
| KEGG_SPHINGOLIPID_METABOLISM | 39 | -0.5778 | -1.7751 | 0.0018 | 0.0154 |
| KEGG_ERBB_SIGNALING_PATHWAY | 87 | -0.5616 | -1.7749 | 0.0000 | 0.0151 |
| KEGG_FC_EPSILON_RI_SIGNALING_PATHWAY | 79 | -0.5534 | -1.7666 | 0.0039 | 0.0167 |
| KEGG_MAPK_SIGNALING_PATHWAY | 267 | -0.5071 | -1.7647 | 0.0000 | 0.0165 |
| KEGG_GNRH_SIGNALING_PATHWAY | 101 | -0.5432 | -1.7625 | 0.0000 | 0.0167 |
| KEGG_TGF_BETA_SIGNALING_PATHWAY | 85 | -0.5696 | -1.7625 | 0.0081 | 0.0163 |
| KEGG_SPLICEOSOME | 127 | -0.6804 | -1.7602 | 0.0000 | 0.0164 |
| KEGG_NON_SMALL_CELL_LUNG_CANCER | 54 | -0.5727 | -1.7565 | 0.0020 | 0.0168 |
| KEGG_P53_SIGNALING_PATHWAY | 68 | -0.5312 | -1.7557 | 0.0095 | 0.0166 |
| KEGG_RIG_I_LIKE_RECEPTOR_SIGNALING_PATHWAY | 71 | -0.5413 | -1.7542 | 0.0095 | 0.0165 |
| KEGG_HYPERTROPHIC_CARDIOMYOPATHY_HCM | 83 | -0.5586 | -1.7534 | 0.0000 | 0.0163 |
| KEGG_GLYCOSPHINGOLIPID_BIOSYNTHESIS_LACTO_AND_NEOLACTO_SERIES | 26 | -0.5964 | -1.7488 | 0.0038 | 0.0168 |
| KEGG_GLYCOSAMINOGLYCAN_BIOSYNTHESIS_CHONDROITIN_SULFATE | 22 | -0.6654 | -1.7459 | 0.0079 | 0.0172 |
| KEGG_ACUTE_MYELOID_LEUKEMIA | 57 | -0.5705 | -1.7438 | 0.0060 | 0.0174 |
| KEGG_INSULIN_SIGNALING_PATHWAY | 137 | -0.4999 | -1.7425 | 0.0020 | 0.0174 |
| KEGG_N_GLYCAN_BIOSYNTHESIS | 46 | -0.5873 | -1.7415 | 0.0152 | 0.0173 |
| KEGG_CYTOSOLIC_DNA_SENSING_PATHWAY | 55 | -0.5623 | -1.7411 | 0.0059 | 0.0171 |
| KEGG_LONG_TERM_POTENTIATION | 70 | -0.5330 | -1.7273 | 0.0058 | 0.0191 |
| KEGG_MISMATCH_REPAIR | 23 | -0.7422 | -1.7273 | 0.0040 | 0.0188 |
| KEGG_ARRHYTHMOGENIC_RIGHT_VENTRICULAR_CARDIOMYOPATHY_ARVC | 74 | -0.5624 | -1.7119 | 0.0082 | 0.0213 |
| KEGG_DILATED_CARDIOMYOPATHY | 90 | -0.5547 | -1.7076 | 0.0040 | 0.0220 |
| KEGG_RNA_POLYMERASE | 29 | -0.6435 | -1.7065 | 0.0113 | 0.0219 |
| KEGG_GLYCOSAMINOGLYCAN_BIOSYNTHESIS_KERATAN_SULFATE | 15 | -0.6730 | -1.7002 | 0.0082 | 0.0231 |
| KEGG_BASE_EXCISION_REPAIR | 35 | -0.6163 | -1.6991 | 0.0171 | 0.0230 |
| KEGG_DNA_REPLICATION | 36 | -0.7270 | -1.6940 | 0.0139 | 0.0238 |
| KEGG_RNA_DEGRADATION | 59 | -0.6037 | -1.6934 | 0.0104 | 0.0236 |
| KEGG_TOLL_LIKE_RECEPTOR_SIGNALING_PATHWAY | 102 | -0.5206 | -1.6919 | 0.0209 | 0.0235 |
| KEGG_CHEMOKINE_SIGNALING_PATHWAY | 188 | -0.5310 | -1.6894 | 0.0176 | 0.0235 |
| KEGG_MELANOMA | 71 | -0.5099 | -1.6774 | 0.0040 | 0.0256 |
| KEGG_ENDOMETRIAL_CANCER | 52 | -0.5535 | -1.6767 | 0.0141 | 0.0255 |
| KEGG_JAK_STAT_SIGNALING_PATHWAY | 155 | -0.5037 | -1.6736 | 0.0138 | 0.0259 |
| KEGG_BASAL_CELL_CARCINOMA | 55 | -0.5340 | -1.6688 | 0.0020 | 0.0265 |
| KEGG_TIGHT_JUNCTION | 132 | -0.4894 | -1.6675 | 0.0099 | 0.0267 |
| KEGG_LEUKOCYTE_TRANSENDOTHELIAL_MIGRATION | 116 | -0.5142 | -1.6506 | 0.0180 | 0.0308 |
| KEGG_NATURAL_KILLER_CELL_MEDIATED_CYTOTOXICITY | 132 | -0.5241 | -1.6322 | 0.0295 | 0.0360 |
| KEGG_T_CELL_RECEPTOR_SIGNALING_PATHWAY | 108 | -0.5603 | -1.6174 | 0.0282 | 0.0400 |
| KEGG_BASAL_TRANSCRIPTION_FACTORS | 35 | -0.5980 | -1.6168 | 0.0222 | 0.0398 |
| KEGG_NUCLEOTIDE_EXCISION_REPAIR | 44 | -0.5893 | -1.6147 | 0.0356 | 0.0399 |
| KEGG_PENTOSE_PHOSPHATE_PATHWAY | 27 | -0.5421 | -1.6120 | 0.0255 | 0.0404 |
| KEGG_SELENOAMINO_ACID_METABOLISM | 26 | -0.5380 | -1.6070 | 0.0245 | 0.0416 |
| KEGG_B_CELL_RECEPTOR_SIGNALING_PATHWAY | 75 | -0.5550 | -1.6023 | 0.0323 | 0.0426 |
| KEGG_HEDGEHOG_SIGNALING_PATHWAY | 56 | -0.5153 | -1.6022 | 0.0100 | 0.0422 |
| KEGG_VASCULAR_SMOOTH_MUSCLE_CONTRACTION | 115 | -0.4842 | -1.6011 | 0.0243 | 0.0420 |
| KEGG_HUNTINGTONS_DISEASE | 180 | -0.4795 | -1.5989 | 0.0544 | 0.0423 |
| KEGG_CALCIUM_SIGNALING_PATHWAY | 177 | -0.4456 | -1.5829 | 0.0059 | 0.0475 |
| KEGG_DORSO_VENTRAL_AXIS_FORMATION | 24 | -0.5846 | -1.5701 | 0.0447 | 0.0518 |
| KEGG_RIBOFLAVIN_METABOLISM | 16 | -0.5888 | -1.5637 | 0.0239 | 0.0534 |
| KEGG_ECM_RECEPTOR_INTERACTION | 84 | -0.5015 | -1.5449 | 0.0671 | 0.0605 |
| KEGG_AMYOTROPHIC_LATERAL_SCLEROSIS_ALS | 53 | -0.4358 | -1.5270 | 0.0300 | 0.0672 |
| KEGG_CARDIAC_MUSCLE_CONTRACTION | 78 | -0.4749 | -1.5262 | 0.0412 | 0.0668 |
| KEGG_GLYCOSAMINOGLYCAN_BIOSYNTHESIS_HEPARAN_SULFATE | 26 | -0.5352 | -1.5232 | 0.0383 | 0.0672 |
| KEGG_ALZHEIMERS_DISEASE | 165 | -0.4463 | -1.5228 | 0.0593 | 0.0667 |
| KEGG_LEISHMANIA_INFECTION | 70 | -0.5633 | -1.5187 | 0.0615 | 0.0681 |
| KEGG_TYPE_II_DIABETES_MELLITUS | 47 | -0.4773 | -1.5087 | 0.0392 | 0.0723 |
| KEGG_O_GLYCAN_BIOSYNTHESIS | 30 | -0.5426 | -1.4942 | 0.0567 | 0.0783 |
| KEGG_GLYCOSYLPHOSPHATIDYLINOSITOL_GPI_ANCHOR_BIOSYNTHESIS | 25 | -0.5531 | -1.4843 | 0.0567 | 0.0830 |
| KEGG_AMINO_SUGAR_AND_NUCLEOTIDE_SUGAR_METABOLISM | 43 | -0.4723 | -1.4837 | 0.0562 | 0.0825 |
| KEGG_ALDOSTERONE_REGULATED_SODIUM_REABSORPTION | 42 | -0.4539 | -1.4718 | 0.0417 | 0.0875 |
| KEGG_REGULATION_OF_AUTOPHAGY | 35 | -0.4678 | -1.4640 | 0.0706 | 0.0906 |
| KEGG_CYTOKINE_CYTOKINE_RECEPTOR_INTERACTION | 264 | -0.4395 | -1.4582 | 0.0837 | 0.0929 |
| KEGG_OTHER_GLYCAN_DEGRADATION | 16 | -0.5611 | -1.4558 | 0.0751 | 0.0936 |
| KEGG_GLYCOSPHINGOLIPID_BIOSYNTHESIS_GANGLIO_SERIES | 15 | -0.5500 | -1.4506 | 0.0882 | 0.0959 |
| KEGG_PRION_DISEASES | 35 | -0.4769 | -1.4449 | 0.0798 | 0.0985 |
| KEGG_PROTEIN_EXPORT | 24 | -0.5185 | -1.3766 | 0.1269 | 0.1391 |
| KEGG_CYSTEINE_AND_METHIONINE_METABOLISM | 34 | -0.4252 | -1.3684 | 0.1016 | 0.1427 |
| KEGG_TASTE_TRANSDUCTION | 51 | -0.4299 | -1.3669 | 0.0760 | 0.1425 |
| KEGG_NEUROACTIVE_LIGAND_RECEPTOR_INTERACTION | 271 | -0.3491 | -1.3164 | 0.0640 | 0.1787 |
| KEGG_GLYCOSAMINOGLYCAN_DEGRADATION | 21 | -0.4670 | -1.2853 | 0.1650 | 0.2014 |
| KEGG_PROTEASOME | 46 | -0.4756 | -1.2398 | 0.2724 | 0.2394 |
| KEGG_VIRAL_MYOCARDITIS | 68 | -0.4270 | -1.2286 | 0.2422 | 0.2485 |
| KEGG_HEMATOPOIETIC_CELL_LINEAGE | 85 | -0.4128 | -1.2050 | 0.2672 | 0.2696 |
| KEGG_OXIDATIVE_PHOSPHORYLATION | 131 | -0.3999 | -1.1670 | 0.3131 | 0.3049 |
| KEGG_CELL_ADHESION_MOLECULES_CAMS | 131 | -0.3745 | -1.1628 | 0.2904 | 0.3059 |
| KEGG_ANTIGEN_PROCESSING_AND_PRESENTATION | 81 | -0.4202 | -1.1595 | 0.3392 | 0.3069 |
| KEGG_GALACTOSE_METABOLISM | 26 | -0.3726 | -1.1539 | 0.2584 | 0.3102 |
| KEGG_SYSTEMIC_LUPUS_ERYTHEMATOSUS | 135 | -0.3501 | -1.1528 | 0.2877 | 0.3091 |
| KEGG_ADIPOCYTOKINE_SIGNALING_PATHWAY | 67 | -0.3297 | -1.1374 | 0.2817 | 0.3231 |
| KEGG_ALPHA_LINOLENIC_ACID_METABOLISM | 19 | -0.3852 | -1.1130 | 0.2910 | 0.3469 |
| KEGG_GLUTATHIONE_METABOLISM | 49 | -0.3369 | -1.1026 | 0.3198 | 0.3558 |
| KEGG_PARKINSONS_DISEASE | 128 | -0.3649 | -1.0967 | 0.3686 | 0.3597 |
| KEGG_GLYCEROLIPID_METABOLISM | 49 | -0.3114 | -1.0892 | 0.3307 | 0.3648 |
| KEGG_RIBOSOME | 87 | -0.5132 | -1.0324 | 0.4990 | 0.4261 |
| KEGG_NITROGEN_METABOLISM | 23 | -0.3446 | -1.0077 | 0.4192 | 0.4527 |
| KEGG_INTESTINAL_IMMUNE_NETWORK_FOR_IGA_PRODUCTION | 46 | -0.3755 | -0.9439 | 0.5193 | 0.5305 |
| KEGG_NICOTINATE_AND_NICOTINAMIDE_METABOLISM | 24 | -0.3185 | -0.9437 | 0.5412 | 0.5266 |
| KEGG_TYPE_I_DIABETES_MELLITUS | 41 | -0.3838 | -0.9420 | 0.5467 | 0.5250 |
| KEGG_MATURITY_ONSET_DIABETES_OF_THE_YOUNG | 25 | -0.3251 | -0.9394 | 0.5286 | 0.5242 |
| KEGG_PROXIMAL_TUBULE_BICARBONATE_RECLAMATION | 23 | -0.3113 | -0.9327 | 0.5458 | 0.5289 |
| KEGG_PRIMARY_IMMUNODEFICIENCY | 35 | -0.4011 | -0.9192 | 0.5800 | 0.5424 |
| KEGG_RENIN_ANGIOTENSIN_SYSTEM | 17 | -0.3392 | -0.8615 | 0.6543 | 0.6151 |
| KEGG_OLFACTORY_TRANSDUCTION | 383 | -0.1837 | -0.8590 | 0.8255 | 0.6140 |
| KEGG_GRAFT_VERSUS_HOST_DISEASE | 37 | -0.3457 | -0.7718 | 0.6951 | 0.7275 |
| KEGG_AUTOIMMUNE_THYROID_DISEASE | 50 | -0.2796 | -0.7222 | 0.7264 | 0.7894 |
| KEGG_ALLOGRAFT_REJECTION | 35 | -0.2935 | -0.6652 | 0.7665 | 0.8565 |
| KEGG_ASTHMA | 28 | -0.2141 | -0.5346 | 0.9323 | 0.9643 |

| **Signaling pathways enriched in HCC samples corresponding ADH1B expression by GSEA based on KEGG** | | | | | |
| --- | --- | --- | --- | --- | --- |
| **KEGG Name** | **Size** | **ES** | **NES** | **NOM p-value** | **FDR q-value** |
| KEGG_RETINOL_METABOLISM | 64 | 0.7959 | 2.4247 | 0.0000 | 0.0000 |
| KEGG_DRUG_METABOLISM_CYTOCHROME_P450 | 71 | 0.7895 | 2.3977 | 0.0000 | 0.0000 |
| KEGG_METABOLISM_OF_XENOBIOTICS_BY_CYTOCHROME_P450 | 69 | 0.7515 | 2.3443 | 0.0000 | 0.0005 |
| KEGG_STEROID_HORMONE_BIOSYNTHESIS | 55 | 0.7291 | 2.3043 | 0.0000 | 0.0004 |
| KEGG_TRYPTOPHAN_METABOLISM | 40 | 0.7765 | 2.2575 | 0.0000 | 0.0003 |
| KEGG_FATTY_ACID_METABOLISM | 42 | 0.8963 | 2.2362 | 0.0000 | 0.0002 |
| KEGG_VALINE_LEUCINE_AND_ISOLEUCINE_DEGRADATION | 43 | 0.8820 | 2.2253 | 0.0000 | 0.0003 |
| KEGG_PPAR_SIGNALING_PATHWAY | 69 | 0.6546 | 2.1760 | 0.0000 | 0.0005 |
| KEGG_PEROXISOME | 78 | 0.6995 | 2.1672 | 0.0000 | 0.0004 |
| KEGG_TYROSINE_METABOLISM | 42 | 0.6769 | 2.1627 | 0.0000 | 0.0004 |
| KEGG_PROPANOATE_METABOLISM | 32 | 0.8087 | 2.1175 | 0.0000 | 0.0004 |
| KEGG_GLYCINE_SERINE_AND_THREONINE_METABOLISM | 31 | 0.7899 | 2.1138 | 0.0000 | 0.0004 |
| KEGG_PRIMARY_BILE_ACID_BIOSYNTHESIS | 16 | 0.9221 | 2.0951 | 0.0000 | 0.0004 |
| KEGG_BUTANOATE_METABOLISM | 34 | 0.7591 | 2.0830 | 0.0000 | 0.0004 |
| KEGG_BETA_ALANINE_METABOLISM | 22 | 0.7964 | 2.0752 | 0.0020 | 0.0004 |
| KEGG_COMPLEMENT_AND_COAGULATION_CASCADES | 69 | 0.7219 | 1.9913 | 0.0020 | 0.0017 |
| KEGG_ASCORBATE_AND_ALDARATE_METABOLISM | 25 | 0.7671 | 1.9538 | 0.0020 | 0.0023 |
| KEGG_HISTIDINE_METABOLISM | 29 | 0.6129 | 1.8733 | 0.0080 | 0.0056 |
| KEGG_DRUG_METABOLISM_OTHER_ENZYMES | 51 | 0.5836 | 1.8725 | 0.0041 | 0.0054 |
| KEGG_PYRUVATE_METABOLISM | 40 | 0.5887 | 1.8707 | 0.0021 | 0.0052 |
| KEGG_STARCH_AND_SUCROSE_METABOLISM | 52 | 0.5618 | 1.8464 | 0.0020 | 0.0066 |
| KEGG_ARGININE_AND_PROLINE_METABOLISM | 54 | 0.5400 | 1.8183 | 0.0000 | 0.0080 |
| KEGG_PENTOSE_AND_GLUCURONATE_INTERCONVERSIONS | 28 | 0.6585 | 1.7993 | 0.0061 | 0.0098 |
| KEGG_GLYCOLYSIS_GLUCONEOGENESIS | 62 | 0.5123 | 1.7747 | 0.0044 | 0.0120 |
| KEGG_PHENYLALANINE_METABOLISM | 18 | 0.6375 | 1.7631 | 0.0095 | 0.0129 |
| KEGG_PORPHYRIN_AND_CHLOROPHYLL_METABOLISM | 41 | 0.5459 | 1.6744 | 0.0174 | 0.0284 |
| KEGG_LINOLEIC_ACID_METABOLISM | 29 | 0.5499 | 1.6621 | 0.0062 | 0.0307 |
| KEGG_GLYOXYLATE_AND_DICARBOXYLATE_METABOLISM | 16 | 0.5803 | 1.6196 | 0.0193 | 0.0416 |
| KEGG_CITRATE_CYCLE_TCA_CYCLE | 31 | 0.5960 | 1.6195 | 0.0524 | 0.0402 |
| KEGG_ALANINE_ASPARTATE_AND_GLUTAMATE_METABOLISM | 32 | 0.4904 | 1.6077 | 0.0150 | 0.0431 |
| KEGG_LYSINE_DEGRADATION | 44 | 0.5115 | 1.5811 | 0.0508 | 0.0498 |
| KEGG_BIOSYNTHESIS_OF_UNSATURATED_FATTY_ACIDS | 22 | 0.5671 | 1.5540 | 0.0421 | 0.0587 |
| KEGG_ARACHIDONIC_ACID_METABOLISM | 58 | 0.4211 | 1.5081 | 0.0136 | 0.0768 |
| KEGG_ABC_TRANSPORTERS | 44 | 0.4469 | 1.4889 | 0.0408 | 0.0836 |
| KEGG_ADIPOCYTOKINE_SIGNALING_PATHWAY | 67 | 0.4030 | 1.4443 | 0.0589 | 0.1047 |
| KEGG_NITROGEN_METABOLISM | 23 | 0.4743 | 1.4157 | 0.0852 | 0.1175 |
| KEGG_GLYCEROLIPID_METABOLISM | 49 | 0.3713 | 1.3439 | 0.0813 | 0.1604 |
| KEGG_CYSTEINE_AND_METHIONINE_METABOLISM | 34 | 0.4047 | 1.3081 | 0.1206 | 0.1828 |
| KEGG_ONE_CARBON_POOL_BY_FOLATE | 17 | 0.4410 | 1.2863 | 0.1603 | 0.1947 |
| KEGG_NICOTINATE_AND_NICOTINAMIDE_METABOLISM | 24 | 0.3417 | 1.0685 | 0.3516 | 0.4020 |
| KEGG_PROXIMAL_TUBULE_BICARBONATE_RECLAMATION | 23 | 0.3149 | 0.9354 | 0.5346 | 0.5592 |
| KEGG_PANTOTHENATE_AND_COA_BIOSYNTHESIS | 16 | 0.3283 | 0.9349 | 0.5373 | 0.5465 |
| KEGG_RENIN_ANGIOTENSIN_SYSTEM | 17 | 0.3576 | 0.9325 | 0.5536 | 0.5370 |
| KEGG_STEROID_BIOSYNTHESIS | 17 | 0.3637 | 0.8341 | 0.6318 | 0.6583 |
| KEGG_GALACTOSE_METABOLISM | 26 | 0.2570 | 0.8035 | 0.7426 | 0.6865 |
| KEGG_TERPENOID_BACKBONE_BIOSYNTHESIS | 15 | 0.3070 | 0.7572 | 0.7265 | 0.7356 |
| KEGG_PATHOGENIC_ESCHERICHIA_COLI_INFECTION | 56 | -0.6867 | -2.0268 | 0.0000 | 0.0212 |
| KEGG_SPLICEOSOME | 127 | -0.7456 | -1.9875 | 0.0000 | 0.0228 |
| KEGG_PURINE_METABOLISM | 158 | -0.5396 | -1.9542 | 0.0000 | 0.0317 |
| KEGG_VEGF_SIGNALING_PATHWAY | 76 | -0.5685 | -1.9298 | 0.0000 | 0.0306 |
| KEGG_NOTCH_SIGNALING_PATHWAY | 47 | -0.6588 | -1.9079 | 0.0000 | 0.0338 |
| KEGG_RENAL_CELL_CARCINOMA | 70 | -0.6261 | -1.9065 | 0.0000 | 0.0289 |
| KEGG_REGULATION_OF_ACTIN_CYTOSKELETON | 213 | -0.5554 | -1.8671 | 0.0000 | 0.0375 |
| KEGG_PYRIMIDINE_METABOLISM | 98 | -0.5663 | -1.8669 | 0.0041 | 0.0328 |
| KEGG_FC_GAMMA_R_MEDIATED_PHAGOCYTOSIS | 96 | -0.6215 | -1.8657 | 0.0000 | 0.0297 |
| KEGG_OOCYTE_MEIOSIS | 112 | -0.5844 | -1.8589 | 0.0020 | 0.0278 |
| KEGG_ENDOCYTOSIS | 181 | -0.5558 | -1.8479 | 0.0000 | 0.0293 |
| KEGG_PATHWAYS_IN_CANCER | 325 | -0.5303 | -1.8389 | 0.0000 | 0.0292 |
| KEGG_CELL_CYCLE | 124 | -0.6520 | -1.8145 | 0.0021 | 0.0389 |
| KEGG_VIBRIO_CHOLERAE_INFECTION | 54 | -0.5778 | -1.8108 | 0.0040 | 0.0385 |
| KEGG_DNA_REPLICATION | 36 | -0.7994 | -1.8071 | 0.0103 | 0.0378 |
| KEGG_BLADDER_CANCER | 42 | -0.5901 | -1.7964 | 0.0020 | 0.0395 |
| KEGG_HOMOLOGOUS_RECOMBINATION | 28 | -0.7361 | -1.7886 | 0.0021 | 0.0400 |
| KEGG_RNA_DEGRADATION | 59 | -0.6196 | -1.7851 | 0.0020 | 0.0395 |
| KEGG_BASE_EXCISION_REPAIR | 35 | -0.6682 | -1.7832 | 0.0040 | 0.0382 |
| KEGG_PROGESTERONE_MEDIATED_OOCYTE_MATURATION | 85 | -0.5600 | -1.7685 | 0.0000 | 0.0424 |
| KEGG_P53_SIGNALING_PATHWAY | 68 | -0.5329 | -1.7525 | 0.0058 | 0.0476 |
| KEGG_GLYCEROPHOSPHOLIPID_METABOLISM | 77 | -0.4770 | -1.7472 | 0.0020 | 0.0475 |
| KEGG_NUCLEOTIDE_EXCISION_REPAIR | 44 | -0.6409 | -1.7429 | 0.0118 | 0.0471 |
| KEGG_AXON_GUIDANCE | 129 | -0.5443 | -1.7392 | 0.0021 | 0.0467 |
| KEGG_DORSO_VENTRAL_AXIS_FORMATION | 24 | -0.6432 | -1.7392 | 0.0040 | 0.0450 |
| KEGG_VASOPRESSIN_REGULATED_WATER_REABSORPTION | 44 | -0.5985 | -1.7303 | 0.0062 | 0.0469 |
| KEGG_CHRONIC_MYELOID_LEUKEMIA | 73 | -0.5644 | -1.7299 | 0.0098 | 0.0454 |
| KEGG_RIBOFLAVIN_METABOLISM | 16 | -0.6473 | -1.7291 | 0.0098 | 0.0442 |
| KEGG_SNARE_INTERACTIONS_IN_VESICULAR_TRANSPORT | 38 | -0.5877 | -1.7286 | 0.0083 | 0.0427 |
| KEGG_UBIQUITIN_MEDIATED_PROTEOLYSIS | 134 | -0.5802 | -1.7233 | 0.0081 | 0.0438 |
| KEGG_GNRH_SIGNALING_PATHWAY | 101 | -0.5158 | -1.7024 | 0.0040 | 0.0520 |
| KEGG_LONG_TERM_DEPRESSION | 70 | -0.5105 | -1.6997 | 0.0061 | 0.0514 |
| KEGG_N_GLYCAN_BIOSYNTHESIS | 46 | -0.5693 | -1.6985 | 0.0164 | 0.0503 |
| KEGG_MISMATCH_REPAIR | 23 | -0.7216 | -1.6928 | 0.0098 | 0.0512 |
| KEGG_NEUROTROPHIN_SIGNALING_PATHWAY | 126 | -0.5392 | -1.6927 | 0.0120 | 0.0498 |
| KEGG_FC_EPSILON_RI_SIGNALING_PATHWAY | 79 | -0.5156 | -1.6926 | 0.0057 | 0.0484 |
| KEGG_GLIOMA | 65 | -0.5267 | -1.6923 | 0.0080 | 0.0471 |
| KEGG_SMALL_CELL_LUNG_CANCER | 84 | -0.5395 | -1.6920 | 0.0163 | 0.0460 |
| KEGG_LYSOSOME | 121 | -0.5197 | -1.6845 | 0.0146 | 0.0472 |
| KEGG_HUNTINGTONS_DISEASE | 180 | -0.5006 | -1.6832 | 0.0239 | 0.0467 |
| KEGG_CARDIAC_MUSCLE_CONTRACTION | 78 | -0.5093 | -1.6768 | 0.0134 | 0.0477 |
| KEGG_MTOR_SIGNALING_PATHWAY | 52 | -0.5446 | -1.6763 | 0.0080 | 0.0468 |
| KEGG_BASAL_TRANSCRIPTION_FACTORS | 35 | -0.6117 | -1.6763 | 0.0117 | 0.0457 |
| KEGG_WNT_SIGNALING_PATHWAY | 150 | -0.5096 | -1.6737 | 0.0083 | 0.0456 |
| KEGG_GLYCOSAMINOGLYCAN_BIOSYNTHESIS_HEPARAN_SULFATE | 26 | -0.6022 | -1.6676 | 0.0063 | 0.0467 |
| KEGG_MELANOMA | 71 | -0.5085 | -1.6640 | 0.0101 | 0.0470 |
| KEGG_ADHERENS_JUNCTION | 73 | -0.5578 | -1.6606 | 0.0176 | 0.0474 |
| KEGG_PANCREATIC_CANCER | 70 | -0.5607 | -1.6556 | 0.0205 | 0.0487 |
| KEGG_MAPK_SIGNALING_PATHWAY | 267 | -0.4674 | -1.6372 | 0.0082 | 0.0557 |
| KEGG_NON_SMALL_CELL_LUNG_CANCER | 54 | -0.5287 | -1.6359 | 0.0216 | 0.0550 |
| KEGG_ERBB_SIGNALING_PATHWAY | 87 | -0.5166 | -1.6352 | 0.0240 | 0.0544 |
| KEGG_INOSITOL_PHOSPHATE_METABOLISM | 54 | -0.5569 | -1.6320 | 0.0304 | 0.0544 |
| KEGG_PHOSPHATIDYLINOSITOL_SIGNALING_SYSTEM | 76 | -0.5434 | -1.6305 | 0.0207 | 0.0538 |
| KEGG_PROTEIN_EXPORT | 24 | -0.6069 | -1.6274 | 0.0392 | 0.0547 |
| KEGG_TIGHT_JUNCTION | 132 | -0.4743 | -1.6259 | 0.0161 | 0.0543 |
| KEGG_GAP_JUNCTION | 90 | -0.5154 | -1.6247 | 0.0204 | 0.0538 |
| KEGG_NOD_LIKE_RECEPTOR_SIGNALING_PATHWAY | 62 | -0.5424 | -1.6219 | 0.0366 | 0.0540 |
| KEGG_COLORECTAL_CANCER | 62 | -0.5443 | -1.6195 | 0.0259 | 0.0541 |
| KEGG_CYTOSOLIC_DNA_SENSING_PATHWAY | 55 | -0.5293 | -1.6101 | 0.0190 | 0.0573 |
| KEGG_FOCAL_ADHESION | 199 | -0.4937 | -1.6095 | 0.0384 | 0.0568 |
| KEGG_THYROID_CANCER | 29 | -0.5686 | -1.5997 | 0.0293 | 0.0604 |
| KEGG_RNA_POLYMERASE | 29 | -0.6068 | -1.5996 | 0.0290 | 0.0595 |
| KEGG_MELANOGENESIS | 101 | -0.4748 | -1.5980 | 0.0120 | 0.0593 |
| KEGG_GLYCOSAMINOGLYCAN_BIOSYNTHESIS_CHONDROITIN_SULFATE | 22 | -0.6108 | -1.5866 | 0.0246 | 0.0645 |
| KEGG_ETHER_LIPID_METABOLISM | 33 | -0.4934 | -1.5856 | 0.0259 | 0.0639 |
| KEGG_RIBOSOME | 87 | -0.7559 | -1.5839 | 0.0526 | 0.0638 |
| KEGG_LONG_TERM_POTENTIATION | 70 | -0.4873 | -1.5824 | 0.0188 | 0.0634 |
| KEGG_EPITHELIAL_CELL_SIGNALING_IN_HELICOBACTER_PYLORI_INFECTION | 68 | -0.5069 | -1.5799 | 0.0346 | 0.0640 |
| KEGG_ALZHEIMERS_DISEASE | 165 | -0.4527 | -1.5761 | 0.0554 | 0.0647 |
| KEGG_TGF_BETA_SIGNALING_PATHWAY | 85 | -0.4958 | -1.5671 | 0.0316 | 0.0680 |
| KEGG_LEUKOCYTE_TRANSENDOTHELIAL_MIGRATION | 116 | -0.4838 | -1.5645 | 0.0388 | 0.0681 |
| KEGG_HYPERTROPHIC_CARDIOMYOPATHY_HCM | 83 | -0.4854 | -1.5561 | 0.0417 | 0.0712 |
| KEGG_AMINOACYL_TRNA_BIOSYNTHESIS | 41 | -0.5805 | -1.5483 | 0.0675 | 0.0744 |
| KEGG_GLYCOSPHINGOLIPID_BIOSYNTHESIS_LACTO_AND_NEOLACTO_SERIES | 26 | -0.5050 | -1.5420 | 0.0292 | 0.0772 |
| KEGG_PROSTATE_CANCER | 89 | -0.4736 | -1.5392 | 0.0378 | 0.0775 |
| KEGG_T_CELL_RECEPTOR_SIGNALING_PATHWAY | 108 | -0.5209 | -1.5211 | 0.0673 | 0.0866 |
| KEGG_TOLL_LIKE_RECEPTOR_SIGNALING_PATHWAY | 102 | -0.4762 | -1.5158 | 0.0578 | 0.0882 |
| KEGG_RIG_I_LIKE_RECEPTOR_SIGNALING_PATHWAY | 71 | -0.4658 | -1.5122 | 0.0621 | 0.0893 |
| KEGG_ACUTE_MYELOID_LEUKEMIA | 57 | -0.4950 | -1.5116 | 0.0655 | 0.0885 |
| KEGG_DILATED_CARDIOMYOPATHY | 90 | -0.4797 | -1.5082 | 0.0579 | 0.0890 |
| KEGG_CHEMOKINE_SIGNALING_PATHWAY | 188 | -0.4673 | -1.5032 | 0.0758 | 0.0907 |
| KEGG_JAK_STAT_SIGNALING_PATHWAY | 155 | -0.4437 | -1.4996 | 0.0736 | 0.0916 |
| KEGG_AMYOTROPHIC_LATERAL_SCLEROSIS_ALS | 53 | -0.4228 | -1.4848 | 0.0321 | 0.0986 |
| KEGG_B_CELL_RECEPTOR_SIGNALING_PATHWAY | 75 | -0.5050 | -1.4808 | 0.0751 | 0.0996 |
| KEGG_APOPTOSIS | 87 | -0.4652 | -1.4785 | 0.0716 | 0.0999 |
| KEGG_ENDOMETRIAL_CANCER | 52 | -0.4782 | -1.4749 | 0.0871 | 0.1007 |
| KEGG_LEISHMANIA_INFECTION | 70 | -0.5340 | -1.4708 | 0.1064 | 0.1018 |
| KEGG_NATURAL_KILLER_CELL_MEDIATED_CYTOTOXICITY | 132 | -0.4766 | -1.4643 | 0.1076 | 0.1048 |
| KEGG_FRUCTOSE_AND_MANNOSE_METABOLISM | 33 | -0.4516 | -1.4574 | 0.0591 | 0.1077 |
| KEGG_GLYCOSYLPHOSPHATIDYLINOSITOL_GPI_ANCHOR_BIOSYNTHESIS | 25 | -0.5261 | -1.4343 | 0.0990 | 0.1210 |
| KEGG_INSULIN_SIGNALING_PATHWAY | 137 | -0.3938 | -1.4136 | 0.1042 | 0.1337 |
| KEGG_VASCULAR_SMOOTH_MUSCLE_CONTRACTION | 115 | -0.4156 | -1.4063 | 0.1200 | 0.1371 |
| KEGG_SPHINGOLIPID_METABOLISM | 39 | -0.4630 | -1.3905 | 0.1060 | 0.1469 |
| KEGG_ARRHYTHMOGENIC_RIGHT_VENTRICULAR_CARDIOMYOPATHY_ARVC | 74 | -0.4441 | -1.3859 | 0.1049 | 0.1484 |
| KEGG_CYTOKINE_CYTOKINE_RECEPTOR_INTERACTION | 264 | -0.4102 | -1.3762 | 0.1542 | 0.1536 |
| KEGG_ANTIGEN_PROCESSING_AND_PRESENTATION | 81 | -0.5037 | -1.3568 | 0.1779 | 0.1670 |
| KEGG_BASAL_CELL_CARCINOMA | 55 | -0.4387 | -1.3557 | 0.0793 | 0.1661 |
| KEGG_TASTE_TRANSDUCTION | 51 | -0.4216 | -1.3526 | 0.1010 | 0.1666 |
| KEGG_AMINO_SUGAR_AND_NUCLEOTIDE_SUGAR_METABOLISM | 43 | -0.4223 | -1.3495 | 0.1263 | 0.1672 |
| KEGG_GLYCOSAMINOGLYCAN_BIOSYNTHESIS_KERATAN_SULFATE | 15 | -0.5221 | -1.3422 | 0.1337 | 0.1711 |
| KEGG_PROTEASOME | 46 | -0.5168 | -1.3298 | 0.1932 | 0.1788 |
| KEGG_PRION_DISEASES | 35 | -0.4203 | -1.3189 | 0.1429 | 0.1851 |
| KEGG_HEDGEHOG_SIGNALING_PATHWAY | 56 | -0.4228 | -1.3005 | 0.1467 | 0.1992 |
| KEGG_CALCIUM_SIGNALING_PATHWAY | 177 | -0.3647 | -1.2950 | 0.1511 | 0.2017 |
| KEGG_TYPE_II_DIABETES_MELLITUS | 47 | -0.4071 | -1.2948 | 0.1561 | 0.1999 |
| KEGG_ECM_RECEPTOR_INTERACTION | 84 | -0.4143 | -1.2777 | 0.2008 | 0.2128 |
| KEGG_VIRAL_MYOCARDITIS | 68 | -0.4324 | -1.2706 | 0.2311 | 0.2167 |
| KEGG_CELL_ADHESION_MOLECULES_CAMS | 131 | -0.4040 | -1.2562 | 0.2344 | 0.2269 |
| KEGG_REGULATION_OF_AUTOPHAGY | 35 | -0.3998 | -1.2410 | 0.1896 | 0.2378 |
| KEGG_GLUTATHIONE_METABOLISM | 49 | -0.3679 | -1.2232 | 0.2012 | 0.2519 |
| KEGG_INTESTINAL_IMMUNE_NETWORK_FOR_IGA_PRODUCTION | 46 | -0.4808 | -1.1867 | 0.3299 | 0.2837 |
| KEGG_GLYCOSPHINGOLIPID_BIOSYNTHESIS_GANGLIO_SERIES | 15 | -0.4427 | -1.1866 | 0.2677 | 0.2812 |
| KEGG_SELENOAMINO_ACID_METABOLISM | 26 | -0.4067 | -1.1854 | 0.2692 | 0.2798 |
| KEGG_OTHER_GLYCAN_DEGRADATION | 16 | -0.4460 | -1.1686 | 0.2631 | 0.2937 |
| KEGG_TYPE_I_DIABETES_MELLITUS | 41 | -0.4833 | -1.1627 | 0.3621 | 0.2975 |
| KEGG_PARKINSONS_DISEASE | 128 | -0.3829 | -1.1552 | 0.2963 | 0.3021 |
| KEGG_PENTOSE_PHOSPHATE_PATHWAY | 27 | -0.3825 | -1.1484 | 0.2740 | 0.3057 |
| KEGG_HEMATOPOIETIC_CELL_LINEAGE | 85 | -0.3932 | -1.1442 | 0.3286 | 0.3073 |
| KEGG_OXIDATIVE_PHOSPHORYLATION | 131 | -0.3788 | -1.1145 | 0.3731 | 0.3349 |
| KEGG_ALDOSTERONE_REGULATED_SODIUM_REABSORPTION | 42 | -0.3457 | -1.0924 | 0.3360 | 0.3560 |
| KEGG_GLYCOSAMINOGLYCAN_DEGRADATION | 21 | -0.3783 | -1.0759 | 0.3735 | 0.3707 |
| KEGG_PRIMARY_IMMUNODEFICIENCY | 35 | -0.4718 | -1.0713 | 0.4570 | 0.3723 |
| KEGG_SYSTEMIC_LUPUS_ERYTHEMATOSUS | 135 | -0.3243 | -1.0706 | 0.3912 | 0.3700 |
| KEGG_ASTHMA | 28 | -0.4209 | -1.0660 | 0.3937 | 0.3718 |
| KEGG_O_GLYCAN_BIOSYNTHESIS | 30 | -0.3847 | -1.0512 | 0.4212 | 0.3857 |
| KEGG_NEUROACTIVE_LIGAND_RECEPTOR_INTERACTION | 271 | -0.2758 | -1.0294 | 0.3919 | 0.4061 |
| KEGG_MATURITY_ONSET_DIABETES_OF_THE_YOUNG | 25 | -0.3389 | -0.9703 | 0.4960 | 0.4698 |
| KEGG_OLFACTORY_TRANSDUCTION | 383 | -0.1920 | -0.8937 | 0.6883 | 0.5624 |
| KEGG_GRAFT_VERSUS_HOST_DISEASE | 37 | -0.4004 | -0.8778 | 0.6091 | 0.5792 |
| KEGG_ALLOGRAFT_REJECTION | 35 | -0.3962 | -0.8769 | 0.5960 | 0.5758 |
| KEGG_AUTOIMMUNE_THYROID_DISEASE | 50 | -0.3235 | -0.8252 | 0.6263 | 0.6415 |
| KEGG_ALPHA_LINOLENIC_ACID_METABOLISM | 19 | -0.2422 | -0.7124 | 0.8723 | 0.7883 |

| **Signaling pathways enriched in HCC samples corresponding ADH1C expression by GSEA based on KEGG** | | | | | |
| --- | --- | --- | --- | --- | --- |
| **KEGG Name** | **Size** | **ES** | **NES** | **NOM p-value** | **FDR q-value** |
| KEGG_DRUG_METABOLISM_CYTOCHROME_P450 | 71 | 0.7992 | 2.4047 | 0.0000 | 0.0000 |
| KEGG_RETINOL_METABOLISM | 64 | 0.8016 | 2.3849 | 0.0000 | 0.0000 |
| KEGG_METABOLISM_OF_XENOBIOTICS_BY_CYTOCHROME_P450 | 69 | 0.7593 | 2.3258 | 0.0000 | 0.0000 |
| KEGG_COMPLEMENT_AND_COAGULATION_CASCADES | 69 | 0.7660 | 2.1805 | 0.0000 | 0.0000 |
| KEGG_FATTY_ACID_METABOLISM | 42 | 0.8858 | 2.1798 | 0.0000 | 0.0000 |
| KEGG_STEROID_HORMONE_BIOSYNTHESIS | 55 | 0.7100 | 2.1613 | 0.0000 | 0.0000 |
| KEGG_PRIMARY_BILE_ACID_BIOSYNTHESIS | 16 | 0.9257 | 2.1080 | 0.0000 | 0.0001 |
| KEGG_TRYPTOPHAN_METABOLISM | 40 | 0.7346 | 2.0794 | 0.0000 | 0.0002 |
| KEGG_ASCORBATE_AND_ALDARATE_METABOLISM | 25 | 0.8443 | 2.0782 | 0.0000 | 0.0002 |
| KEGG_BUTANOATE_METABOLISM | 34 | 0.7633 | 2.0733 | 0.0000 | 0.0002 |
| KEGG_PEROXISOME | 78 | 0.6744 | 2.0548 | 0.0000 | 0.0002 |
| KEGG_VALINE_LEUCINE_AND_ISOLEUCINE_DEGRADATION | 43 | 0.8359 | 2.0522 | 0.0000 | 0.0003 |
| KEGG_TYROSINE_METABOLISM | 42 | 0.6294 | 2.0317 | 0.0000 | 0.0005 |
| KEGG_PENTOSE_AND_GLUCURONATE_INTERCONVERSIONS | 28 | 0.7553 | 2.0036 | 0.0000 | 0.0007 |
| KEGG_BETA_ALANINE_METABOLISM | 22 | 0.7628 | 1.9813 | 0.0000 | 0.0009 |
| KEGG_PROPANOATE_METABOLISM | 32 | 0.7538 | 1.9047 | 0.0000 | 0.0025 |
| KEGG_DRUG_METABOLISM_OTHER_ENZYMES | 51 | 0.5951 | 1.8972 | 0.0000 | 0.0027 |
| KEGG_STARCH_AND_SUCROSE_METABOLISM | 52 | 0.5893 | 1.8901 | 0.0000 | 0.0029 |
| KEGG_PPAR_SIGNALING_PATHWAY | 69 | 0.5839 | 1.8564 | 0.0040 | 0.0044 |
| KEGG_GLYCINE_SERINE_AND_THREONINE_METABOLISM | 31 | 0.7091 | 1.8067 | 0.0064 | 0.0085 |
| KEGG_PORPHYRIN_AND_CHLOROPHYLL_METABOLISM | 41 | 0.5725 | 1.6931 | 0.0180 | 0.0252 |
| KEGG_PYRUVATE_METABOLISM | 40 | 0.5299 | 1.6564 | 0.0236 | 0.0333 |
| KEGG_PHENYLALANINE_METABOLISM | 18 | 0.5926 | 1.5800 | 0.0475 | 0.0577 |
| KEGG_HISTIDINE_METABOLISM | 29 | 0.5226 | 1.5759 | 0.0299 | 0.0570 |
| KEGG_GLYCOLYSIS_GLUCONEOGENESIS | 62 | 0.4632 | 1.5532 | 0.0426 | 0.0634 |
| KEGG_ARGININE_AND_PROLINE_METABOLISM | 54 | 0.4622 | 1.5369 | 0.0285 | 0.0672 |
| KEGG_LINOLEIC_ACID_METABOLISM | 29 | 0.4918 | 1.5253 | 0.0421 | 0.0696 |
| KEGG_ARACHIDONIC_ACID_METABOLISM | 58 | 0.3859 | 1.3696 | 0.0768 | 0.1591 |
| KEGG_CITRATE_CYCLE_TCA_CYCLE | 31 | 0.4986 | 1.3021 | 0.2086 | 0.2078 |
| KEGG_GLYOXYLATE_AND_DICARBOXYLATE_METABOLISM | 16 | 0.4573 | 1.2771 | 0.1751 | 0.2233 |
| KEGG_LYSINE_DEGRADATION | 44 | 0.4089 | 1.2501 | 0.2218 | 0.2403 |
| KEGG_ALANINE_ASPARTATE_AND_GLUTAMATE_METABOLISM | 32 | 0.3912 | 1.2388 | 0.2008 | 0.2443 |
| KEGG_ABC_TRANSPORTERS | 44 | 0.3757 | 1.2070 | 0.1976 | 0.2678 |
| KEGG_BIOSYNTHESIS_OF_UNSATURATED_FATTY_ACIDS | 22 | 0.4428 | 1.1850 | 0.2682 | 0.2818 |
| KEGG_GLYCEROLIPID_METABOLISM | 49 | 0.2869 | 1.0254 | 0.4050 | 0.4514 |
| KEGG_NICOTINATE_AND_NICOTINAMIDE_METABOLISM | 24 | 0.3159 | 0.9646 | 0.5120 | 0.5164 |
| KEGG_NITROGEN_METABOLISM | 23 | 0.3009 | 0.8953 | 0.5996 | 0.5951 |
| KEGG_RENIN_ANGIOTENSIN_SYSTEM | 17 | 0.3434 | 0.8791 | 0.6351 | 0.6015 |
| KEGG_TERPENOID_BACKBONE_BIOSYNTHESIS | 15 | 0.3092 | 0.7613 | 0.7267 | 0.7500 |
| KEGG_MATURITY_ONSET_DIABETES_OF_THE_YOUNG | 25 | 0.2059 | 0.5828 | 0.9901 | 0.9348 |
| KEGG_SPLICEOSOME | 127 | -0.7517 | -2.0067 | 0.0000 | 0.0397 |
| KEGG_THYROID_CANCER | 29 | -0.6960 | -1.9949 | 0.0000 | 0.0227 |
| KEGG_PROGESTERONE_MEDIATED_OOCYTE_MATURATION | 85 | -0.6169 | -1.9463 | 0.0000 | 0.0380 |
| KEGG_OOCYTE_MEIOSIS | 112 | -0.6076 | -1.9390 | 0.0000 | 0.0310 |
| KEGG_RNA_DEGRADATION | 59 | -0.6743 | -1.9368 | 0.0000 | 0.0257 |
| KEGG_BLADDER_CANCER | 42 | -0.6420 | -1.9193 | 0.0000 | 0.0271 |
| KEGG_PATHOGENIC_ESCHERICHIA_COLI_INFECTION | 56 | -0.6529 | -1.8789 | 0.0000 | 0.0388 |
| KEGG_CELL_CYCLE | 124 | -0.6727 | -1.8754 | 0.0020 | 0.0345 |
| KEGG_ENDOCYTOSIS | 181 | -0.5598 | -1.8596 | 0.0000 | 0.0352 |
| KEGG_PYRIMIDINE_METABOLISM | 98 | -0.5669 | -1.8560 | 0.0020 | 0.0334 |
| KEGG_FC_GAMMA_R_MEDIATED_PHAGOCYTOSIS | 96 | -0.6201 | -1.8487 | 0.0000 | 0.0327 |
| KEGG_VEGF_SIGNALING_PATHWAY | 76 | -0.5490 | -1.8478 | 0.0022 | 0.0302 |
| KEGG_HOMOLOGOUS_RECOMBINATION | 28 | -0.7407 | -1.8356 | 0.0000 | 0.0314 |
| KEGG_PURINE_METABOLISM | 158 | -0.5112 | -1.8303 | 0.0000 | 0.0306 |
| KEGG_BASE_EXCISION_REPAIR | 35 | -0.6846 | -1.8219 | 0.0021 | 0.0314 |
| KEGG_GNRH_SIGNALING_PATHWAY | 101 | -0.5381 | -1.8117 | 0.0000 | 0.0326 |
| KEGG_MTOR_SIGNALING_PATHWAY | 52 | -0.5861 | -1.8077 | 0.0021 | 0.0315 |
| KEGG_NUCLEOTIDE_EXCISION_REPAIR | 44 | -0.6690 | -1.7953 | 0.0000 | 0.0346 |
| KEGG_UBIQUITIN_MEDIATED_PROTEOLYSIS | 134 | -0.5866 | -1.7905 | 0.0019 | 0.0351 |
| KEGG_FC_EPSILON_RI_SIGNALING_PATHWAY | 79 | -0.5341 | -1.7809 | 0.0143 | 0.0384 |
| KEGG_RENAL_CELL_CARCINOMA | 70 | -0.5904 | -1.7795 | 0.0021 | 0.0369 |
| KEGG_DNA_REPLICATION | 36 | -0.7770 | -1.7782 | 0.0101 | 0.0353 |
| KEGG_PATHWAYS_IN_CANCER | 325 | -0.5214 | -1.7775 | 0.0021 | 0.0340 |
| KEGG_ADHERENS_JUNCTION | 73 | -0.5920 | -1.7647 | 0.0081 | 0.0370 |
| KEGG_RIBOFLAVIN_METABOLISM | 16 | -0.6609 | -1.7622 | 0.0040 | 0.0361 |
| KEGG_GLYCEROPHOSPHOLIPID_METABOLISM | 77 | -0.4845 | -1.7621 | 0.0021 | 0.0347 |
| KEGG_NOTCH_SIGNALING_PATHWAY | 47 | -0.5992 | -1.7586 | 0.0062 | 0.0350 |
| KEGG_VASOPRESSIN_REGULATED_WATER_REABSORPTION | 44 | -0.6180 | -1.7580 | 0.0042 | 0.0341 |
| KEGG_WNT_SIGNALING_PATHWAY | 150 | -0.5310 | -1.7577 | 0.0042 | 0.0330 |
| KEGG_COLORECTAL_CANCER | 62 | -0.5913 | -1.7567 | 0.0021 | 0.0321 |
| KEGG_ACUTE_MYELOID_LEUKEMIA | 57 | -0.5714 | -1.7565 | 0.0040 | 0.0311 |
| KEGG_AXON_GUIDANCE | 129 | -0.5430 | -1.7448 | 0.0105 | 0.0338 |
| KEGG_BASAL_TRANSCRIPTION_FACTORS | 35 | -0.6347 | -1.7381 | 0.0020 | 0.0348 |
| KEGG_INSULIN_SIGNALING_PATHWAY | 137 | -0.4861 | -1.7308 | 0.0020 | 0.0363 |
| KEGG_REGULATION_OF_ACTIN_CYTOSKELETON | 213 | -0.5182 | -1.7205 | 0.0064 | 0.0390 |
| KEGG_P53_SIGNALING_PATHWAY | 68 | -0.5224 | -1.7200 | 0.0040 | 0.0381 |
| KEGG_NOD_LIKE_RECEPTOR_SIGNALING_PATHWAY | 62 | -0.5708 | -1.7173 | 0.0143 | 0.0382 |
| KEGG_T_CELL_RECEPTOR_SIGNALING_PATHWAY | 108 | -0.5701 | -1.7126 | 0.0267 | 0.0391 |
| KEGG_PANCREATIC_CANCER | 70 | -0.5837 | -1.7055 | 0.0042 | 0.0413 |
| KEGG_INOSITOL_PHOSPHATE_METABOLISM | 54 | -0.5731 | -1.7029 | 0.0103 | 0.0414 |
| KEGG_NEUROTROPHIN_SIGNALING_PATHWAY | 126 | -0.5394 | -1.6931 | 0.0082 | 0.0448 |
| KEGG_GLYCOSAMINOGLYCAN_BIOSYNTHESIS_CHONDROITIN_SULFATE | 22 | -0.6347 | -1.6924 | 0.0183 | 0.0439 |
| KEGG_RNA_POLYMERASE | 29 | -0.6375 | -1.6869 | 0.0059 | 0.0453 |
| KEGG_PHOSPHATIDYLINOSITOL_SIGNALING_SYSTEM | 76 | -0.5512 | -1.6822 | 0.0146 | 0.0462 |
| KEGG_CHRONIC_MYELOID_LEUKEMIA | 73 | -0.5598 | -1.6775 | 0.0062 | 0.0470 |
| KEGG_ERBB_SIGNALING_PATHWAY | 87 | -0.5233 | -1.6753 | 0.0082 | 0.0472 |
| KEGG_HUNTINGTONS_DISEASE | 180 | -0.5003 | -1.6679 | 0.0287 | 0.0495 |
| KEGG_ETHER_LIPID_METABOLISM | 33 | -0.5212 | -1.6671 | 0.0105 | 0.0487 |
| KEGG_SMALL_CELL_LUNG_CANCER | 84 | -0.5423 | -1.6634 | 0.0165 | 0.0494 |
| KEGG_NON_SMALL_CELL_LUNG_CANCER | 54 | -0.5475 | -1.6586 | 0.0062 | 0.0504 |
| KEGG_LONG_TERM_DEPRESSION | 70 | -0.4979 | -1.6554 | 0.0129 | 0.0505 |
| KEGG_MELANOGENESIS | 101 | -0.4873 | -1.6496 | 0.0063 | 0.0527 |
| KEGG_SNARE_INTERACTIONS_IN_VESICULAR_TRANSPORT | 38 | -0.5523 | -1.6456 | 0.0216 | 0.0534 |
| KEGG_GLIOMA | 65 | -0.5079 | -1.6356 | 0.0120 | 0.0573 |
| KEGG_AMINOACYL_TRNA_BIOSYNTHESIS | 41 | -0.6184 | -1.6337 | 0.0278 | 0.0570 |
| KEGG_REGULATION_OF_AUTOPHAGY | 35 | -0.5245 | -1.6326 | 0.0179 | 0.0565 |
| KEGG_VIBRIO_CHOLERAE_INFECTION | 54 | -0.5201 | -1.6324 | 0.0248 | 0.0555 |
| KEGG_TIGHT_JUNCTION | 132 | -0.4745 | -1.6320 | 0.0123 | 0.0547 |
| KEGG_LYSOSOME | 121 | -0.4993 | -1.6295 | 0.0325 | 0.0548 |
| KEGG_MAPK_SIGNALING_PATHWAY | 267 | -0.4611 | -1.6243 | 0.0105 | 0.0559 |
| KEGG_EPITHELIAL_CELL_SIGNALING_IN_HELICOBACTER_PYLORI_INFECTION | 68 | -0.5185 | -1.6236 | 0.0268 | 0.0553 |
| KEGG_MISMATCH_REPAIR | 23 | -0.6965 | -1.6217 | 0.0348 | 0.0552 |
| KEGG_N_GLYCAN_BIOSYNTHESIS | 46 | -0.5417 | -1.6130 | 0.0235 | 0.0576 |
| KEGG_PROSTATE_CANCER | 89 | -0.5012 | -1.6117 | 0.0244 | 0.0572 |
| KEGG_LONG_TERM_POTENTIATION | 70 | -0.4891 | -1.6110 | 0.0120 | 0.0566 |
| KEGG_GLYCOSAMINOGLYCAN_BIOSYNTHESIS_HEPARAN_SULFATE | 26 | -0.5650 | -1.6085 | 0.0241 | 0.0565 |
| KEGG_OTHER_GLYCAN_DEGRADATION | 16 | -0.6180 | -1.6085 | 0.0275 | 0.0556 |
| KEGG_APOPTOSIS | 87 | -0.5036 | -1.6051 | 0.0277 | 0.0561 |
| KEGG_PROTEIN_EXPORT | 24 | -0.5986 | -1.6017 | 0.0354 | 0.0565 |
| KEGG_SELENOAMINO_ACID_METABOLISM | 26 | -0.5319 | -1.5965 | 0.0193 | 0.0578 |
| KEGG_TGF_BETA_SIGNALING_PATHWAY | 85 | -0.5092 | -1.5952 | 0.0327 | 0.0573 |
| KEGG_GAP_JUNCTION | 90 | -0.5048 | -1.5765 | 0.0381 | 0.0647 |
| KEGG_GLYCOSYLPHOSPHATIDYLINOSITOL_GPI_ANCHOR_BIOSYNTHESIS | 25 | -0.5698 | -1.5583 | 0.0584 | 0.0724 |
| KEGG_FRUCTOSE_AND_MANNOSE_METABOLISM | 33 | -0.4792 | -1.5578 | 0.0271 | 0.0716 |
| KEGG_GLYCOSPHINGOLIPID_BIOSYNTHESIS_GANGLIO_SERIES | 15 | -0.5689 | -1.5569 | 0.0475 | 0.0710 |
| KEGG_B_CELL_RECEPTOR_SIGNALING_PATHWAY | 75 | -0.5171 | -1.5527 | 0.0806 | 0.0720 |
| KEGG_ALZHEIMERS_DISEASE | 165 | -0.4521 | -1.5511 | 0.0554 | 0.0719 |
| KEGG_MELANOMA | 71 | -0.4711 | -1.5465 | 0.0234 | 0.0732 |
| KEGG_DORSO_VENTRAL_AXIS_FORMATION | 24 | -0.5655 | -1.5452 | 0.0560 | 0.0732 |
| KEGG_CYTOSOLIC_DNA_SENSING_PATHWAY | 55 | -0.4995 | -1.5309 | 0.0494 | 0.0793 |
| KEGG_LEUKOCYTE_TRANSENDOTHELIAL_MIGRATION | 116 | -0.4675 | -1.5278 | 0.0565 | 0.0798 |
| KEGG_NATURAL_KILLER_CELL_MEDIATED_CYTOTOXICITY | 132 | -0.4833 | -1.5229 | 0.0789 | 0.0810 |
| KEGG_ENDOMETRIAL_CANCER | 52 | -0.5078 | -1.5185 | 0.0553 | 0.0821 |
| KEGG_CHEMOKINE_SIGNALING_PATHWAY | 188 | -0.4670 | -1.5041 | 0.0710 | 0.0893 |
| KEGG_LEISHMANIA_INFECTION | 70 | -0.5349 | -1.4996 | 0.1020 | 0.0909 |
| KEGG_FOCAL_ADHESION | 199 | -0.4625 | -1.4910 | 0.0878 | 0.0947 |
| KEGG_TOLL_LIKE_RECEPTOR_SIGNALING_PATHWAY | 102 | -0.4537 | -1.4773 | 0.0732 | 0.1017 |
| KEGG_SPHINGOLIPID_METABOLISM | 39 | -0.4809 | -1.4739 | 0.0568 | 0.1023 |
| KEGG_TYPE_II_DIABETES_MELLITUS | 47 | -0.4592 | -1.4589 | 0.0523 | 0.1107 |
| KEGG_TASTE_TRANSDUCTION | 51 | -0.4538 | -1.4461 | 0.0473 | 0.1182 |
| KEGG_GLYCOSAMINOGLYCAN_BIOSYNTHESIS_KERATAN_SULFATE | 15 | -0.5665 | -1.4409 | 0.0847 | 0.1200 |
| KEGG_RIG_I_LIKE_RECEPTOR_SIGNALING_PATHWAY | 71 | -0.4477 | -1.4393 | 0.0768 | 0.1195 |
| KEGG_RIBOSOME | 87 | -0.7086 | -1.4318 | 0.1475 | 0.1233 |
| KEGG_VASCULAR_SMOOTH_MUSCLE_CONTRACTION | 115 | -0.4119 | -1.4109 | 0.1042 | 0.1366 |
| KEGG_GLYCOSPHINGOLIPID_BIOSYNTHESIS_LACTO_AND_NEOLACTO_SERIES | 26 | -0.4662 | -1.3896 | 0.1046 | 0.1505 |
| KEGG_JAK_STAT_SIGNALING_PATHWAY | 155 | -0.4066 | -1.3797 | 0.1204 | 0.1561 |
| KEGG_AMYOTROPHIC_LATERAL_SCLEROSIS_ALS | 53 | -0.3963 | -1.3650 | 0.0861 | 0.1652 |
| KEGG_HEDGEHOG_SIGNALING_PATHWAY | 56 | -0.4416 | -1.3615 | 0.1089 | 0.1664 |
| KEGG_HYPERTROPHIC_CARDIOMYOPATHY_HCM | 83 | -0.4198 | -1.3562 | 0.1263 | 0.1692 |
| KEGG_AMINO_SUGAR_AND_NUCLEOTIDE_SUGAR_METABOLISM | 43 | -0.4137 | -1.3469 | 0.1070 | 0.1744 |
| KEGG_ARRHYTHMOGENIC_RIGHT_VENTRICULAR_CARDIOMYOPATHY_ARVC | 74 | -0.4208 | -1.3201 | 0.1587 | 0.1941 |
| KEGG_PROTEASOME | 46 | -0.5049 | -1.3167 | 0.2143 | 0.1954 |
| KEGG_CARDIAC_MUSCLE_CONTRACTION | 78 | -0.4004 | -1.3050 | 0.1521 | 0.2038 |
| KEGG_CYSTEINE_AND_METHIONINE_METABOLISM | 34 | -0.4102 | -1.3043 | 0.1513 | 0.2025 |
| KEGG_BASAL_CELL_CARCINOMA | 55 | -0.4158 | -1.2983 | 0.1435 | 0.2055 |
| KEGG_CYTOKINE_CYTOKINE_RECEPTOR_INTERACTION | 264 | -0.3803 | -1.2946 | 0.1933 | 0.2070 |
| KEGG_ANTIGEN_PROCESSING_AND_PRESENTATION | 81 | -0.4706 | -1.2844 | 0.2273 | 0.2136 |
| KEGG_CALCIUM_SIGNALING_PATHWAY | 177 | -0.3546 | -1.2658 | 0.1519 | 0.2264 |
| KEGG_DILATED_CARDIOMYOPATHY | 90 | -0.4049 | -1.2623 | 0.1846 | 0.2272 |
| KEGG_GLYCOSAMINOGLYCAN_DEGRADATION | 21 | -0.4542 | -1.2503 | 0.1903 | 0.2357 |
| KEGG_HEMATOPOIETIC_CELL_LINEAGE | 85 | -0.4174 | -1.2469 | 0.2364 | 0.2364 |
| KEGG_PARKINSONS_DISEASE | 128 | -0.4058 | -1.2288 | 0.2590 | 0.2508 |
| KEGG_ASTHMA | 28 | -0.4912 | -1.2256 | 0.2814 | 0.2517 |
| KEGG_OXIDATIVE_PHOSPHORYLATION | 131 | -0.4076 | -1.1947 | 0.2990 | 0.2790 |
| KEGG_TYPE_I_DIABETES_MELLITUS | 41 | -0.4895 | -1.1874 | 0.3112 | 0.2837 |
| KEGG_INTESTINAL_IMMUNE_NETWORK_FOR_IGA_PRODUCTION | 46 | -0.4624 | -1.1736 | 0.3176 | 0.2949 |
| KEGG_VIRAL_MYOCARDITIS | 68 | -0.3993 | -1.1664 | 0.3160 | 0.2997 |
| KEGG_CELL_ADHESION_MOLECULES_CAMS | 131 | -0.3707 | -1.1598 | 0.3013 | 0.3036 |
| KEGG_PRIMARY_IMMUNODEFICIENCY | 35 | -0.4946 | -1.1527 | 0.3657 | 0.3077 |
| KEGG_PRION_DISEASES | 35 | -0.3542 | -1.0987 | 0.3407 | 0.3625 |
| KEGG_NEUROACTIVE_LIGAND_RECEPTOR_INTERACTION | 271 | -0.2847 | -1.0890 | 0.2914 | 0.3700 |
| KEGG_PENTOSE_PHOSPHATE_PATHWAY | 27 | -0.3675 | -1.0875 | 0.3464 | 0.3686 |
| KEGG_ONE_CARBON_POOL_BY_FOLATE | 17 | -0.3774 | -1.0836 | 0.3659 | 0.3701 |
| KEGG_ECM_RECEPTOR_INTERACTION | 84 | -0.3443 | -1.0673 | 0.3825 | 0.3864 |
| KEGG_ADIPOCYTOKINE_SIGNALING_PATHWAY | 67 | -0.2920 | -1.0331 | 0.3937 | 0.4224 |
| KEGG_ALPHA_LINOLENIC_ACID_METABOLISM | 19 | -0.3422 | -1.0144 | 0.4247 | 0.4417 |
| KEGG_ALLOGRAFT_REJECTION | 35 | -0.4456 | -0.9897 | 0.5049 | 0.4674 |
| KEGG_O_GLYCAN_BIOSYNTHESIS | 30 | -0.3568 | -0.9881 | 0.4853 | 0.4656 |
| KEGG_GRAFT_VERSUS_HOST_DISEASE | 37 | -0.4398 | -0.9694 | 0.5359 | 0.4844 |
| KEGG_GALACTOSE_METABOLISM | 26 | -0.3065 | -0.9590 | 0.4857 | 0.4939 |
| KEGG_GLUTATHIONE_METABOLISM | 49 | -0.2935 | -0.9580 | 0.5240 | 0.4915 |
| KEGG_PROXIMAL_TUBULE_BICARBONATE_RECLAMATION | 23 | -0.3216 | -0.9566 | 0.5000 | 0.4898 |
| KEGG_ALDOSTERONE_REGULATED_SODIUM_REABSORPTION | 42 | -0.2930 | -0.9390 | 0.5678 | 0.5078 |
| KEGG_PANTOTHENATE_AND_COA_BIOSYNTHESIS | 16 | -0.3340 | -0.9173 | 0.5551 | 0.5312 |
| KEGG_SYSTEMIC_LUPUS_ERYTHEMATOSUS | 135 | -0.2691 | -0.8718 | 0.6139 | 0.5875 |
| KEGG_AUTOIMMUNE_THYROID_DISEASE | 50 | -0.3283 | -0.8474 | 0.6209 | 0.6153 |
| KEGG_OLFACTORY_TRANSDUCTION | 383 | -0.1809 | -0.8438 | 0.8412 | 0.6158 |
| KEGG_STEROID_BIOSYNTHESIS | 17 | -0.3411 | -0.7924 | 0.6908 | 0.6824 |

| **Signaling pathways enriched in HCC samples corresponding ADH4 expression by GSEA based on KEGG** | | | | | |
| --- | --- | --- | --- | --- | --- |
| **KEGG Name** | **Size** | **ES** | **NES** | **NOM p-value** | **FDR q-value** |
| KEGG_RETINOL_METABOLISM | 64 | 0.7690 | 2.2631 | 0.0000 | 0.0000 |
| KEGG_DRUG_METABOLISM_CYTOCHROME_P450 | 71 | 0.7660 | 2.2258 | 0.0000 | 0.0005 |
| KEGG_FATTY_ACID_METABOLISM | 42 | 0.8849 | 2.1621 | 0.0000 | 0.0009 |
| KEGG_TYROSINE_METABOLISM | 42 | 0.6809 | 2.1590 | 0.0000 | 0.0007 |
| KEGG_GLYCINE_SERINE_AND_THREONINE_METABOLISM | 31 | 0.8366 | 2.1275 | 0.0000 | 0.0013 |
| KEGG_METABOLISM_OF_XENOBIOTICS_BY_CYTOCHROME_P450 | 69 | 0.6965 | 2.1164 | 0.0020 | 0.0011 |
| KEGG_PEROXISOME | 78 | 0.6940 | 2.0863 | 0.0020 | 0.0012 |
| KEGG_VALINE_LEUCINE_AND_ISOLEUCINE_DEGRADATION | 43 | 0.8460 | 2.0594 | 0.0000 | 0.0013 |
| KEGG_TRYPTOPHAN_METABOLISM | 40 | 0.7268 | 2.0542 | 0.0000 | 0.0012 |
| KEGG_COMPLEMENT_AND_COAGULATION_CASCADES | 69 | 0.7242 | 2.0472 | 0.0020 | 0.0010 |
| KEGG_PPAR_SIGNALING_PATHWAY | 69 | 0.6394 | 2.0438 | 0.0000 | 0.0010 |
| KEGG_PRIMARY_BILE_ACID_BIOSYNTHESIS | 16 | 0.9241 | 2.0317 | 0.0000 | 0.0010 |
| KEGG_BUTANOATE_METABOLISM | 34 | 0.7471 | 1.9688 | 0.0000 | 0.0020 |
| KEGG_PROPANOATE_METABOLISM | 32 | 0.7611 | 1.9600 | 0.0000 | 0.0021 |
| KEGG_BETA_ALANINE_METABOLISM | 22 | 0.7693 | 1.9597 | 0.0000 | 0.0020 |
| KEGG_ARGININE_AND_PROLINE_METABOLISM | 54 | 0.5925 | 1.9306 | 0.0019 | 0.0029 |
| KEGG_STEROID_HORMONE_BIOSYNTHESIS | 55 | 0.6184 | 1.9047 | 0.0060 | 0.0038 |
| KEGG_HISTIDINE_METABOLISM | 29 | 0.6152 | 1.8225 | 0.0000 | 0.0088 |
| KEGG_ASCORBATE_AND_ALDARATE_METABOLISM | 25 | 0.6945 | 1.7420 | 0.0281 | 0.0205 |
| KEGG_ALANINE_ASPARTATE_AND_GLUTAMATE_METABOLISM | 32 | 0.5443 | 1.7129 | 0.0150 | 0.0248 |
| KEGG_PYRUVATE_METABOLISM | 40 | 0.5481 | 1.6954 | 0.0152 | 0.0280 |
| KEGG_GLYCOLYSIS_GLUCONEOGENESIS | 62 | 0.4879 | 1.6853 | 0.0074 | 0.0294 |
| KEGG_PHENYLALANINE_METABOLISM | 18 | 0.6057 | 1.6849 | 0.0173 | 0.0281 |
| KEGG_DRUG_METABOLISM_OTHER_ENZYMES | 51 | 0.5170 | 1.6168 | 0.0375 | 0.0482 |
| KEGG_NITROGEN_METABOLISM | 23 | 0.5527 | 1.6060 | 0.0217 | 0.0507 |
| KEGG_PENTOSE_AND_GLUCURONATE_INTERCONVERSIONS | 28 | 0.5753 | 1.5553 | 0.0575 | 0.0703 |
| KEGG_GLYOXYLATE_AND_DICARBOXYLATE_METABOLISM | 16 | 0.5615 | 1.5472 | 0.0470 | 0.0716 |
| KEGG_LINOLEIC_ACID_METABOLISM | 29 | 0.4897 | 1.4909 | 0.0466 | 0.0983 |
| KEGG_LYSINE_DEGRADATION | 44 | 0.4872 | 1.4423 | 0.0902 | 0.1241 |
| KEGG_STARCH_AND_SUCROSE_METABOLISM | 52 | 0.4526 | 1.4312 | 0.0830 | 0.1277 |
| KEGG_CITRATE_CYCLE_TCA_CYCLE | 31 | 0.5430 | 1.4270 | 0.1319 | 0.1260 |
| KEGG_ONE_CARBON_POOL_BY_FOLATE | 17 | 0.4861 | 1.4044 | 0.0904 | 0.1381 |
| KEGG_GLYCEROLIPID_METABOLISM | 49 | 0.3965 | 1.4033 | 0.0508 | 0.1346 |
| KEGG_BIOSYNTHESIS_OF_UNSATURATED_FATTY_ACIDS | 22 | 0.5163 | 1.3968 | 0.1148 | 0.1350 |
| KEGG_PORPHYRIN_AND_CHLOROPHYLL_METABOLISM | 41 | 0.4343 | 1.3113 | 0.1594 | 0.1928 |
| KEGG_CYSTEINE_AND_METHIONINE_METABOLISM | 34 | 0.4013 | 1.2676 | 0.1747 | 0.2238 |
| KEGG_ARACHIDONIC_ACID_METABOLISM | 58 | 0.3482 | 1.2447 | 0.1498 | 0.2382 |
| KEGG_ABC_TRANSPORTERS | 44 | 0.3545 | 1.1461 | 0.2452 | 0.3300 |
| KEGG_TERPENOID_BACKBONE_BIOSYNTHESIS | 15 | 0.4238 | 1.0357 | 0.4198 | 0.4489 |
| KEGG_PANTOTHENATE_AND_COA_BIOSYNTHESIS | 16 | 0.3606 | 0.9963 | 0.4618 | 0.4850 |
| KEGG_PROXIMAL_TUBULE_BICARBONATE_RECLAMATION | 23 | 0.3406 | 0.9918 | 0.4735 | 0.4789 |
| KEGG_ADIPOCYTOKINE_SIGNALING_PATHWAY | 67 | 0.2748 | 0.9583 | 0.5158 | 0.5083 |
| KEGG_RENIN_ANGIOTENSIN_SYSTEM | 17 | 0.3528 | 0.9146 | 0.5661 | 0.5499 |
| KEGG_NICOTINATE_AND_NICOTINAMIDE_METABOLISM | 24 | 0.2751 | 0.8333 | 0.7268 | 0.6445 |
| KEGG_STEROID_BIOSYNTHESIS | 17 | 0.3421 | 0.7960 | 0.6551 | 0.6784 |
| KEGG_ENDOCYTOSIS | 181 | -0.6046 | -2.0388 | 0.0000 | 0.0197 |
| KEGG_PATHOGENIC_ESCHERICHIA_COLI_INFECTION | 56 | -0.6860 | -2.0311 | 0.0000 | 0.0110 |
| KEGG_RENAL_CELL_CARCINOMA | 70 | -0.6566 | -2.0095 | 0.0000 | 0.0104 |
| KEGG_REGULATION_OF_ACTIN_CYTOSKELETON | 213 | -0.5833 | -1.9783 | 0.0000 | 0.0140 |
| KEGG_NOTCH_SIGNALING_PATHWAY | 47 | -0.6853 | -1.9640 | 0.0000 | 0.0133 |
| KEGG_PURINE_METABOLISM | 158 | -0.5265 | -1.9562 | 0.0000 | 0.0129 |
| KEGG_SPLICEOSOME | 127 | -0.7285 | -1.9508 | 0.0000 | 0.0122 |
| KEGG_BLADDER_CANCER | 42 | -0.6503 | -1.9492 | 0.0000 | 0.0109 |
| KEGG_CELL_CYCLE | 124 | -0.6559 | -1.9077 | 0.0000 | 0.0170 |
| KEGG_OOCYTE_MEIOSIS | 112 | -0.5771 | -1.9051 | 0.0000 | 0.0161 |
| KEGG_PATHWAYS_IN_CANCER | 325 | -0.5491 | -1.8934 | 0.0021 | 0.0176 |
| KEGG_CHRONIC_MYELOID_LEUKEMIA | 73 | -0.6234 | -1.8909 | 0.0000 | 0.0165 |
| KEGG_MELANOMA | 71 | -0.5543 | -1.8746 | 0.0000 | 0.0192 |
| KEGG_FC_GAMMA_R_MEDIATED_PHAGOCYTOSIS | 96 | -0.6205 | -1.8736 | 0.0042 | 0.0178 |
| KEGG_VEGF_SIGNALING_PATHWAY | 76 | -0.5463 | -1.8599 | 0.0021 | 0.0202 |
| KEGG_VASOPRESSIN_REGULATED_WATER_REABSORPTION | 44 | -0.6418 | -1.8580 | 0.0040 | 0.0194 |
| KEGG_MTOR_SIGNALING_PATHWAY | 52 | -0.5976 | -1.8561 | 0.0000 | 0.0189 |
| KEGG_VIBRIO_CHOLERAE_INFECTION | 54 | -0.5873 | -1.8508 | 0.0020 | 0.0190 |
| KEGG_PYRIMIDINE_METABOLISM | 98 | -0.5464 | -1.8492 | 0.0000 | 0.0186 |
| KEGG_PROGESTERONE_MEDIATED_OOCYTE_MATURATION | 85 | -0.5708 | -1.8428 | 0.0000 | 0.0192 |
| KEGG_LYSOSOME | 121 | -0.5649 | -1.8326 | 0.0106 | 0.0214 |
| KEGG_AMINOACYL_TRNA_BIOSYNTHESIS | 41 | -0.6783 | -1.8310 | 0.0020 | 0.0206 |
| KEGG_DNA_REPLICATION | 36 | -0.7666 | -1.8243 | 0.0021 | 0.0213 |
| KEGG_PANCREATIC_CANCER | 70 | -0.6103 | -1.8141 | 0.0000 | 0.0232 |
| KEGG_AXON_GUIDANCE | 129 | -0.5662 | -1.8060 | 0.0041 | 0.0243 |
| KEGG_PROSTATE_CANCER | 89 | -0.5528 | -1.8009 | 0.0000 | 0.0249 |
| KEGG_LONG_TERM_DEPRESSION | 70 | -0.5255 | -1.8001 | 0.0020 | 0.0242 |
| KEGG_EPITHELIAL_CELL_SIGNALING_IN_HELICOBACTER_PYLORI_INFECTION | 68 | -0.5649 | -1.7946 | 0.0061 | 0.0244 |
| KEGG_SNARE_INTERACTIONS_IN_VESICULAR_TRANSPORT | 38 | -0.6053 | -1.7932 | 0.0020 | 0.0239 |
| KEGG_NEUROTROPHIN_SIGNALING_PATHWAY | 126 | -0.5697 | -1.7892 | 0.0084 | 0.0238 |
| KEGG_MISMATCH_REPAIR | 23 | -0.7334 | -1.7840 | 0.0126 | 0.0242 |
| KEGG_HOMOLOGOUS_RECOMBINATION | 28 | -0.7000 | -1.7826 | 0.0063 | 0.0239 |
| KEGG_SMALL_CELL_LUNG_CANCER | 84 | -0.5853 | -1.7777 | 0.0000 | 0.0237 |
| KEGG_MAPK_SIGNALING_PATHWAY | 267 | -0.4916 | -1.7766 | 0.0000 | 0.0234 |
| KEGG_TOLL_LIKE_RECEPTOR_SIGNALING_PATHWAY | 102 | -0.5435 | -1.7703 | 0.0062 | 0.0250 |
| KEGG_P53_SIGNALING_PATHWAY | 68 | -0.5397 | -1.7658 | 0.0022 | 0.0254 |
| KEGG_NOD_LIKE_RECEPTOR_SIGNALING_PATHWAY | 62 | -0.5976 | -1.7578 | 0.0043 | 0.0267 |
| KEGG_GNRH_SIGNALING_PATHWAY | 101 | -0.5177 | -1.7572 | 0.0060 | 0.0262 |
| KEGG_PHOSPHATIDYLINOSITOL_SIGNALING_SYSTEM | 76 | -0.5692 | -1.7502 | 0.0041 | 0.0269 |
| KEGG_WNT_SIGNALING_PATHWAY | 150 | -0.5245 | -1.7476 | 0.0041 | 0.0271 |
| KEGG_HUNTINGTONS_DISEASE | 180 | -0.5117 | -1.7461 | 0.0251 | 0.0266 |
| KEGG_RNA_POLYMERASE | 29 | -0.6569 | -1.7420 | 0.0000 | 0.0271 |
| KEGG_NON_SMALL_CELL_LUNG_CANCER | 54 | -0.5597 | -1.7381 | 0.0062 | 0.0277 |
| KEGG_GLYCOSPHINGOLIPID_BIOSYNTHESIS_LACTO_AND_NEOLACTO_SERIES | 26 | -0.5894 | -1.7352 | 0.0020 | 0.0276 |
| KEGG_ERBB_SIGNALING_PATHWAY | 87 | -0.5452 | -1.7283 | 0.0021 | 0.0291 |
| KEGG_RNA_DEGRADATION | 59 | -0.6068 | -1.7265 | 0.0043 | 0.0289 |
| KEGG_TIGHT_JUNCTION | 132 | -0.4955 | -1.7248 | 0.0082 | 0.0287 |
| KEGG_INOSITOL_PHOSPHATE_METABOLISM | 54 | -0.5822 | -1.7246 | 0.0063 | 0.0282 |
| KEGG_GLYCEROPHOSPHOLIPID_METABOLISM | 77 | -0.4826 | -1.7231 | 0.0000 | 0.0279 |
| KEGG_GAP_JUNCTION | 90 | -0.5333 | -1.7167 | 0.0040 | 0.0294 |
| KEGG_FC_EPSILON_RI_SIGNALING_PATHWAY | 79 | -0.5181 | -1.7150 | 0.0104 | 0.0293 |
| KEGG_NUCLEOTIDE_EXCISION_REPAIR | 44 | -0.6093 | -1.7142 | 0.0142 | 0.0289 |
| KEGG_ETHER_LIPID_METABOLISM | 33 | -0.5371 | -1.7127 | 0.0020 | 0.0288 |
| KEGG_COLORECTAL_CANCER | 62 | -0.5719 | -1.7094 | 0.0042 | 0.0290 |
| KEGG_N_GLYCAN_BIOSYNTHESIS | 46 | -0.5782 | -1.7085 | 0.0084 | 0.0286 |
| KEGG_ADHERENS_JUNCTION | 73 | -0.5781 | -1.7073 | 0.0106 | 0.0283 |
| KEGG_LONG_TERM_POTENTIATION | 70 | -0.5076 | -1.6954 | 0.0101 | 0.0308 |
| KEGG_GLIOMA | 65 | -0.5201 | -1.6953 | 0.0124 | 0.0303 |
| KEGG_UBIQUITIN_MEDIATED_PROTEOLYSIS | 134 | -0.5544 | -1.6908 | 0.0081 | 0.0313 |
| KEGG_HYPERTROPHIC_CARDIOMYOPATHY_HCM | 83 | -0.5267 | -1.6875 | 0.0103 | 0.0316 |
| KEGG_CYTOSOLIC_DNA_SENSING_PATHWAY | 55 | -0.5533 | -1.6870 | 0.0147 | 0.0313 |
| KEGG_ACUTE_MYELOID_LEUKEMIA | 57 | -0.5458 | -1.6838 | 0.0083 | 0.0315 |
| KEGG_T_CELL_RECEPTOR_SIGNALING_PATHWAY | 108 | -0.5662 | -1.6773 | 0.0207 | 0.0331 |
| KEGG_RIBOFLAVIN_METABOLISM | 16 | -0.6200 | -1.6724 | 0.0104 | 0.0342 |
| KEGG_TGF_BETA_SIGNALING_PATHWAY | 85 | -0.5391 | -1.6721 | 0.0147 | 0.0337 |
| KEGG_BASE_EXCISION_REPAIR | 35 | -0.5983 | -1.6620 | 0.0162 | 0.0362 |
| KEGG_CHEMOKINE_SIGNALING_PATHWAY | 188 | -0.5162 | -1.6599 | 0.0187 | 0.0362 |
| KEGG_THYROID_CANCER | 29 | -0.5850 | -1.6552 | 0.0062 | 0.0371 |
| KEGG_FOCAL_ADHESION | 199 | -0.5120 | -1.6550 | 0.0247 | 0.0366 |
| KEGG_INSULIN_SIGNALING_PATHWAY | 137 | -0.4635 | -1.6522 | 0.0152 | 0.0372 |
| KEGG_B_CELL_RECEPTOR_SIGNALING_PATHWAY | 75 | -0.5578 | -1.6498 | 0.0281 | 0.0373 |
| KEGG_APOPTOSIS | 87 | -0.5242 | -1.6427 | 0.0105 | 0.0388 |
| KEGG_GLYCOSAMINOGLYCAN_BIOSYNTHESIS_CHONDROITIN_SULFATE | 22 | -0.6324 | -1.6414 | 0.0123 | 0.0387 |
| KEGG_MELANOGENESIS | 101 | -0.4742 | -1.6322 | 0.0144 | 0.0409 |
| KEGG_LEUKOCYTE_TRANSENDOTHELIAL_MIGRATION | 116 | -0.4991 | -1.6271 | 0.0271 | 0.0420 |
| KEGG_LEISHMANIA_INFECTION | 70 | -0.5845 | -1.6244 | 0.0473 | 0.0421 |
| KEGG_NATURAL_KILLER_CELL_MEDIATED_CYTOTOXICITY | 132 | -0.5117 | -1.6114 | 0.0266 | 0.0459 |
| KEGG_ALZHEIMERS_DISEASE | 165 | -0.4749 | -1.6092 | 0.0456 | 0.0461 |
| KEGG_GLYCOSAMINOGLYCAN_BIOSYNTHESIS_HEPARAN_SULFATE | 26 | -0.5700 | -1.6072 | 0.0219 | 0.0460 |
| KEGG_AMYOTROPHIC_LATERAL_SCLEROSIS_ALS | 53 | -0.4563 | -1.6064 | 0.0123 | 0.0456 |
| KEGG_DORSO_VENTRAL_AXIS_FORMATION | 24 | -0.5938 | -1.5825 | 0.0340 | 0.0532 |
| KEGG_HEDGEHOG_SIGNALING_PATHWAY | 56 | -0.5082 | -1.5803 | 0.0181 | 0.0533 |
| KEGG_BASAL_TRANSCRIPTION_FACTORS | 35 | -0.5673 | -1.5781 | 0.0317 | 0.0537 |
| KEGG_PROTEIN_EXPORT | 24 | -0.5908 | -1.5774 | 0.0365 | 0.0535 |
| KEGG_RIG_I_LIKE_RECEPTOR_SIGNALING_PATHWAY | 71 | -0.4984 | -1.5650 | 0.0280 | 0.0571 |
| KEGG_DILATED_CARDIOMYOPATHY | 90 | -0.4973 | -1.5538 | 0.0317 | 0.0610 |
| KEGG_CARDIAC_MUSCLE_CONTRACTION | 78 | -0.4725 | -1.5454 | 0.0335 | 0.0639 |
| KEGG_RIBOSOME | 87 | -0.7558 | -1.5413 | 0.0632 | 0.0650 |
| KEGG_ARRHYTHMOGENIC_RIGHT_VENTRICULAR_CARDIOMYOPATHY_ARVC | 74 | -0.4970 | -1.5393 | 0.0421 | 0.0651 |
| KEGG_CYTOKINE_CYTOKINE_RECEPTOR_INTERACTION | 264 | -0.4645 | -1.5326 | 0.0356 | 0.0677 |
| KEGG_JAK_STAT_SIGNALING_PATHWAY | 155 | -0.4614 | -1.5310 | 0.0418 | 0.0678 |
| KEGG_GLYCOSAMINOGLYCAN_BIOSYNTHESIS_KERATAN_SULFATE | 15 | -0.5918 | -1.5232 | 0.0573 | 0.0706 |
| KEGG_ECM_RECEPTOR_INTERACTION | 84 | -0.4858 | -1.4826 | 0.0868 | 0.0882 |
| KEGG_TASTE_TRANSDUCTION | 51 | -0.4577 | -1.4824 | 0.0376 | 0.0874 |
| KEGG_ENDOMETRIAL_CANCER | 52 | -0.4794 | -1.4568 | 0.0840 | 0.1000 |
| KEGG_SPHINGOLIPID_METABOLISM | 39 | -0.4849 | -1.4517 | 0.0602 | 0.1017 |
| KEGG_BASAL_CELL_CARCINOMA | 55 | -0.4679 | -1.4513 | 0.0688 | 0.1009 |
| KEGG_TYPE_II_DIABETES_MELLITUS | 47 | -0.4361 | -1.4205 | 0.0726 | 0.1190 |
| KEGG_PRION_DISEASES | 35 | -0.4490 | -1.4157 | 0.1032 | 0.1208 |
| KEGG_AMINO_SUGAR_AND_NUCLEOTIDE_SUGAR_METABOLISM | 43 | -0.4335 | -1.4075 | 0.0955 | 0.1248 |
| KEGG_GLYCOSYLPHOSPHATIDYLINOSITOL_GPI_ANCHOR_BIOSYNTHESIS | 25 | -0.5042 | -1.4003 | 0.1245 | 0.1281 |
| KEGG_ANTIGEN_PROCESSING_AND_PRESENTATION | 81 | -0.5129 | -1.3965 | 0.1503 | 0.1293 |
| KEGG_CELL_ADHESION_MOLECULES_CAMS | 131 | -0.4537 | -1.3965 | 0.1302 | 0.1280 |
| KEGG_VASCULAR_SMOOTH_MUSCLE_CONTRACTION | 115 | -0.4170 | -1.3946 | 0.0841 | 0.1281 |
| KEGG_CALCIUM_SIGNALING_PATHWAY | 177 | -0.3879 | -1.3853 | 0.0725 | 0.1327 |
| KEGG_HEMATOPOIETIC_CELL_LINEAGE | 85 | -0.4774 | -1.3724 | 0.1458 | 0.1397 |
| KEGG_FRUCTOSE_AND_MANNOSE_METABOLISM | 33 | -0.4182 | -1.3700 | 0.0860 | 0.1401 |
| KEGG_SELENOAMINO_ACID_METABOLISM | 26 | -0.4478 | -1.3407 | 0.1305 | 0.1589 |
| KEGG_VIRAL_MYOCARDITIS | 68 | -0.4582 | -1.3312 | 0.1828 | 0.1640 |
| KEGG_PROTEASOME | 46 | -0.5077 | -1.3169 | 0.2331 | 0.1725 |
| KEGG_PRIMARY_IMMUNODEFICIENCY | 35 | -0.5762 | -1.3136 | 0.2209 | 0.1734 |
| KEGG_INTESTINAL_IMMUNE_NETWORK_FOR_IGA_PRODUCTION | 46 | -0.5222 | -1.3020 | 0.2470 | 0.1808 |
| KEGG_OTHER_GLYCAN_DEGRADATION | 16 | -0.4906 | -1.2699 | 0.1889 | 0.2043 |
| KEGG_GLYCOSPHINGOLIPID_BIOSYNTHESIS_GANGLIO_SERIES | 15 | -0.4745 | -1.2427 | 0.2008 | 0.2253 |
| KEGG_REGULATION_OF_AUTOPHAGY | 35 | -0.3905 | -1.2291 | 0.2271 | 0.2345 |
| KEGG_TYPE_I_DIABETES_MELLITUS | 41 | -0.5022 | -1.2071 | 0.2932 | 0.2515 |
| KEGG_PENTOSE_PHOSPHATE_PATHWAY | 27 | -0.3929 | -1.1813 | 0.2320 | 0.2733 |
| KEGG_ALDOSTERONE_REGULATED_SODIUM_REABSORPTION | 42 | -0.3682 | -1.1789 | 0.2121 | 0.2731 |
| KEGG_O_GLYCAN_BIOSYNTHESIS | 30 | -0.4266 | -1.1732 | 0.2786 | 0.2756 |
| KEGG_GLUTATHIONE_METABOLISM | 49 | -0.3459 | -1.1582 | 0.2354 | 0.2873 |
| KEGG_NEUROACTIVE_LIGAND_RECEPTOR_INTERACTION | 271 | -0.2953 | -1.1224 | 0.2489 | 0.3198 |
| KEGG_PARKINSONS_DISEASE | 128 | -0.3806 | -1.1187 | 0.3541 | 0.3209 |
| KEGG_OXIDATIVE_PHOSPHORYLATION | 131 | -0.3961 | -1.1179 | 0.3518 | 0.3190 |
| KEGG_GLYCOSAMINOGLYCAN_DEGRADATION | 21 | -0.3789 | -1.0477 | 0.3991 | 0.3928 |
| KEGG_MATURITY_ONSET_DIABETES_OF_THE_YOUNG | 25 | -0.3607 | -1.0389 | 0.3972 | 0.3999 |
| KEGG_GALACTOSE_METABOLISM | 26 | -0.3273 | -1.0349 | 0.3918 | 0.4013 |
| KEGG_SYSTEMIC_LUPUS_ERYTHEMATOSUS | 135 | -0.3161 | -1.0333 | 0.4135 | 0.3999 |
| KEGG_ALLOGRAFT_REJECTION | 35 | -0.4565 | -0.9974 | 0.5210 | 0.4365 |
| KEGG_ALPHA_LINOLENIC_ACID_METABOLISM | 19 | -0.3320 | -0.9962 | 0.4338 | 0.4343 |
| KEGG_ASTHMA | 28 | -0.3979 | -0.9841 | 0.5000 | 0.4456 |
| KEGG_GRAFT_VERSUS_HOST_DISEASE | 37 | -0.4470 | -0.9668 | 0.5353 | 0.4624 |
| KEGG_AUTOIMMUNE_THYROID_DISEASE | 50 | -0.3345 | -0.8199 | 0.6618 | 0.6505 |
| KEGG_OLFACTORY_TRANSDUCTION | 383 | -0.1608 | -0.7352 | 0.9854 | 0.7647 |

| **Signaling pathways enriched in HCC samples corresponding ADH5 expression by GSEA based on KEGG** | | | | | |
| --- | --- | --- | --- | --- | --- |
| **KEGG Name** | **Size** | **ES** | **NES** | **NOM p-value** | **FDR q-value** |
| KEGG_DRUG_METABOLISM_CYTOCHROME_P450 | 71 | 0.7541 | 2.2730 | 0.0000 | 0.0000 |
| KEGG_METABOLISM_OF_XENOBIOTICS_BY_CYTOCHROME_P450 | 69 | 0.7286 | 2.2496 | 0.0000 | 0.0000 |
| KEGG_PEROXISOME | 78 | 0.7082 | 2.1829 | 0.0020 | 0.0004 |
| KEGG_FATTY_ACID_METABOLISM | 42 | 0.8624 | 2.1634 | 0.0000 | 0.0003 |
| KEGG_RETINOL_METABOLISM | 64 | 0.7080 | 2.1625 | 0.0000 | 0.0003 |
| KEGG_TYROSINE_METABOLISM | 42 | 0.6371 | 2.0550 | 0.0000 | 0.0007 |
| KEGG_STEROID_HORMONE_BIOSYNTHESIS | 55 | 0.6553 | 2.0317 | 0.0000 | 0.0010 |
| KEGG_PPAR_SIGNALING_PATHWAY | 69 | 0.6256 | 2.0301 | 0.0000 | 0.0010 |
| KEGG_PENTOSE_AND_GLUCURONATE_INTERCONVERSIONS | 28 | 0.7262 | 2.0242 | 0.0000 | 0.0009 |
| KEGG_LYSINE_DEGRADATION | 44 | 0.6509 | 2.0081 | 0.0000 | 0.0013 |
| KEGG_ASCORBATE_AND_ALDARATE_METABOLISM | 25 | 0.7774 | 1.9932 | 0.0000 | 0.0016 |
| KEGG_PROPANOATE_METABOLISM | 32 | 0.7739 | 1.9587 | 0.0000 | 0.0029 |
| KEGG_VALINE_LEUCINE_AND_ISOLEUCINE_DEGRADATION | 43 | 0.7986 | 1.9573 | 0.0020 | 0.0027 |
| KEGG_BUTANOATE_METABOLISM | 34 | 0.7302 | 1.9512 | 0.0000 | 0.0028 |
| KEGG_TRYPTOPHAN_METABOLISM | 40 | 0.6726 | 1.9380 | 0.0000 | 0.0029 |
| KEGG_DRUG_METABOLISM_OTHER_ENZYMES | 51 | 0.5890 | 1.9370 | 0.0000 | 0.0029 |
| KEGG_GLYCOLYSIS_GLUCONEOGENESIS | 62 | 0.5703 | 1.9367 | 0.0000 | 0.0027 |
| KEGG_BETA_ALANINE_METABOLISM | 22 | 0.7316 | 1.9184 | 0.0000 | 0.0034 |
| KEGG_PORPHYRIN_AND_CHLOROPHYLL_METABOLISM | 41 | 0.6239 | 1.9033 | 0.0000 | 0.0041 |
| KEGG_ARGININE_AND_PROLINE_METABOLISM | 54 | 0.5507 | 1.8568 | 0.0019 | 0.0071 |
| KEGG_GLYCINE_SERINE_AND_THREONINE_METABOLISM | 31 | 0.6967 | 1.8415 | 0.0079 | 0.0080 |
| KEGG_PRIMARY_BILE_ACID_BIOSYNTHESIS | 16 | 0.8264 | 1.8233 | 0.0020 | 0.0097 |
| KEGG_HISTIDINE_METABOLISM | 29 | 0.5916 | 1.8153 | 0.0020 | 0.0100 |
| KEGG_PYRUVATE_METABOLISM | 40 | 0.5793 | 1.8040 | 0.0061 | 0.0112 |
| KEGG_BIOSYNTHESIS_OF_UNSATURATED_FATTY_ACIDS | 22 | 0.6596 | 1.7795 | 0.0158 | 0.0138 |
| KEGG_CITRATE_CYCLE_TCA_CYCLE | 31 | 0.6676 | 1.7385 | 0.0208 | 0.0201 |
| KEGG_GLYOXYLATE_AND_DICARBOXYLATE_METABOLISM | 16 | 0.6167 | 1.7351 | 0.0020 | 0.0201 |
| KEGG_STARCH_AND_SUCROSE_METABOLISM | 52 | 0.5239 | 1.7246 | 0.0060 | 0.0217 |
| KEGG_ABC_TRANSPORTERS | 44 | 0.5071 | 1.6588 | 0.0081 | 0.0378 |
| KEGG_CYSTEINE_AND_METHIONINE_METABOLISM | 34 | 0.5016 | 1.6410 | 0.0253 | 0.0420 |
| KEGG_PHENYLALANINE_METABOLISM | 18 | 0.5956 | 1.6373 | 0.0343 | 0.0421 |
| KEGG_ADIPOCYTOKINE_SIGNALING_PATHWAY | 67 | 0.4452 | 1.5442 | 0.0188 | 0.0792 |
| KEGG_ONE_CARBON_POOL_BY_FOLATE | 17 | 0.5355 | 1.5418 | 0.0410 | 0.0782 |
| KEGG_GLYCEROLIPID_METABOLISM | 49 | 0.4364 | 1.5332 | 0.0100 | 0.0797 |
| KEGG_PANTOTHENATE_AND_COA_BIOSYNTHESIS | 16 | 0.5157 | 1.4524 | 0.0706 | 0.1269 |
| KEGG_ALANINE_ASPARTATE_AND_GLUTAMATE_METABOLISM | 32 | 0.4316 | 1.3871 | 0.0966 | 0.1721 |
| KEGG_COMPLEMENT_AND_COAGULATION_CASCADES | 69 | 0.4776 | 1.3596 | 0.1546 | 0.1897 |
| KEGG_LINOLEIC_ACID_METABOLISM | 29 | 0.4242 | 1.3199 | 0.0950 | 0.2194 |
| KEGG_RENIN_ANGIOTENSIN_SYSTEM | 17 | 0.4956 | 1.2749 | 0.1855 | 0.2588 |
| KEGG_STEROID_BIOSYNTHESIS | 17 | 0.5613 | 1.2722 | 0.2395 | 0.2546 |
| KEGG_SELENOAMINO_ACID_METABOLISM | 26 | 0.4010 | 1.2305 | 0.2258 | 0.2924 |
| KEGG_NITROGEN_METABOLISM | 23 | 0.4178 | 1.2118 | 0.1992 | 0.3056 |
| KEGG_TERPENOID_BACKBONE_BIOSYNTHESIS | 15 | 0.4923 | 1.2025 | 0.2596 | 0.3088 |
| KEGG_MATURITY_ONSET_DIABETES_OF_THE_YOUNG | 25 | 0.4237 | 1.1946 | 0.2275 | 0.3104 |
| KEGG_GLUTATHIONE_METABOLISM | 49 | 0.3602 | 1.1739 | 0.2358 | 0.3255 |
| KEGG_NICOTINATE_AND_NICOTINAMIDE_METABOLISM | 24 | 0.3677 | 1.1032 | 0.2982 | 0.4017 |
| KEGG_THYROID_CANCER | 29 | 0.3829 | 1.1026 | 0.3493 | 0.3940 |
| KEGG_BASAL_TRANSCRIPTION_FACTORS | 35 | 0.3715 | 1.0474 | 0.4261 | 0.4523 |
| KEGG_OTHER_GLYCAN_DEGRADATION | 16 | 0.3817 | 1.0124 | 0.4245 | 0.4885 |
| KEGG_ALDOSTERONE_REGULATED_SODIUM_REABSORPTION | 42 | 0.3156 | 0.9954 | 0.4390 | 0.5007 |
| KEGG_REGULATION_OF_AUTOPHAGY | 35 | 0.3139 | 0.9892 | 0.4549 | 0.4986 |
| KEGG_GALACTOSE_METABOLISM | 26 | 0.3107 | 0.9562 | 0.4900 | 0.5338 |
| KEGG_UBIQUITIN_MEDIATED_PROTEOLYSIS | 134 | 0.3085 | 0.9464 | 0.5213 | 0.5367 |
| KEGG_ENDOMETRIAL_CANCER | 52 | 0.3063 | 0.9322 | 0.5398 | 0.5455 |
| KEGG_AMINOACYL_TRNA_BIOSYNTHESIS | 41 | 0.3251 | 0.8716 | 0.5996 | 0.6203 |
| KEGG_GLYCOSAMINOGLYCAN_DEGRADATION | 21 | 0.3102 | 0.8710 | 0.6444 | 0.6101 |
| KEGG_RNA_DEGRADATION | 59 | 0.2914 | 0.8550 | 0.6113 | 0.6212 |
| KEGG_NUCLEOTIDE_EXCISION_REPAIR | 44 | 0.2669 | 0.7436 | 0.7035 | 0.7655 |
| KEGG_OLFACTORY_TRANSDUCTION | 383 | 0.1506 | 0.7025 | 0.9899 | 0.8067 |
| KEGG_CARDIAC_MUSCLE_CONTRACTION | 78 | -0.6138 | -2.0183 | 0.0000 | 0.0316 |
| KEGG_HUNTINGTONS_DISEASE | 180 | -0.5661 | -1.9391 | 0.0020 | 0.0533 |
| KEGG_VIBRIO_CHOLERAE_INFECTION | 54 | -0.5691 | -1.8094 | 0.0056 | 0.1574 |
| KEGG_ALZHEIMERS_DISEASE | 165 | -0.5248 | -1.8020 | 0.0101 | 0.1290 |
| KEGG_PATHOGENIC_ESCHERICHIA_COLI_INFECTION | 56 | -0.6275 | -1.7808 | 0.0019 | 0.1333 |
| KEGG_OXIDATIVE_PHOSPHORYLATION | 131 | -0.5900 | -1.7489 | 0.0314 | 0.1517 |
| KEGG_GLYCOSAMINOGLYCAN_BIOSYNTHESIS_CHONDROITIN_SULFATE | 22 | -0.6625 | -1.7390 | 0.0040 | 0.1429 |
| KEGG_PARKINSONS_DISEASE | 128 | -0.5595 | -1.7238 | 0.0266 | 0.1484 |
| KEGG_FC_GAMMA_R_MEDIATED_PHAGOCYTOSIS | 96 | -0.5637 | -1.6587 | 0.0077 | 0.2312 |
| KEGG_BLADDER_CANCER | 42 | -0.5475 | -1.6186 | 0.0208 | 0.2914 |
| KEGG_DILATED_CARDIOMYOPATHY | 90 | -0.5060 | -1.5715 | 0.0254 | 0.3820 |
| KEGG_PRION_DISEASES | 35 | -0.4966 | -1.5444 | 0.0265 | 0.4167 |
| KEGG_SNARE_INTERACTIONS_IN_VESICULAR_TRANSPORT | 38 | -0.5300 | -1.5377 | 0.0491 | 0.4011 |
| KEGG_CYTOKINE_CYTOKINE_RECEPTOR_INTERACTION | 264 | -0.4425 | -1.4940 | 0.0593 | 0.4881 |
| KEGG_AMYOTROPHIC_LATERAL_SCLEROSIS_ALS | 53 | -0.4249 | -1.4768 | 0.0361 | 0.5037 |
| KEGG_HYPERTROPHIC_CARDIOMYOPATHY_HCM | 83 | -0.4710 | -1.4715 | 0.0397 | 0.4888 |
| KEGG_VASOPRESSIN_REGULATED_WATER_REABSORPTION | 44 | -0.5089 | -1.4633 | 0.0891 | 0.4815 |
| KEGG_GLYCOSPHINGOLIPID_BIOSYNTHESIS_LACTO_AND_NEOLACTO_SERIES | 26 | -0.5037 | -1.4615 | 0.0611 | 0.4596 |
| KEGG_RENAL_CELL_CARCINOMA | 70 | -0.4894 | -1.4519 | 0.0787 | 0.4597 |
| KEGG_FRUCTOSE_AND_MANNOSE_METABOLISM | 33 | -0.4509 | -1.4455 | 0.0473 | 0.4539 |
| KEGG_LYSOSOME | 121 | -0.4279 | -1.3796 | 0.1130 | 0.6117 |
| KEGG_RIBOSOME | 87 | -0.6896 | -1.3757 | 0.1798 | 0.5946 |
| KEGG_REGULATION_OF_ACTIN_CYTOSKELETON | 213 | -0.4208 | -1.3723 | 0.1102 | 0.5784 |
| KEGG_VEGF_SIGNALING_PATHWAY | 76 | -0.4093 | -1.3681 | 0.1172 | 0.5663 |
| KEGG_ENDOCYTOSIS | 181 | -0.4100 | -1.3424 | 0.1543 | 0.6141 |
| KEGG_CELL_ADHESION_MOLECULES_CAMS | 131 | -0.4270 | -1.3400 | 0.1645 | 0.5981 |
| KEGG_O_GLYCAN_BIOSYNTHESIS | 30 | -0.4908 | -1.3389 | 0.1217 | 0.5788 |
| KEGG_CALCIUM_SIGNALING_PATHWAY | 177 | -0.3701 | -1.3282 | 0.1017 | 0.5859 |
| KEGG_MELANOMA | 71 | -0.4050 | -1.3242 | 0.1288 | 0.5758 |
| KEGG_GNRH_SIGNALING_PATHWAY | 101 | -0.3966 | -1.3134 | 0.1489 | 0.5816 |
| KEGG_ECM_RECEPTOR_INTERACTION | 84 | -0.4327 | -1.2945 | 0.1646 | 0.6116 |
| KEGG_FC_EPSILON_RI_SIGNALING_PATHWAY | 79 | -0.3917 | -1.2888 | 0.1715 | 0.6072 |
| KEGG_FOCAL_ADHESION | 199 | -0.4060 | -1.2881 | 0.2008 | 0.5904 |
| KEGG_GLYCEROPHOSPHOLIPID_METABOLISM | 77 | -0.3560 | -1.2815 | 0.1175 | 0.5889 |
| KEGG_LEUKOCYTE_TRANSENDOTHELIAL_MIGRATION | 116 | -0.3961 | -1.2809 | 0.1801 | 0.5737 |
| KEGG_CHEMOKINE_SIGNALING_PATHWAY | 188 | -0.3983 | -1.2751 | 0.2081 | 0.5704 |
| KEGG_LEISHMANIA_INFECTION | 70 | -0.4624 | -1.2717 | 0.2374 | 0.5622 |
| KEGG_DNA_REPLICATION | 36 | -0.5489 | -1.2657 | 0.2596 | 0.5612 |
| KEGG_AXON_GUIDANCE | 129 | -0.3968 | -1.2596 | 0.2012 | 0.5609 |
| KEGG_NOTCH_SIGNALING_PATHWAY | 47 | -0.4370 | -1.2587 | 0.2250 | 0.5486 |
| KEGG_PANCREATIC_CANCER | 70 | -0.4234 | -1.2529 | 0.2495 | 0.5482 |
| KEGG_NEUROACTIVE_LIGAND_RECEPTOR_INTERACTION | 271 | -0.3280 | -1.2511 | 0.0975 | 0.5391 |
| KEGG_SPHINGOLIPID_METABOLISM | 39 | -0.4138 | -1.2460 | 0.2032 | 0.5371 |
| KEGG_TIGHT_JUNCTION | 132 | -0.3595 | -1.2412 | 0.1918 | 0.5346 |
| KEGG_JAK_STAT_SIGNALING_PATHWAY | 155 | -0.3729 | -1.2412 | 0.2060 | 0.5227 |
| KEGG_VASCULAR_SMOOTH_MUSCLE_CONTRACTION | 115 | -0.3642 | -1.2309 | 0.2087 | 0.5307 |
| KEGG_ASTHMA | 28 | -0.4923 | -1.2230 | 0.2556 | 0.5343 |
| KEGG_PROTEASOME | 46 | -0.4703 | -1.2086 | 0.2952 | 0.5505 |
| KEGG_TYPE_II_DIABETES_MELLITUS | 47 | -0.3830 | -1.2070 | 0.1965 | 0.5422 |
| KEGG_TOLL_LIKE_RECEPTOR_SIGNALING_PATHWAY | 102 | -0.3745 | -1.2050 | 0.2536 | 0.5347 |
| KEGG_EPITHELIAL_CELL_SIGNALING_IN_HELICOBACTER_PYLORI_INFECTION | 68 | -0.3817 | -1.1964 | 0.2727 | 0.5407 |
| KEGG_GLIOMA | 65 | -0.3730 | -1.1922 | 0.2402 | 0.5383 |
| KEGG_GAP_JUNCTION | 90 | -0.3800 | -1.1901 | 0.2778 | 0.5321 |
| KEGG_MAPK_SIGNALING_PATHWAY | 267 | -0.3361 | -1.1879 | 0.2447 | 0.5262 |
| KEGG_INOSITOL_PHOSPHATE_METABOLISM | 54 | -0.4123 | -1.1855 | 0.3017 | 0.5208 |
| KEGG_ARACHIDONIC_ACID_METABOLISM | 58 | -0.3287 | -1.1844 | 0.2011 | 0.5130 |
| KEGG_ETHER_LIPID_METABOLISM | 33 | -0.3640 | -1.1844 | 0.2466 | 0.5040 |
| KEGG_PURINE_METABOLISM | 158 | -0.3341 | -1.1803 | 0.2560 | 0.5021 |
| KEGG_HOMOLOGOUS_RECOMBINATION | 28 | -0.4732 | -1.1788 | 0.3327 | 0.4964 |
| KEGG_HEMATOPOIETIC_CELL_LINEAGE | 85 | -0.4123 | -1.1785 | 0.2994 | 0.4889 |
| KEGG_NEUROTROPHIN_SIGNALING_PATHWAY | 126 | -0.3818 | -1.1743 | 0.3002 | 0.4885 |
| KEGG_APOPTOSIS | 87 | -0.3683 | -1.1722 | 0.2889 | 0.4838 |
| KEGG_DORSO_VENTRAL_AXIS_FORMATION | 24 | -0.4305 | -1.1670 | 0.2852 | 0.4847 |
| KEGG_PATHWAYS_IN_CANCER | 325 | -0.3413 | -1.1623 | 0.2805 | 0.4844 |
| KEGG_LONG_TERM_DEPRESSION | 70 | -0.3497 | -1.1580 | 0.2549 | 0.4829 |
| KEGG_PHOSPHATIDYLINOSITOL_SIGNALING_SYSTEM | 76 | -0.3811 | -1.1423 | 0.3162 | 0.5013 |
| KEGG_MELANOGENESIS | 101 | -0.3377 | -1.1336 | 0.3021 | 0.5071 |
| KEGG_RIBOFLAVIN_METABOLISM | 16 | -0.4179 | -1.1335 | 0.3214 | 0.4998 |
| KEGG_PYRIMIDINE_METABOLISM | 98 | -0.3459 | -1.1272 | 0.3365 | 0.5021 |
| KEGG_TASTE_TRANSDUCTION | 51 | -0.3475 | -1.1271 | 0.2933 | 0.4951 |
| KEGG_B_CELL_RECEPTOR_SIGNALING_PATHWAY | 75 | -0.3865 | -1.1203 | 0.3724 | 0.4986 |
| KEGG_INTESTINAL_IMMUNE_NETWORK_FOR_IGA_PRODUCTION | 46 | -0.4570 | -1.1150 | 0.3873 | 0.5011 |
| KEGG_PROSTATE_CANCER | 89 | -0.3455 | -1.1139 | 0.3448 | 0.4957 |
| KEGG_P53_SIGNALING_PATHWAY | 68 | -0.3406 | -1.0999 | 0.3508 | 0.5115 |
| KEGG_NATURAL_KILLER_CELL_MEDIATED_CYTOTOXICITY | 132 | -0.3610 | -1.0974 | 0.3628 | 0.5088 |
| KEGG_NOD_LIKE_RECEPTOR_SIGNALING_PATHWAY | 62 | -0.3773 | -1.0914 | 0.3848 | 0.5112 |
| KEGG_AMINO_SUGAR_AND_NUCLEOTIDE_SUGAR_METABOLISM | 43 | -0.3340 | -1.0884 | 0.3544 | 0.5091 |
| KEGG_LONG_TERM_POTENTIATION | 70 | -0.3278 | -1.0598 | 0.4176 | 0.5471 |
| KEGG_CHRONIC_MYELOID_LEUKEMIA | 73 | -0.3547 | -1.0576 | 0.4066 | 0.5438 |
| KEGG_PROGESTERONE_MEDIATED_OOCYTE_MATURATION | 85 | -0.3320 | -1.0533 | 0.3951 | 0.5435 |
| KEGG_ANTIGEN_PROCESSING_AND_PRESENTATION | 81 | -0.3957 | -1.0526 | 0.4549 | 0.5378 |
| KEGG_ACUTE_MYELOID_LEUKEMIA | 57 | -0.3432 | -1.0380 | 0.4141 | 0.5544 |
| KEGG_T_CELL_RECEPTOR_SIGNALING_PATHWAY | 108 | -0.3517 | -1.0356 | 0.4430 | 0.5514 |
| KEGG_ERBB_SIGNALING_PATHWAY | 87 | -0.3243 | -1.0351 | 0.3977 | 0.5456 |
| KEGG_INSULIN_SIGNALING_PATHWAY | 137 | -0.2907 | -1.0297 | 0.4163 | 0.5467 |
| KEGG_VIRAL_MYOCARDITIS | 68 | -0.3566 | -1.0204 | 0.4442 | 0.5543 |
| KEGG_OOCYTE_MEIOSIS | 112 | -0.3156 | -1.0204 | 0.4545 | 0.5480 |
| KEGG_GLYCOSAMINOGLYCAN_BIOSYNTHESIS_HEPARAN_SULFATE | 26 | -0.3600 | -1.0096 | 0.4589 | 0.5574 |
| KEGG_CYTOSOLIC_DNA_SENSING_PATHWAY | 55 | -0.3269 | -1.0061 | 0.4600 | 0.5568 |
| KEGG_SMALL_CELL_LUNG_CANCER | 84 | -0.3276 | -1.0015 | 0.4511 | 0.5570 |
| KEGG_PROTEIN_EXPORT | 24 | -0.3742 | -0.9962 | 0.4635 | 0.5589 |
| KEGG_PROXIMAL_TUBULE_BICARBONATE_RECLAMATION | 23 | -0.3364 | -0.9888 | 0.4485 | 0.5640 |
| KEGG_TYPE_I_DIABETES_MELLITUS | 41 | -0.4080 | -0.9868 | 0.4767 | 0.5612 |
| KEGG_GLYCOSAMINOGLYCAN_BIOSYNTHESIS_KERATAN_SULFATE | 15 | -0.3851 | -0.9746 | 0.4841 | 0.5728 |
| KEGG_MTOR_SIGNALING_PATHWAY | 52 | -0.3213 | -0.9683 | 0.4930 | 0.5756 |
| KEGG_NON_SMALL_CELL_LUNG_CANCER | 54 | -0.3179 | -0.9656 | 0.4866 | 0.5737 |
| KEGG_N_GLYCAN_BIOSYNTHESIS | 46 | -0.3271 | -0.9635 | 0.5271 | 0.5709 |
| KEGG_HEDGEHOG_SIGNALING_PATHWAY | 56 | -0.3040 | -0.9611 | 0.5151 | 0.5684 |
| KEGG_RNA_POLYMERASE | 29 | -0.3679 | -0.9584 | 0.5389 | 0.5666 |
| KEGG_ARRHYTHMOGENIC_RIGHT_VENTRICULAR_CARDIOMYOPATHY_ARVC | 74 | -0.3058 | -0.9405 | 0.5602 | 0.5866 |
| KEGG_CELL_CYCLE | 124 | -0.3286 | -0.9278 | 0.5585 | 0.5995 |
| KEGG_ALPHA_LINOLENIC_ACID_METABOLISM | 19 | -0.3083 | -0.9206 | 0.5793 | 0.6041 |
| KEGG_COLORECTAL_CANCER | 62 | -0.3060 | -0.9196 | 0.5285 | 0.5999 |
| KEGG_TGF_BETA_SIGNALING_PATHWAY | 85 | -0.2918 | -0.9101 | 0.5460 | 0.6078 |
| KEGG_WNT_SIGNALING_PATHWAY | 150 | -0.2692 | -0.8957 | 0.5977 | 0.6229 |
| KEGG_GLYCOSPHINGOLIPID_BIOSYNTHESIS_GANGLIO_SERIES | 15 | -0.3342 | -0.8896 | 0.6031 | 0.6257 |
| KEGG_ALLOGRAFT_REJECTION | 35 | -0.3985 | -0.8772 | 0.6093 | 0.6377 |
| KEGG_PENTOSE_PHOSPHATE_PATHWAY | 27 | -0.2884 | -0.8511 | 0.6520 | 0.6705 |
| KEGG_GRAFT_VERSUS_HOST_DISEASE | 37 | -0.3936 | -0.8486 | 0.6491 | 0.6681 |
| KEGG_RIG_I_LIKE_RECEPTOR_SIGNALING_PATHWAY | 71 | -0.2671 | -0.8483 | 0.6431 | 0.6623 |
| KEGG_ADHERENS_JUNCTION | 73 | -0.2852 | -0.8475 | 0.6167 | 0.6577 |
| KEGG_PRIMARY_IMMUNODEFICIENCY | 35 | -0.3717 | -0.8347 | 0.6569 | 0.6701 |
| KEGG_BASAL_CELL_CARCINOMA | 55 | -0.2591 | -0.8129 | 0.7229 | 0.6943 |
| KEGG_SPLICEOSOME | 127 | -0.2958 | -0.7774 | 0.6911 | 0.7388 |
| KEGG_BASE_EXCISION_REPAIR | 35 | -0.2793 | -0.7556 | 0.7328 | 0.7619 |
| KEGG_GLYCOSYLPHOSPHATIDYLINOSITOL_GPI_ANCHOR_BIOSYNTHESIS | 25 | -0.2790 | -0.7539 | 0.7099 | 0.7577 |
| KEGG_MISMATCH_REPAIR | 23 | -0.3045 | -0.7191 | 0.7505 | 0.7991 |
| KEGG_SYSTEMIC_LUPUS_ERYTHEMATOSUS | 135 | -0.2089 | -0.6871 | 0.8395 | 0.8319 |
| KEGG_AUTOIMMUNE_THYROID_DISEASE | 50 | -0.2334 | -0.5946 | 0.8271 | 0.9230 |

| **Signaling pathways enriched in HCC samples corresponding ADH6 expression by GSEA based on KEGG** | | | | | |
| --- | --- | --- | --- | --- | --- |
| **KEGG Name** | **Size** | **ES** | **NES** | **NOM p-value** | **FDR q-value** |
| KEGG_DRUG_METABOLISM_CYTOCHROME_P450 | 71 | 0.7843 | 2.3252 | 0.0000 | 0.0000 |
| KEGG_METABOLISM_OF_XENOBIOTICS_BY_CYTOCHROME_P450 | 69 | 0.7531 | 2.3136 | 0.0000 | 0.0000 |
| KEGG_RETINOL_METABOLISM | 64 | 0.7627 | 2.2921 | 0.0000 | 0.0000 |
| KEGG_TYROSINE_METABOLISM | 42 | 0.6987 | 2.2232 | 0.0000 | 0.0001 |
| KEGG_PEROXISOME | 78 | 0.7263 | 2.1685 | 0.0000 | 0.0001 |
| KEGG_FATTY_ACID_METABOLISM | 42 | 0.8849 | 2.1667 | 0.0000 | 0.0001 |
| KEGG_STEROID_HORMONE_BIOSYNTHESIS | 55 | 0.7054 | 2.1659 | 0.0000 | 0.0001 |
| KEGG_PPAR_SIGNALING_PATHWAY | 69 | 0.6525 | 2.1029 | 0.0000 | 0.0001 |
| KEGG_TRYPTOPHAN_METABOLISM | 40 | 0.7232 | 2.0563 | 0.0000 | 0.0005 |
| KEGG_GLYCINE_SERINE_AND_THREONINE_METABOLISM | 31 | 0.8020 | 2.0475 | 0.0000 | 0.0006 |
| KEGG_PRIMARY_BILE_ACID_BIOSYNTHESIS | 16 | 0.9118 | 2.0229 | 0.0000 | 0.0008 |
| KEGG_BUTANOATE_METABOLISM | 34 | 0.7207 | 1.9263 | 0.0080 | 0.0027 |
| KEGG_ASCORBATE_AND_ALDARATE_METABOLISM | 25 | 0.7373 | 1.9227 | 0.0019 | 0.0027 |
| KEGG_HISTIDINE_METABOLISM | 29 | 0.6365 | 1.8937 | 0.0000 | 0.0046 |
| KEGG_VALINE_LEUCINE_AND_ISOLEUCINE_DEGRADATION | 43 | 0.7976 | 1.8918 | 0.0041 | 0.0044 |
| KEGG_BETA_ALANINE_METABOLISM | 22 | 0.7156 | 1.8107 | 0.0079 | 0.0107 |
| KEGG_PROPANOATE_METABOLISM | 32 | 0.6979 | 1.8053 | 0.0137 | 0.0107 |
| KEGG_PHENYLALANINE_METABOLISM | 18 | 0.6590 | 1.7948 | 0.0020 | 0.0111 |
| KEGG_ARGININE_AND_PROLINE_METABOLISM | 54 | 0.5436 | 1.7818 | 0.0020 | 0.0119 |
| KEGG_DRUG_METABOLISM_OTHER_ENZYMES | 51 | 0.5373 | 1.7497 | 0.0135 | 0.0159 |
| KEGG_LINOLEIC_ACID_METABOLISM | 29 | 0.5617 | 1.7396 | 0.0097 | 0.0168 |
| KEGG_PENTOSE_AND_GLUCURONATE_INTERCONVERSIONS | 28 | 0.6124 | 1.6970 | 0.0350 | 0.0243 |
| KEGG_COMPLEMENT_AND_COAGULATION_CASCADES | 69 | 0.5668 | 1.5974 | 0.0575 | 0.0547 |
| KEGG_PORPHYRIN_AND_CHLOROPHYLL_METABOLISM | 41 | 0.5274 | 1.5796 | 0.0287 | 0.0592 |
| KEGG_ALANINE_ASPARTATE_AND_GLUTAMATE_METABOLISM | 32 | 0.4863 | 1.5634 | 0.0295 | 0.0636 |
| KEGG_GLYCOLYSIS_GLUCONEOGENESIS | 62 | 0.4473 | 1.5352 | 0.0502 | 0.0745 |
| KEGG_GLYOXYLATE_AND_DICARBOXYLATE_METABOLISM | 16 | 0.5624 | 1.5241 | 0.0429 | 0.0773 |
| KEGG_PYRUVATE_METABOLISM | 40 | 0.4755 | 1.4922 | 0.0780 | 0.0909 |
| KEGG_STARCH_AND_SUCROSE_METABOLISM | 52 | 0.4485 | 1.4761 | 0.0512 | 0.0967 |
| KEGG_ONE_CARBON_POOL_BY_FOLATE | 17 | 0.5155 | 1.4730 | 0.0609 | 0.0951 |
| KEGG_NITROGEN_METABOLISM | 23 | 0.4932 | 1.4621 | 0.0648 | 0.0978 |
| KEGG_BIOSYNTHESIS_OF_UNSATURATED_FATTY_ACIDS | 22 | 0.5395 | 1.4310 | 0.0918 | 0.1135 |
| KEGG_LYSINE_DEGRADATION | 44 | 0.4598 | 1.3458 | 0.1715 | 0.1664 |
| KEGG_ARACHIDONIC_ACID_METABOLISM | 58 | 0.3603 | 1.3218 | 0.0688 | 0.1803 |
| KEGG_ABC_TRANSPORTERS | 44 | 0.3994 | 1.2618 | 0.1744 | 0.2253 |
| KEGG_GLYCEROLIPID_METABOLISM | 49 | 0.3529 | 1.2443 | 0.1502 | 0.2337 |
| KEGG_PANTOTHENATE_AND_COA_BIOSYNTHESIS | 16 | 0.4452 | 1.2300 | 0.1911 | 0.2398 |
| KEGG_ADIPOCYTOKINE_SIGNALING_PATHWAY | 67 | 0.3340 | 1.1575 | 0.2435 | 0.3002 |
| KEGG_TERPENOID_BACKBONE_BIOSYNTHESIS | 15 | 0.4664 | 1.1509 | 0.3208 | 0.2989 |
| KEGG_RENIN_ANGIOTENSIN_SYSTEM | 17 | 0.4406 | 1.1328 | 0.2843 | 0.3100 |
| KEGG_GLUTATHIONE_METABOLISM | 49 | 0.3334 | 1.1010 | 0.3067 | 0.3334 |
| KEGG_STEROID_BIOSYNTHESIS | 17 | 0.4459 | 1.0139 | 0.4406 | 0.4192 |
| KEGG_MATURITY_ONSET_DIABETES_OF_THE_YOUNG | 25 | 0.3052 | 0.8746 | 0.6154 | 0.5811 |
| KEGG_CITRATE_CYCLE_TCA_CYCLE | 31 | 0.2747 | 0.7252 | 0.7198 | 0.7700 |
| KEGG_FC_GAMMA_R_MEDIATED_PHAGOCYTOSIS | 96 | -0.6710 | -1.9715 | 0.0000 | 0.1063 |
| KEGG_PATHOGENIC_ESCHERICHIA_COLI_INFECTION | 56 | -0.6667 | -1.9498 | 0.0019 | 0.0704 |
| KEGG_GLYCOSAMINOGLYCAN_BIOSYNTHESIS_CHONDROITIN_SULFATE | 22 | -0.7309 | -1.9320 | 0.0000 | 0.0606 |
| KEGG_REGULATION_OF_ACTIN_CYTOSKELETON | 213 | -0.5665 | -1.8903 | 0.0000 | 0.0675 |
| KEGG_INOSITOL_PHOSPHATE_METABOLISM | 54 | -0.6386 | -1.8725 | 0.0000 | 0.0658 |
| KEGG_ENDOCYTOSIS | 181 | -0.5681 | -1.8509 | 0.0000 | 0.0709 |
| KEGG_RENAL_CELL_CARCINOMA | 70 | -0.6149 | -1.8329 | 0.0000 | 0.0729 |
| KEGG_AXON_GUIDANCE | 129 | -0.5786 | -1.8278 | 0.0020 | 0.0662 |
| KEGG_PHOSPHATIDYLINOSITOL_SIGNALING_SYSTEM | 76 | -0.5988 | -1.8102 | 0.0000 | 0.0711 |
| KEGG_EPITHELIAL_CELL_SIGNALING_IN_HELICOBACTER_PYLORI_INFECTION | 68 | -0.5919 | -1.8042 | 0.0020 | 0.0676 |
| KEGG_FC_EPSILON_RI_SIGNALING_PATHWAY | 79 | -0.5638 | -1.8030 | 0.0000 | 0.0617 |
| KEGG_VEGF_SIGNALING_PATHWAY | 76 | -0.5525 | -1.7907 | 0.0000 | 0.0655 |
| KEGG_NEUROTROPHIN_SIGNALING_PATHWAY | 126 | -0.5721 | -1.7836 | 0.0076 | 0.0651 |
| KEGG_BLADDER_CANCER | 42 | -0.5856 | -1.7726 | 0.0077 | 0.0667 |
| KEGG_VIBRIO_CHOLERAE_INFECTION | 54 | -0.5734 | -1.7708 | 0.0019 | 0.0632 |
| KEGG_NOD_LIKE_RECEPTOR_SIGNALING_PATHWAY | 62 | -0.6093 | -1.7654 | 0.0020 | 0.0622 |
| KEGG_SNARE_INTERACTIONS_IN_VESICULAR_TRANSPORT | 38 | -0.6087 | -1.7644 | 0.0039 | 0.0591 |
| KEGG_DORSO_VENTRAL_AXIS_FORMATION | 24 | -0.6565 | -1.7580 | 0.0058 | 0.0587 |
| KEGG_GLYCOSPHINGOLIPID_BIOSYNTHESIS_LACTO_AND_NEOLACTO_SERIES | 26 | -0.6039 | -1.7560 | 0.0020 | 0.0565 |
| KEGG_CYTOSOLIC_DNA_SENSING_PATHWAY | 55 | -0.5683 | -1.7557 | 0.0074 | 0.0539 |
| KEGG_SMALL_CELL_LUNG_CANCER | 84 | -0.5744 | -1.7551 | 0.0020 | 0.0515 |
| KEGG_NOTCH_SIGNALING_PATHWAY | 47 | -0.6107 | -1.7546 | 0.0040 | 0.0495 |
| KEGG_VASOPRESSIN_REGULATED_WATER_REABSORPTION | 44 | -0.6136 | -1.7539 | 0.0040 | 0.0475 |
| KEGG_B_CELL_RECEPTOR_SIGNALING_PATHWAY | 75 | -0.5975 | -1.7420 | 0.0189 | 0.0521 |
| KEGG_OOCYTE_MEIOSIS | 112 | -0.5394 | -1.7367 | 0.0095 | 0.0526 |
| KEGG_MELANOMA | 71 | -0.5177 | -1.7275 | 0.0020 | 0.0551 |
| KEGG_MAPK_SIGNALING_PATHWAY | 267 | -0.4909 | -1.7252 | 0.0060 | 0.0537 |
| KEGG_HOMOLOGOUS_RECOMBINATION | 28 | -0.6940 | -1.7228 | 0.0073 | 0.0528 |
| KEGG_PROGESTERONE_MEDIATED_OOCYTE_MATURATION | 85 | -0.5436 | -1.7224 | 0.0075 | 0.0511 |
| KEGG_PATHWAYS_IN_CANCER | 325 | -0.5007 | -1.7179 | 0.0098 | 0.0512 |
| KEGG_CHRONIC_MYELOID_LEUKEMIA | 73 | -0.5774 | -1.7168 | 0.0059 | 0.0502 |
| KEGG_PANCREATIC_CANCER | 70 | -0.5807 | -1.7115 | 0.0098 | 0.0508 |
| KEGG_LEISHMANIA_INFECTION | 70 | -0.6191 | -1.7029 | 0.0215 | 0.0524 |
| KEGG_AMINO_SUGAR_AND_NUCLEOTIDE_SUGAR_METABOLISM | 43 | -0.5388 | -1.7017 | 0.0137 | 0.0514 |
| KEGG_MTOR_SIGNALING_PATHWAY | 52 | -0.5549 | -1.6963 | 0.0040 | 0.0525 |
| KEGG_PURINE_METABOLISM | 158 | -0.4708 | -1.6950 | 0.0076 | 0.0516 |
| KEGG_JAK_STAT_SIGNALING_PATHWAY | 155 | -0.5112 | -1.6918 | 0.0079 | 0.0516 |
| KEGG_APOPTOSIS | 87 | -0.5431 | -1.6895 | 0.0158 | 0.0511 |
| KEGG_TOLL_LIKE_RECEPTOR_SIGNALING_PATHWAY | 102 | -0.5349 | -1.6872 | 0.0214 | 0.0510 |
| KEGG_FOCAL_ADHESION | 199 | -0.5315 | -1.6869 | 0.0218 | 0.0499 |
| KEGG_LYSOSOME | 121 | -0.5342 | -1.6857 | 0.0247 | 0.0492 |
| KEGG_T_CELL_RECEPTOR_SIGNALING_PATHWAY | 108 | -0.5862 | -1.6847 | 0.0097 | 0.0483 |
| KEGG_GAP_JUNCTION | 90 | -0.5426 | -1.6784 | 0.0120 | 0.0500 |
| KEGG_CYTOKINE_CYTOKINE_RECEPTOR_INTERACTION | 264 | -0.5148 | -1.6774 | 0.0117 | 0.0492 |
| KEGG_GLIOMA | 65 | -0.5227 | -1.6761 | 0.0117 | 0.0484 |
| KEGG_GNRH_SIGNALING_PATHWAY | 101 | -0.5127 | -1.6760 | 0.0080 | 0.0474 |
| KEGG_PRION_DISEASES | 35 | -0.5442 | -1.6546 | 0.0231 | 0.0556 |
| KEGG_CHEMOKINE_SIGNALING_PATHWAY | 188 | -0.5305 | -1.6540 | 0.0253 | 0.0548 |
| KEGG_ERBB_SIGNALING_PATHWAY | 87 | -0.5438 | -1.6512 | 0.0145 | 0.0547 |
| KEGG_GLYCEROPHOSPHOLIPID_METABOLISM | 77 | -0.4602 | -1.6454 | 0.0059 | 0.0563 |
| KEGG_CELL_CYCLE | 124 | -0.5913 | -1.6312 | 0.0251 | 0.0614 |
| KEGG_FRUCTOSE_AND_MANNOSE_METABOLISM | 33 | -0.5061 | -1.6261 | 0.0118 | 0.0625 |
| KEGG_INSULIN_SIGNALING_PATHWAY | 137 | -0.4571 | -1.6214 | 0.0210 | 0.0634 |
| KEGG_NATURAL_KILLER_CELL_MEDIATED_CYTOTOXICITY | 132 | -0.5312 | -1.6202 | 0.0345 | 0.0630 |
| KEGG_ANTIGEN_PROCESSING_AND_PRESENTATION | 81 | -0.5755 | -1.5943 | 0.0670 | 0.0744 |
| KEGG_DNA_REPLICATION | 36 | -0.6997 | -1.5789 | 0.0433 | 0.0819 |
| KEGG_RIG_I_LIKE_RECEPTOR_SIGNALING_PATHWAY | 71 | -0.4941 | -1.5713 | 0.0236 | 0.0851 |
| KEGG_P53_SIGNALING_PATHWAY | 68 | -0.4792 | -1.5635 | 0.0319 | 0.0875 |
| KEGG_HUNTINGTONS_DISEASE | 180 | -0.4677 | -1.5537 | 0.0523 | 0.0918 |
| KEGG_LONG_TERM_DEPRESSION | 70 | -0.4707 | -1.5482 | 0.0197 | 0.0937 |
| KEGG_MISMATCH_REPAIR | 23 | -0.6777 | -1.5465 | 0.0424 | 0.0929 |
| KEGG_LEUKOCYTE_TRANSENDOTHELIAL_MIGRATION | 116 | -0.4785 | -1.5455 | 0.0468 | 0.0920 |
| KEGG_PROSTATE_CANCER | 89 | -0.4863 | -1.5440 | 0.0423 | 0.0915 |
| KEGG_DILATED_CARDIOMYOPATHY | 90 | -0.4958 | -1.5410 | 0.0513 | 0.0918 |
| KEGG_HYPERTROPHIC_CARDIOMYOPATHY_HCM | 83 | -0.4857 | -1.5317 | 0.0423 | 0.0956 |
| KEGG_TGF_BETA_SIGNALING_PATHWAY | 85 | -0.5002 | -1.5309 | 0.0527 | 0.0946 |
| KEGG_PYRIMIDINE_METABOLISM | 98 | -0.4704 | -1.5190 | 0.0483 | 0.1005 |
| KEGG_CARDIAC_MUSCLE_CONTRACTION | 78 | -0.4672 | -1.5168 | 0.0525 | 0.1003 |
| KEGG_LONG_TERM_POTENTIATION | 70 | -0.4649 | -1.5034 | 0.0498 | 0.1076 |
| KEGG_MELANOGENESIS | 101 | -0.4472 | -1.5011 | 0.0423 | 0.1072 |
| KEGG_TYPE_II_DIABETES_MELLITUS | 47 | -0.4682 | -1.5004 | 0.0395 | 0.1059 |
| KEGG_SPLICEOSOME | 127 | -0.5647 | -1.4878 | 0.0914 | 0.1117 |
| KEGG_CELL_ADHESION_MOLECULES_CAMS | 131 | -0.4770 | -1.4860 | 0.1048 | 0.1114 |
| KEGG_NON_SMALL_CELL_LUNG_CANCER | 54 | -0.4932 | -1.4820 | 0.0558 | 0.1128 |
| KEGG_VASCULAR_SMOOTH_MUSCLE_CONTRACTION | 115 | -0.4434 | -1.4814 | 0.0747 | 0.1116 |
| KEGG_BASAL_TRANSCRIPTION_FACTORS | 35 | -0.5351 | -1.4794 | 0.0895 | 0.1114 |
| KEGG_UBIQUITIN_MEDIATED_PROTEOLYSIS | 134 | -0.4844 | -1.4706 | 0.0985 | 0.1156 |
| KEGG_ALZHEIMERS_DISEASE | 165 | -0.4341 | -1.4659 | 0.0840 | 0.1176 |
| KEGG_WNT_SIGNALING_PATHWAY | 150 | -0.4451 | -1.4610 | 0.0667 | 0.1192 |
| KEGG_ECM_RECEPTOR_INTERACTION | 84 | -0.4920 | -1.4573 | 0.0989 | 0.1199 |
| KEGG_ACUTE_MYELOID_LEUKEMIA | 57 | -0.4819 | -1.4550 | 0.0812 | 0.1202 |
| KEGG_GLYCOSAMINOGLYCAN_BIOSYNTHESIS_KERATAN_SULFATE | 15 | -0.5699 | -1.4497 | 0.0769 | 0.1225 |
| KEGG_HEMATOPOIETIC_CELL_LINEAGE | 85 | -0.5059 | -1.4431 | 0.0973 | 0.1249 |
| KEGG_COLORECTAL_CANCER | 62 | -0.4848 | -1.4382 | 0.1089 | 0.1272 |
| KEGG_CALCIUM_SIGNALING_PATHWAY | 177 | -0.4012 | -1.4330 | 0.0724 | 0.1292 |
| KEGG_ETHER_LIPID_METABOLISM | 33 | -0.4490 | -1.4308 | 0.0639 | 0.1293 |
| KEGG_INTESTINAL_IMMUNE_NETWORK_FOR_IGA_PRODUCTION | 46 | -0.5833 | -1.4295 | 0.1260 | 0.1284 |
| KEGG_N_GLYCAN_BIOSYNTHESIS | 46 | -0.4803 | -1.4202 | 0.1015 | 0.1334 |
| KEGG_AMYOTROPHIC_LATERAL_SCLEROSIS_ALS | 53 | -0.4114 | -1.4155 | 0.0601 | 0.1352 |
| KEGG_TYPE_I_DIABETES_MELLITUS | 41 | -0.5736 | -1.4128 | 0.1531 | 0.1355 |
| KEGG_TIGHT_JUNCTION | 132 | -0.4100 | -1.4122 | 0.0996 | 0.1344 |
| KEGG_AMINOACYL_TRNA_BIOSYNTHESIS | 41 | -0.5266 | -1.4044 | 0.1460 | 0.1386 |
| KEGG_TASTE_TRANSDUCTION | 51 | -0.4362 | -1.3974 | 0.0739 | 0.1419 |
| KEGG_NUCLEOTIDE_EXCISION_REPAIR | 44 | -0.5233 | -1.3926 | 0.1300 | 0.1437 |
| KEGG_RIBOFLAVIN_METABOLISM | 16 | -0.5278 | -1.3904 | 0.0897 | 0.1435 |
| KEGG_RNA_DEGRADATION | 59 | -0.4828 | -1.3860 | 0.1417 | 0.1452 |
| KEGG_VIRAL_MYOCARDITIS | 68 | -0.4693 | -1.3796 | 0.1537 | 0.1484 |
| KEGG_HEDGEHOG_SIGNALING_PATHWAY | 56 | -0.4384 | -1.3654 | 0.0922 | 0.1570 |
| KEGG_RNA_POLYMERASE | 29 | -0.5108 | -1.3520 | 0.1401 | 0.1653 |
| KEGG_SPHINGOLIPID_METABOLISM | 39 | -0.4477 | -1.3382 | 0.1563 | 0.1750 |
| KEGG_GLYCOSPHINGOLIPID_BIOSYNTHESIS_GANGLIO_SERIES | 15 | -0.5169 | -1.3354 | 0.1361 | 0.1756 |
| KEGG_PRIMARY_IMMUNODEFICIENCY | 35 | -0.6062 | -1.3291 | 0.1785 | 0.1785 |
| KEGG_O_GLYCAN_BIOSYNTHESIS | 30 | -0.4926 | -1.3289 | 0.1277 | 0.1769 |
| KEGG_BASE_EXCISION_REPAIR | 35 | -0.4812 | -1.2906 | 0.2300 | 0.2067 |
| KEGG_NEUROACTIVE_LIGAND_RECEPTOR_INTERACTION | 271 | -0.3339 | -1.2622 | 0.1022 | 0.2287 |
| KEGG_ADHERENS_JUNCTION | 73 | -0.4286 | -1.2591 | 0.2391 | 0.2291 |
| KEGG_ARRHYTHMOGENIC_RIGHT_VENTRICULAR_CARDIOMYOPATHY_ARVC | 74 | -0.4082 | -1.2576 | 0.1733 | 0.2282 |
| KEGG_ASTHMA | 28 | -0.4951 | -1.2460 | 0.2417 | 0.2361 |
| KEGG_REGULATION_OF_AUTOPHAGY | 35 | -0.3889 | -1.2097 | 0.2349 | 0.2676 |
| KEGG_GLYCOSAMINOGLYCAN_BIOSYNTHESIS_HEPARAN_SULFATE | 26 | -0.4307 | -1.2025 | 0.2505 | 0.2722 |
| KEGG_PROTEIN_EXPORT | 24 | -0.4520 | -1.2003 | 0.2611 | 0.2716 |
| KEGG_GALACTOSE_METABOLISM | 26 | -0.3833 | -1.1999 | 0.2148 | 0.2696 |
| KEGG_SELENOAMINO_ACID_METABOLISM | 26 | -0.3941 | -1.1839 | 0.2622 | 0.2833 |
| KEGG_PROTEASOME | 46 | -0.4732 | -1.1723 | 0.3359 | 0.2923 |
| KEGG_ALLOGRAFT_REJECTION | 35 | -0.5309 | -1.1714 | 0.3281 | 0.2907 |
| KEGG_GRAFT_VERSUS_HOST_DISEASE | 37 | -0.5190 | -1.1451 | 0.3647 | 0.3146 |
| KEGG_OXIDATIVE_PHOSPHORYLATION | 131 | -0.3959 | -1.1450 | 0.3353 | 0.3119 |
| KEGG_GLYCOSYLPHOSPHATIDYLINOSITOL_GPI_ANCHOR_BIOSYNTHESIS | 25 | -0.4286 | -1.1408 | 0.3333 | 0.3132 |
| KEGG_OTHER_GLYCAN_DEGRADATION | 16 | -0.4465 | -1.1278 | 0.3046 | 0.3239 |
| KEGG_PENTOSE_PHOSPHATE_PATHWAY | 27 | -0.3780 | -1.1273 | 0.2883 | 0.3218 |
| KEGG_PARKINSONS_DISEASE | 128 | -0.3743 | -1.1219 | 0.3622 | 0.3247 |
| KEGG_BASAL_CELL_CARCINOMA | 55 | -0.3521 | -1.1034 | 0.3246 | 0.3410 |
| KEGG_AUTOIMMUNE_THYROID_DISEASE | 50 | -0.4251 | -1.0969 | 0.3638 | 0.3448 |
| KEGG_ENDOMETRIAL_CANCER | 52 | -0.3576 | -1.0696 | 0.3870 | 0.3708 |
| KEGG_CYSTEINE_AND_METHIONINE_METABOLISM | 34 | -0.3314 | -1.0460 | 0.3770 | 0.3942 |
| KEGG_OLFACTORY_TRANSDUCTION | 383 | -0.2156 | -0.9891 | 0.4618 | 0.4576 |
| KEGG_NICOTINATE_AND_NICOTINAMIDE_METABOLISM | 24 | -0.3201 | -0.9804 | 0.4892 | 0.4646 |
| KEGG_THYROID_CANCER | 29 | -0.3446 | -0.9691 | 0.4940 | 0.4750 |
| KEGG_ALDOSTERONE_REGULATED_SODIUM_REABSORPTION | 42 | -0.2964 | -0.9533 | 0.4921 | 0.4906 |
| KEGG_PROXIMAL_TUBULE_BICARBONATE_RECLAMATION | 23 | -0.3107 | -0.9184 | 0.5755 | 0.5312 |
| KEGG_SYSTEMIC_LUPUS_ERYTHEMATOSUS | 135 | -0.2626 | -0.8553 | 0.6277 | 0.6110 |
| KEGG_GLYCOSAMINOGLYCAN_DEGRADATION | 21 | -0.3082 | -0.8432 | 0.6601 | 0.6228 |
| KEGG_ALPHA_LINOLENIC_ACID_METABOLISM | 19 | -0.2745 | -0.8217 | 0.7460 | 0.6471 |
| KEGG_RIBOSOME | 87 | -0.3281 | -0.6798 | 0.7540 | 0.8319 |
