# Supplementary material for: Prognostic implications of alcohol dehydrogenases in hepatocellular carcinoma
Source: BMC Cancer. 2020 Dec 7;20:1204. doi: 10.1186/s12885-020-07689-1 (PMC7720489; doi:10.1186/s12885-020-07689-1)
Supplement: Supplementary file 4 — Additional file 4: Table S4. Signaling pathways enriched in HCC samples corresponding ADHs expression by GSEA based on PID. [file 12885_2020_7689_MOESM4_ESM.docx]

**Table S4. Signaling pathways enriched in HCC samples corresponding ADHs expression by GSEA based on PID.**

| **Signaling pathways enriched in HCC samples corresponding ADH1A expression by GSEA based on PID** | | | | | |
| --- | --- | --- | --- | --- | --- |
| **PID Name** | **Size** | **ES** | **NES** | **NOM p-value** | **FDR q-value** |
| PID_HNF3B_PATHWAY | 45 | 0.4878 | 1.6185 | 0.0203 | 0.0462 |
| PID_HNF3A_PATHWAY | 44 | 0.3138 | 1.0345 | 0.3796 | 0.4416 |
| PID_INTEGRIN2_PATHWAY | 28 | 0.3291 | 0.8779 | 0.5976 | 0.4589 |
| PID_MTOR_4PATHWAY | 69 | -0.6658 | -2.0492 | 0.0000 | 0.0055 |
| PID_ARF_3PATHWAY | 19 | -0.8405 | -2.0481 | 0.0000 | 0.0028 |
| PID_PDGFRB_PATHWAY | 129 | -0.6712 | -2.0206 | 0.0000 | 0.0025 |
| PID_NOTCH_PATHWAY | 59 | -0.6778 | -2.0118 | 0.0000 | 0.0021 |
| PID_NETRIN_PATHWAY | 32 | -0.7316 | -2.0114 | 0.0000 | 0.0017 |
| PID_HIF1A_PATHWAY | 19 | -0.7618 | -2.0021 | 0.0000 | 0.0018 |
| PID_INSULIN_GLUCOSE_PATHWAY | 26 | -0.7114 | -1.9973 | 0.0000 | 0.0018 |
| PID_EPHB_FWD_PATHWAY | 40 | -0.7044 | -1.9971 | 0.0000 | 0.0016 |
| PID_AR_NONGENOMIC_PATHWAY | 31 | -0.6671 | -1.9830 | 0.0000 | 0.0015 |
| PID_ERBB1_DOWNSTREAM_PATHWAY | 105 | -0.6464 | -1.9759 | 0.0000 | 0.0017 |
| PID_MYC_ACTIV_PATHWAY | 79 | -0.6624 | -1.9720 | 0.0000 | 0.0017 |
| PID_PRL_SIGNALING_EVENTS_PATHWAY | 23 | -0.6902 | -1.9685 | 0.0000 | 0.0017 |
| PID_VEGFR1_2_PATHWAY | 69 | -0.6715 | -1.9661 | 0.0000 | 0.0016 |
| PID_HDAC_CLASSII_PATHWAY | 34 | -0.6983 | -1.9647 | 0.0000 | 0.0016 |
| PID_CDC42_PATHWAY | 70 | -0.6537 | -1.9638 | 0.0000 | 0.0016 |
| PID_ILK_PATHWAY | 45 | -0.7059 | -1.9550 | 0.0000 | 0.0019 |
| PID_LYSOPHOSPHOLIPID_PATHWAY | 65 | -0.6269 | -1.9540 | 0.0000 | 0.0018 |
| PID_A6B1_A6B4_INTEGRIN_PATHWAY | 46 | -0.6489 | -1.9517 | 0.0020 | 0.0018 |
| PID_RAC1_PATHWAY | 54 | -0.6884 | -1.9481 | 0.0000 | 0.0019 |
| PID_TRKR_PATHWAY | 61 | -0.6365 | -1.9416 | 0.0000 | 0.0021 |
| PID_FAK_PATHWAY | 59 | -0.7023 | -1.9339 | 0.0000 | 0.0023 |
| PID_TAP63_PATHWAY | 54 | -0.6095 | -1.9312 | 0.0000 | 0.0025 |
| PID_PLK1_PATHWAY | 46 | -0.7602 | -1.9289 | 0.0019 | 0.0025 |
| PID_FOXM1_PATHWAY | 40 | -0.7040 | -1.9176 | 0.0000 | 0.0027 |
| PID_ARF6_DOWNSTREAM_PATHWAY | 15 | -0.7587 | -1.9176 | 0.0000 | 0.0026 |
| PID_ATR_PATHWAY | 39 | -0.7745 | -1.9040 | 0.0019 | 0.0029 |
| PID_P53_DOWNSTREAM_PATHWAY | 137 | -0.5528 | -1.9004 | 0.0000 | 0.0029 |
| PID_HIF2PATHWAY | 34 | -0.6170 | -1.8988 | 0.0000 | 0.0029 |
| PID_ARF6_TRAFFICKING_PATHWAY | 49 | -0.6538 | -1.8914 | 0.0000 | 0.0032 |
| PID_MAPK_TRK_PATHWAY | 34 | -0.6834 | -1.8910 | 0.0000 | 0.0031 |
| PID_INTEGRIN_A9B1_PATHWAY | 25 | -0.6694 | -1.8906 | 0.0020 | 0.0030 |
| PID_EPHA2_FWD_PATHWAY | 19 | -0.7336 | -1.8889 | 0.0000 | 0.0030 |
| PID_DELTA_NP63_PATHWAY | 47 | -0.5933 | -1.8853 | 0.0000 | 0.0031 |
| PID_RHOA_PATHWAY | 44 | -0.6437 | -1.8846 | 0.0000 | 0.0031 |
| PID_BARD1_PATHWAY | 29 | -0.7799 | -1.8843 | 0.0000 | 0.0030 |
| PID_ERBB1_INTERNALIZATION_PATHWAY | 41 | -0.6733 | -1.8801 | 0.0019 | 0.0030 |
| PID_RET_PATHWAY | 39 | -0.6464 | -1.8776 | 0.0039 | 0.0030 |
| PID_LIS1_PATHWAY | 28 | -0.6881 | -1.8716 | 0.0000 | 0.0032 |
| PID_NCADHERIN_PATHWAY | 33 | -0.7052 | -1.8701 | 0.0000 | 0.0031 |
| PID_BETA_CATENIN_NUC_PATHWAY | 78 | -0.5775 | -1.8629 | 0.0000 | 0.0034 |
| PID_TCPTP_PATHWAY | 42 | -0.6540 | -1.8570 | 0.0019 | 0.0036 |
| PID_HEDGEHOG_GLI_PATHWAY | 48 | -0.6541 | -1.8566 | 0.0018 | 0.0036 |
| PID_TELOMERASE_PATHWAY | 67 | -0.6501 | -1.8563 | 0.0019 | 0.0035 |
| PID_HDAC_CLASSI_PATHWAY | 66 | -0.6165 | -1.8554 | 0.0019 | 0.0035 |
| PID_TGFBR_PATHWAY | 53 | -0.6583 | -1.8545 | 0.0000 | 0.0035 |
| PID_WNT_SIGNALING_PATHWAY | 28 | -0.6260 | -1.8532 | 0.0000 | 0.0035 |
| PID_ECADHERIN_STABILIZATION_PATHWAY | 41 | -0.6584 | -1.8464 | 0.0019 | 0.0036 |
| PID_LKB1_PATHWAY | 47 | -0.6270 | -1.8431 | 0.0019 | 0.0038 |
| PID_MET_PATHWAY | 79 | -0.6243 | -1.8351 | 0.0020 | 0.0041 |
| PID_ECADHERIN_NASCENT_AJ_PATHWAY | 39 | -0.6604 | -1.8319 | 0.0000 | 0.0043 |
| PID_RHOA_REG_PATHWAY | 46 | -0.6687 | -1.8274 | 0.0000 | 0.0045 |
| PID_E2F_PATHWAY | 73 | -0.6445 | -1.8264 | 0.0079 | 0.0045 |
| PID_HIF1_TFPATHWAY | 66 | -0.5600 | -1.8254 | 0.0020 | 0.0045 |
| PID_SMAD2_3NUCLEAR_PATHWAY | 82 | -0.6022 | -1.8247 | 0.0000 | 0.0044 |
| PID_ATM_PATHWAY | 34 | -0.7296 | -1.8232 | 0.0055 | 0.0044 |
| PID_S1P_S1P1_PATHWAY | 21 | -0.6937 | -1.8230 | 0.0019 | 0.0043 |
| PID_ER_NONGENOMIC_PATHWAY | 40 | -0.6297 | -1.8220 | 0.0019 | 0.0042 |
| PID_SYNDECAN_2_PATHWAY | 33 | -0.6336 | -1.8219 | 0.0019 | 0.0041 |
| PID_REELIN_PATHWAY | 28 | -0.6653 | -1.8204 | 0.0038 | 0.0042 |
| PID_ERBB2_ERBB3_PATHWAY | 44 | -0.6165 | -1.8164 | 0.0000 | 0.0043 |
| PID_ERBB1_RECEPTOR_PROXIMAL_PATHWAY | 35 | -0.6775 | -1.8157 | 0.0038 | 0.0043 |
| PID_INSULIN_PATHWAY | 44 | -0.6432 | -1.8129 | 0.0000 | 0.0044 |
| PID_IGF1_PATHWAY | 29 | -0.6533 | -1.8110 | 0.0019 | 0.0044 |
| PID_AR_PATHWAY | 61 | -0.6207 | -1.8106 | 0.0000 | 0.0044 |
| PID_CASPASE_PATHWAY | 51 | -0.6312 | -1.8095 | 0.0000 | 0.0044 |
| PID_RB_1PATHWAY | 65 | -0.5913 | -1.8070 | 0.0000 | 0.0044 |
| PID_TNF_PATHWAY | 46 | -0.6356 | -1.7977 | 0.0038 | 0.0050 |
| PID_P38_ALPHA_BETA_DOWNSTREAM_PATHWAY | 38 | -0.6037 | -1.7943 | 0.0019 | 0.0052 |
| PID_WNT_NONCANONICAL_PATHWAY | 32 | -0.6663 | -1.7927 | 0.0058 | 0.0053 |
| PID_CMYB_PATHWAY | 84 | -0.5534 | -1.7886 | 0.0020 | 0.0055 |
| PID_WNT_CANONICAL_PATHWAY | 20 | -0.6885 | -1.7881 | 0.0019 | 0.0055 |
| PID_P73PATHWAY | 79 | -0.5477 | -1.7873 | 0.0040 | 0.0055 |
| PID_TRAIL_PATHWAY | 28 | -0.6430 | -1.7775 | 0.0093 | 0.0063 |
| PID_PI3KCI_AKT_PATHWAY | 35 | -0.6023 | -1.7719 | 0.0057 | 0.0066 |
| PID_PI3K_PLC_TRK_PATHWAY | 36 | -0.6216 | -1.7718 | 0.0038 | 0.0065 |
| PID_RAC1_REG_PATHWAY | 38 | -0.6389 | -1.7711 | 0.0077 | 0.0065 |
| PID_AURORA_B_PATHWAY | 39 | -0.6952 | -1.7679 | 0.0098 | 0.0068 |
| PID_P38_MK2_PATHWAY | 21 | -0.7010 | -1.7657 | 0.0019 | 0.0070 |
| PID_FGF_PATHWAY | 55 | -0.5628 | -1.7599 | 0.0059 | 0.0075 |
| PID_CDC42_REG_PATHWAY | 30 | -0.6688 | -1.7589 | 0.0057 | 0.0076 |
| PID_CXCR4_PATHWAY | 101 | -0.6077 | -1.7562 | 0.0038 | 0.0078 |
| PID_GMCSF_PATHWAY | 36 | -0.6311 | -1.7544 | 0.0058 | 0.0079 |
| PID_MYC_PATHWAY | 25 | -0.6870 | -1.7506 | 0.0020 | 0.0084 |
| PID_CXCR3_PATHWAY | 43 | -0.5877 | -1.7470 | 0.0115 | 0.0087 |
| PID_RAS_PATHWAY | 30 | -0.6126 | -1.7446 | 0.0038 | 0.0088 |
| PID_PI3KCI_PATHWAY | 48 | -0.6553 | -1.7417 | 0.0019 | 0.0090 |
| PID_NECTIN_PATHWAY | 30 | -0.6706 | -1.7400 | 0.0019 | 0.0091 |
| PID_PTP1B_PATHWAY | 52 | -0.5854 | -1.7385 | 0.0038 | 0.0092 |
| PID_THROMBIN_PAR1_PATHWAY | 43 | -0.5966 | -1.7287 | 0.0117 | 0.0103 |
| PID_KIT_PATHWAY | 52 | -0.6074 | -1.7277 | 0.0076 | 0.0103 |
| PID_EPHA_FWDPATHWAY | 34 | -0.6077 | -1.7243 | 0.0155 | 0.0105 |
| PID_ERB_GENOMIC_PATHWAY | 15 | -0.6514 | -1.7235 | 0.0056 | 0.0105 |
| PID_NFAT_3PATHWAY | 53 | -0.6054 | -1.7148 | 0.0114 | 0.0114 |
| PID_CIRCADIAN_PATHWAY | 16 | -0.6909 | -1.7128 | 0.0098 | 0.0116 |
| PID_IL8_CXCR2_PATHWAY | 34 | -0.6540 | -1.7096 | 0.0131 | 0.0123 |
| PID_P53_REGULATION_PATHWAY | 59 | -0.6138 | -1.7070 | 0.0077 | 0.0125 |
| PID_MYC_REPRESS_PATHWAY | 63 | -0.5245 | -1.7054 | 0.0038 | 0.0126 |
| PID_AURORA_A_PATHWAY | 31 | -0.6267 | -1.6996 | 0.0198 | 0.0132 |
| PID_AJDISS_2PATHWAY | 48 | -0.5737 | -1.6990 | 0.0096 | 0.0131 |
| PID_FANCONI_PATHWAY | 47 | -0.6910 | -1.6962 | 0.0211 | 0.0134 |
| PID_IL2_PI3K_PATHWAY | 34 | -0.6025 | -1.6935 | 0.0153 | 0.0136 |
| PID_PDGFRA_PATHWAY | 22 | -0.6400 | -1.6931 | 0.0041 | 0.0135 |
| PID_ALK1_PATHWAY | 26 | -0.6354 | -1.6896 | 0.0115 | 0.0139 |
| PID_P75_NTR_PATHWAY | 68 | -0.5324 | -1.6889 | 0.0038 | 0.0138 |
| PID_AVB3_INTEGRIN_PATHWAY | 74 | -0.5768 | -1.6887 | 0.0263 | 0.0137 |
| PID_FAS_PATHWAY | 38 | -0.6123 | -1.6884 | 0.0151 | 0.0136 |
| PID_BMP_PATHWAY | 42 | -0.5620 | -1.6828 | 0.0096 | 0.0144 |
| PID_FCER1_PATHWAY | 60 | -0.5981 | -1.6827 | 0.0133 | 0.0142 |
| PID_INTEGRIN_A4B1_PATHWAY | 33 | -0.5835 | -1.6826 | 0.0226 | 0.0141 |
| PID_S1P_S1P2_PATHWAY | 24 | -0.6273 | -1.6793 | 0.0121 | 0.0144 |
| PID_ERBB4_PATHWAY | 38 | -0.5552 | -1.6767 | 0.0154 | 0.0146 |
| PID_ALPHA_SYNUCLEIN_PATHWAY | 32 | -0.5961 | -1.6766 | 0.0075 | 0.0145 |
| PID_IL3_PATHWAY | 26 | -0.5780 | -1.6760 | 0.0152 | 0.0144 |
| PID_EPO_PATHWAY | 33 | -0.6111 | -1.6731 | 0.0099 | 0.0147 |
| PID_CERAMIDE_PATHWAY | 44 | -0.5681 | -1.6711 | 0.0135 | 0.0149 |
| PID_AVB3_OPN_PATHWAY | 31 | -0.6299 | -1.6709 | 0.0193 | 0.0148 |
| PID_ARF6_PATHWAY | 35 | -0.5665 | -1.6663 | 0.0174 | 0.0153 |
| PID_ENDOTHELIN_PATHWAY | 63 | -0.5506 | -1.6657 | 0.0235 | 0.0153 |
| PID_PS1_PATHWAY | 45 | -0.5668 | -1.6619 | 0.0247 | 0.0158 |
| PID_NEPHRIN_NEPH1_PATHWAY | 31 | -0.5941 | -1.6578 | 0.0079 | 0.0164 |
| PID_IL1_PATHWAY | 34 | -0.6031 | -1.6568 | 0.0176 | 0.0165 |
| PID_HES_HEY_PATHWAY | 48 | -0.5533 | -1.6497 | 0.0274 | 0.0173 |
| PID_ECADHERIN_KERATINOCYTE_PATHWAY | 21 | -0.6447 | -1.6411 | 0.0117 | 0.0186 |
| PID_SYNDECAN_3_PATHWAY | 17 | -0.6418 | -1.6398 | 0.0188 | 0.0187 |
| PID_BETA_CATENIN_DEG_PATHWAY | 18 | -0.6485 | -1.6386 | 0.0266 | 0.0189 |
| PID_DNA_PK_PATHWAY | 16 | -0.6695 | -1.6300 | 0.0171 | 0.0203 |
| PID_SMAD2_3PATHWAY | 16 | -0.7290 | -1.6247 | 0.0114 | 0.0212 |
| PID_GLYPICAN_1PATHWAY | 27 | -0.5930 | -1.6234 | 0.0343 | 0.0213 |
| PID_IL2_1PATHWAY | 55 | -0.5702 | -1.6220 | 0.0196 | 0.0214 |
| PID_REG_GR_PATHWAY | 82 | -0.4863 | -1.6197 | 0.0174 | 0.0218 |
| PID_P38_ALPHA_BETA_PATHWAY | 30 | -0.5723 | -1.6180 | 0.0286 | 0.0220 |
| PID_SYNDECAN_4_PATHWAY | 32 | -0.5311 | -1.6174 | 0.0237 | 0.0219 |
| PID_S1P_S1P3_PATHWAY | 29 | -0.6082 | -1.6146 | 0.0374 | 0.0223 |
| PID_VEGFR1_PATHWAY | 26 | -0.6187 | -1.6136 | 0.0328 | 0.0223 |
| PID_S1P_META_PATHWAY | 21 | -0.5984 | -1.6095 | 0.0292 | 0.0229 |
| PID_AR_TF_PATHWAY | 53 | -0.5406 | -1.6083 | 0.0270 | 0.0230 |
| PID_FOXO_PATHWAY | 49 | -0.5535 | -1.6076 | 0.0391 | 0.0230 |
| PID_IL8_CXCR1_PATHWAY | 28 | -0.6225 | -1.6075 | 0.0478 | 0.0228 |
| PID_IFNG_PATHWAY | 40 | -0.5853 | -1.6010 | 0.0441 | 0.0240 |
| PID_ERA_GENOMIC_PATHWAY | 64 | -0.4678 | -1.5832 | 0.0119 | 0.0281 |
| PID_INTEGRIN_CS_PATHWAY | 26 | -0.6217 | -1.5788 | 0.0362 | 0.0288 |
| PID_RXR_VDR_PATHWAY | 26 | -0.5406 | -1.5751 | 0.0356 | 0.0293 |
| PID_BCR_5PATHWAY | 63 | -0.5740 | -1.5675 | 0.0472 | 0.0308 |
| PID_TXA2PATHWAY | 56 | -0.5687 | -1.5650 | 0.0657 | 0.0315 |
| PID_NFKAPPAB_CANONICAL_PATHWAY | 23 | -0.6221 | -1.5646 | 0.0451 | 0.0313 |
| PID_HIV_NEF_PATHWAY | 35 | -0.5715 | -1.5571 | 0.0284 | 0.0327 |
| PID_NFKAPPAB_ATYPICAL_PATHWAY | 17 | -0.6264 | -1.5534 | 0.0307 | 0.0335 |
| PID_LYMPH_ANGIOGENESIS_PATHWAY | 25 | -0.5761 | -1.5373 | 0.0592 | 0.0376 |
| PID_FRA_PATHWAY | 37 | -0.5658 | -1.5348 | 0.0615 | 0.0381 |
| PID_RETINOIC_ACID_PATHWAY | 30 | -0.5319 | -1.5018 | 0.0673 | 0.0481 |
| PID_ANGIOPOIETIN_RECEPTOR_PATHWAY | 49 | -0.5067 | -1.5006 | 0.0730 | 0.0482 |
| PID_AP1_PATHWAY | 69 | -0.4826 | -1.4986 | 0.0579 | 0.0486 |
| PID_SYNDECAN_1_PATHWAY | 46 | -0.5495 | -1.4926 | 0.0814 | 0.0505 |
| PID_CD40_PATHWAY | 31 | -0.5455 | -1.4906 | 0.0599 | 0.0508 |
| PID_ATF2_PATHWAY | 59 | -0.4546 | -1.4848 | 0.0628 | 0.0522 |
| PID_EPHRINB_REV_PATHWAY | 30 | -0.5361 | -1.4786 | 0.0621 | 0.0540 |
| PID_IL2_STAT5_PATHWAY | 30 | -0.5749 | -1.4591 | 0.0940 | 0.0612 |
| PID_SHP2_PATHWAY | 57 | -0.4841 | -1.4476 | 0.0727 | 0.0652 |
| PID_UPA_UPAR_PATHWAY | 42 | -0.4843 | -1.4246 | 0.1021 | 0.0740 |
| PID_TOLL_ENDOGENOUS_PATHWAY | 24 | -0.5077 | -1.4111 | 0.0933 | 0.0791 |
| PID_P38_MKK3_6PATHWAY | 26 | -0.4819 | -1.4099 | 0.0866 | 0.0790 |
| PID_HDAC_CLASSIII_PATHWAY | 26 | -0.4651 | -1.3975 | 0.1074 | 0.0839 |
| PID_ANTHRAX_PATHWAY | 18 | -0.5412 | -1.3869 | 0.1180 | 0.0888 |
| PID_LPA4_PATHWAY | 15 | -0.5447 | -1.3840 | 0.1192 | 0.0897 |
| PID_TCR_PATHWAY | 65 | -0.5515 | -1.3819 | 0.1708 | 0.0901 |
| PID_IL4_2PATHWAY | 64 | -0.4550 | -1.3703 | 0.1242 | 0.0951 |
| PID_INTEGRIN3_PATHWAY | 42 | -0.4805 | -1.3652 | 0.1642 | 0.0965 |
| PID_THROMBIN_PAR4_PATHWAY | 15 | -0.5644 | -1.3580 | 0.1547 | 0.0994 |
| PID_CD8_TCR_PATHWAY | 53 | -0.5496 | -1.3534 | 0.1806 | 0.1008 |
| PID_TCR_CALCIUM_PATHWAY | 28 | -0.5122 | -1.3423 | 0.1562 | 0.1055 |
| PID_INTEGRIN1_PATHWAY | 66 | -0.4544 | -1.3373 | 0.1754 | 0.1073 |
| PID_HEDGEHOG_2PATHWAY | 22 | -0.4747 | -1.3237 | 0.1036 | 0.1134 |
| PID_ERBB_NETWORK_PATHWAY | 15 | -0.5375 | -1.3132 | 0.1831 | 0.1183 |
| PID_IL6_7_PATHWAY | 47 | -0.4413 | -1.2996 | 0.1721 | 0.1254 |
| PID_CD8_TCR_DOWNSTREAM_PATHWAY | 65 | -0.4523 | -1.2944 | 0.2233 | 0.1275 |
| PID_IL27_PATHWAY | 26 | -0.5100 | -1.2822 | 0.2331 | 0.1337 |
| PID_CONE_PATHWAY | 22 | -0.4383 | -1.2781 | 0.1449 | 0.1351 |
| PID_AMB2_NEUTROPHILS_PATHWAY | 40 | -0.4579 | -1.2551 | 0.2249 | 0.1484 |
| PID_NFAT_TFPATHWAY | 45 | -0.4258 | -1.1790 | 0.3008 | 0.1977 |
| PID_INTEGRIN5_PATHWAY | 16 | -0.4666 | -1.1695 | 0.2954 | 0.2031 |
| PID_RHODOPSIN_PATHWAY | 23 | -0.3803 | -1.1453 | 0.2738 | 0.2200 |
| PID_IL23_PATHWAY | 37 | -0.4158 | -1.1023 | 0.3762 | 0.2537 |
| PID_IL12_2PATHWAY | 63 | -0.4156 | -1.0662 | 0.4229 | 0.2816 |
| PID_IL12_STAT4_PATHWAY | 33 | -0.4464 | -1.0642 | 0.4405 | 0.2816 |

| **Signaling pathways enriched in HCC samples corresponding ADH1B expression by GSEA based on PID** | | | | | |
| --- | --- | --- | --- | --- | --- |
| **PID Name** | **Size** | **ES** | **NES** | **NOM p-value** | **FDR q-value** |
| PID_HNF3B_PATHWAY | 45 | 0.4766 | 1.5607 | 0.0198 | 0.0494 |
| PID_HNF3A_PATHWAY | 44 | 0.4335 | 1.4393 | 0.0529 | 0.0583 |
| PID_HIF1A_PATHWAY | 19 | -0.7507 | -2.0110 | 0.0000 | 0.0161 |
| PID_RAC1_PATHWAY | 54 | -0.7023 | -1.9922 | 0.0000 | 0.0091 |
| PID_ATR_PATHWAY | 39 | -0.8020 | -1.9526 | 0.0000 | 0.0141 |
| PID_EPHA2_FWD_PATHWAY | 19 | -0.7390 | -1.9426 | 0.0021 | 0.0111 |
| PID_ATM_PATHWAY | 34 | -0.7830 | -1.9404 | 0.0000 | 0.0088 |
| PID_INSULIN_GLUCOSE_PATHWAY | 26 | -0.6836 | -1.9310 | 0.0021 | 0.0089 |
| PID_MYC_ACTIV_PATHWAY | 79 | -0.6578 | -1.9302 | 0.0000 | 0.0076 |
| PID_P73PATHWAY | 79 | -0.5831 | -1.9193 | 0.0040 | 0.0082 |
| PID_ARF6_DOWNSTREAM_PATHWAY | 15 | -0.7844 | -1.9180 | 0.0000 | 0.0074 |
| PID_PLK1_PATHWAY | 46 | -0.7675 | -1.8767 | 0.0021 | 0.0132 |
| PID_ERB_GENOMIC_PATHWAY | 15 | -0.6832 | -1.8708 | 0.0020 | 0.0138 |
| PID_ERBB1_DOWNSTREAM_PATHWAY | 105 | -0.6115 | -1.8669 | 0.0040 | 0.0134 |
| PID_ILK_PATHWAY | 45 | -0.6745 | -1.8586 | 0.0000 | 0.0142 |
| PID_MYC_PATHWAY | 25 | -0.7219 | -1.8548 | 0.0000 | 0.0136 |
| PID_FOXM1_PATHWAY | 40 | -0.7100 | -1.8519 | 0.0000 | 0.0134 |
| PID_LKB1_PATHWAY | 47 | -0.6257 | -1.8491 | 0.0000 | 0.0134 |
| PID_LIS1_PATHWAY | 28 | -0.6742 | -1.8462 | 0.0000 | 0.0131 |
| PID_CDC42_PATHWAY | 70 | -0.6135 | -1.8435 | 0.0079 | 0.0129 |
| PID_CASPASE_PATHWAY | 51 | -0.6345 | -1.8417 | 0.0021 | 0.0125 |
| PID_ARF_3PATHWAY | 19 | -0.7411 | -1.8345 | 0.0000 | 0.0136 |
| PID_NOTCH_PATHWAY | 59 | -0.6275 | -1.8338 | 0.0021 | 0.0131 |
| PID_BARD1_PATHWAY | 29 | -0.7803 | -1.8331 | 0.0000 | 0.0125 |
| PID_RHOA_REG_PATHWAY | 46 | -0.6529 | -1.8228 | 0.0000 | 0.0142 |
| PID_DNA_PK_PATHWAY | 16 | -0.7388 | -1.8189 | 0.0021 | 0.0143 |
| PID_PRL_SIGNALING_EVENTS_PATHWAY | 23 | -0.6435 | -1.8177 | 0.0063 | 0.0140 |
| PID_DELTA_NP63_PATHWAY | 47 | -0.5747 | -1.8158 | 0.0000 | 0.0135 |
| PID_WNT_NONCANONICAL_PATHWAY | 32 | -0.6793 | -1.8082 | 0.0062 | 0.0145 |
| PID_PDGFRB_PATHWAY | 129 | -0.6065 | -1.8061 | 0.0060 | 0.0143 |
| PID_EPHB_FWD_PATHWAY | 40 | -0.6443 | -1.7914 | 0.0062 | 0.0165 |
| PID_ECADHERIN_STABILIZATION_PATHWAY | 41 | -0.6487 | -1.7854 | 0.0041 | 0.0170 |
| PID_P38_MK2_PATHWAY | 21 | -0.7095 | -1.7835 | 0.0020 | 0.0168 |
| PID_RB_1PATHWAY | 65 | -0.6030 | -1.7806 | 0.0021 | 0.0167 |
| PID_A6B1_A6B4_INTEGRIN_PATHWAY | 46 | -0.5925 | -1.7724 | 0.0063 | 0.0184 |
| PID_TCPTP_PATHWAY | 42 | -0.6270 | -1.7643 | 0.0122 | 0.0195 |
| PID_TELOMERASE_PATHWAY | 67 | -0.6182 | -1.7613 | 0.0063 | 0.0196 |
| PID_FANCONI_PATHWAY | 47 | -0.7185 | -1.7449 | 0.0083 | 0.0238 |
| PID_P53_DOWNSTREAM_PATHWAY | 137 | -0.5150 | -1.7408 | 0.0102 | 0.0241 |
| PID_WNT_SIGNALING_PATHWAY | 28 | -0.6011 | -1.7397 | 0.0021 | 0.0237 |
| PID_RHOA_PATHWAY | 44 | -0.6115 | -1.7351 | 0.0124 | 0.0247 |
| PID_PS1_PATHWAY | 45 | -0.5832 | -1.7284 | 0.0128 | 0.0259 |
| PID_HDAC_CLASSII_PATHWAY | 34 | -0.6139 | -1.7272 | 0.0063 | 0.0258 |
| PID_HDAC_CLASSI_PATHWAY | 66 | -0.5658 | -1.7258 | 0.0102 | 0.0255 |
| PID_TAP63_PATHWAY | 54 | -0.5387 | -1.7189 | 0.0041 | 0.0270 |
| PID_AJDISS_2PATHWAY | 48 | -0.5768 | -1.7151 | 0.0164 | 0.0275 |
| PID_BETA_CATENIN_DEG_PATHWAY | 18 | -0.6706 | -1.7133 | 0.0260 | 0.0274 |
| PID_MTOR_4PATHWAY | 69 | -0.5750 | -1.7085 | 0.0084 | 0.0281 |
| PID_TRKR_PATHWAY | 61 | -0.5696 | -1.7048 | 0.0158 | 0.0284 |
| PID_BETA_CATENIN_NUC_PATHWAY | 78 | -0.5232 | -1.7022 | 0.0106 | 0.0287 |
| PID_NECTIN_PATHWAY | 30 | -0.6618 | -1.6986 | 0.0265 | 0.0289 |
| PID_NFAT_3PATHWAY | 53 | -0.5963 | -1.6980 | 0.0205 | 0.0285 |
| PID_P75_NTR_PATHWAY | 68 | -0.5310 | -1.6886 | 0.0082 | 0.0306 |
| PID_MET_PATHWAY | 79 | -0.5811 | -1.6866 | 0.0226 | 0.0307 |
| PID_ECADHERIN_NASCENT_AJ_PATHWAY | 39 | -0.6108 | -1.6835 | 0.0325 | 0.0310 |
| PID_P53_REGULATION_PATHWAY | 59 | -0.6032 | -1.6807 | 0.0206 | 0.0313 |
| PID_AURORA_A_PATHWAY | 31 | -0.6335 | -1.6791 | 0.0148 | 0.0313 |
| PID_P38_ALPHA_BETA_DOWNSTREAM_PATHWAY | 38 | -0.5574 | -1.6716 | 0.0122 | 0.0330 |
| PID_SYNDECAN_2_PATHWAY | 33 | -0.5621 | -1.6715 | 0.0222 | 0.0325 |
| PID_NCADHERIN_PATHWAY | 33 | -0.6171 | -1.6689 | 0.0276 | 0.0327 |
| PID_ERBB2_ERBB3_PATHWAY | 44 | -0.5679 | -1.6662 | 0.0205 | 0.0331 |
| PID_PI3KCI_PATHWAY | 48 | -0.6297 | -1.6636 | 0.0122 | 0.0334 |
| PID_ERBB1_RECEPTOR_PROXIMAL_PATHWAY | 35 | -0.6234 | -1.6563 | 0.0286 | 0.0350 |
| PID_IL8_CXCR2_PATHWAY | 34 | -0.6489 | -1.6546 | 0.0221 | 0.0351 |
| PID_ALK1_PATHWAY | 26 | -0.6243 | -1.6528 | 0.0185 | 0.0350 |
| PID_RAS_PATHWAY | 30 | -0.5830 | -1.6491 | 0.0162 | 0.0355 |
| PID_CERAMIDE_PATHWAY | 44 | -0.5629 | -1.6487 | 0.0171 | 0.0350 |
| PID_VEGFR1_2_PATHWAY | 69 | -0.5670 | -1.6466 | 0.0369 | 0.0351 |
| PID_FAK_PATHWAY | 59 | -0.6035 | -1.6412 | 0.0507 | 0.0361 |
| PID_CIRCADIAN_PATHWAY | 16 | -0.6537 | -1.6389 | 0.0262 | 0.0363 |
| PID_AURORA_B_PATHWAY | 39 | -0.6559 | -1.6297 | 0.0288 | 0.0390 |
| PID_PI3K_PLC_TRK_PATHWAY | 36 | -0.5745 | -1.6251 | 0.0271 | 0.0400 |
| PID_WNT_CANONICAL_PATHWAY | 20 | -0.6283 | -1.6202 | 0.0297 | 0.0414 |
| PID_MYC_REPRESS_PATHWAY | 63 | -0.4933 | -1.6201 | 0.0252 | 0.0408 |
| PID_CXCR4_PATHWAY | 101 | -0.5546 | -1.6177 | 0.0448 | 0.0410 |
| PID_ERBB1_INTERNALIZATION_PATHWAY | 41 | -0.5856 | -1.6074 | 0.0431 | 0.0449 |
| PID_CXCR3_PATHWAY | 43 | -0.5354 | -1.5937 | 0.0405 | 0.0503 |
| PID_ALPHA_SYNUCLEIN_PATHWAY | 32 | -0.5670 | -1.5895 | 0.0249 | 0.0511 |
| PID_TNF_PATHWAY | 46 | -0.5580 | -1.5828 | 0.0376 | 0.0529 |
| PID_CMYB_PATHWAY | 84 | -0.4809 | -1.5803 | 0.0417 | 0.0534 |
| PID_IL8_CXCR1_PATHWAY | 28 | -0.6241 | -1.5796 | 0.0559 | 0.0529 |
| PID_AR_PATHWAY | 61 | -0.5490 | -1.5735 | 0.0484 | 0.0549 |
| PID_MAPK_TRK_PATHWAY | 34 | -0.5694 | -1.5731 | 0.0471 | 0.0544 |
| PID_AR_NONGENOMIC_PATHWAY | 31 | -0.5335 | -1.5711 | 0.0482 | 0.0544 |
| PID_INSULIN_PATHWAY | 44 | -0.5524 | -1.5692 | 0.0485 | 0.0543 |
| PID_SMAD2_3PATHWAY | 16 | -0.6902 | -1.5622 | 0.0297 | 0.0562 |
| PID_S1P_S1P1_PATHWAY | 21 | -0.5954 | -1.5594 | 0.0475 | 0.0566 |
| PID_TGFBR_PATHWAY | 53 | -0.5402 | -1.5561 | 0.0607 | 0.0573 |
| PID_CDC42_REG_PATHWAY | 30 | -0.5910 | -1.5464 | 0.0552 | 0.0609 |
| PID_E2F_PATHWAY | 73 | -0.5534 | -1.5419 | 0.0669 | 0.0621 |
| PID_GMCSF_PATHWAY | 36 | -0.5576 | -1.5417 | 0.0636 | 0.0615 |
| PID_KIT_PATHWAY | 52 | -0.5408 | -1.5415 | 0.0728 | 0.0608 |
| PID_THROMBIN_PAR1_PATHWAY | 43 | -0.5282 | -1.5371 | 0.0465 | 0.0624 |
| PID_ECADHERIN_KERATINOCYTE_PATHWAY | 21 | -0.5988 | -1.5304 | 0.0521 | 0.0651 |
| PID_IGF1_PATHWAY | 29 | -0.5480 | -1.5275 | 0.0823 | 0.0656 |
| PID_NETRIN_PATHWAY | 32 | -0.5626 | -1.5270 | 0.0624 | 0.0651 |
| PID_ARF6_TRAFFICKING_PATHWAY | 49 | -0.5180 | -1.5173 | 0.0815 | 0.0693 |
| PID_FGF_PATHWAY | 55 | -0.4903 | -1.5132 | 0.0460 | 0.0705 |
| PID_ANTHRAX_PATHWAY | 18 | -0.5939 | -1.5131 | 0.0607 | 0.0699 |
| PID_SYNDECAN_4_PATHWAY | 32 | -0.4946 | -1.5028 | 0.0565 | 0.0742 |
| PID_PI3KCI_AKT_PATHWAY | 35 | -0.5194 | -1.5009 | 0.0655 | 0.0743 |
| PID_RET_PATHWAY | 39 | -0.5232 | -1.4991 | 0.0731 | 0.0745 |
| PID_S1P_S1P3_PATHWAY | 29 | -0.5633 | -1.4951 | 0.0895 | 0.0756 |
| PID_FOXO_PATHWAY | 49 | -0.5141 | -1.4934 | 0.0815 | 0.0755 |
| PID_REG_GR_PATHWAY | 82 | -0.4485 | -1.4933 | 0.0350 | 0.0748 |
| PID_SYNDECAN_1_PATHWAY | 46 | -0.5332 | -1.4933 | 0.0841 | 0.0741 |
| PID_INTEGRIN_A4B1_PATHWAY | 33 | -0.5213 | -1.4897 | 0.0816 | 0.0752 |
| PID_ENDOTHELIN_PATHWAY | 63 | -0.5001 | -1.4891 | 0.0766 | 0.0752 |
| PID_HIF2PATHWAY | 34 | -0.4939 | -1.4858 | 0.0583 | 0.0762 |
| PID_REELIN_PATHWAY | 28 | -0.5472 | -1.4831 | 0.0842 | 0.0768 |
| PID_FCER1_PATHWAY | 60 | -0.5201 | -1.4766 | 0.0977 | 0.0790 |
| PID_SYNDECAN_3_PATHWAY | 17 | -0.5712 | -1.4753 | 0.0665 | 0.0794 |
| PID_TXA2PATHWAY | 56 | -0.5306 | -1.4728 | 0.0972 | 0.0798 |
| PID_LYSOPHOSPHOLIPID_PATHWAY | 65 | -0.4820 | -1.4693 | 0.1030 | 0.0819 |
| PID_FRA_PATHWAY | 37 | -0.5405 | -1.4690 | 0.0924 | 0.0813 |
| PID_EPHA_FWDPATHWAY | 34 | -0.5285 | -1.4600 | 0.0553 | 0.0855 |
| PID_SMAD2_3NUCLEAR_PATHWAY | 82 | -0.4953 | -1.4592 | 0.0861 | 0.0851 |
| PID_CD8_TCR_DOWNSTREAM_PATHWAY | 65 | -0.5006 | -1.4579 | 0.1081 | 0.0850 |
| PID_IFNG_PATHWAY | 40 | -0.5298 | -1.4538 | 0.0953 | 0.0866 |
| PID_RAC1_REG_PATHWAY | 38 | -0.5265 | -1.4537 | 0.1002 | 0.0859 |
| PID_HEDGEHOG_GLI_PATHWAY | 48 | -0.5193 | -1.4520 | 0.0964 | 0.0859 |
| PID_NEPHRIN_NEPH1_PATHWAY | 31 | -0.5177 | -1.4471 | 0.0880 | 0.0871 |
| PID_ER_NONGENOMIC_PATHWAY | 40 | -0.5196 | -1.4425 | 0.1125 | 0.0886 |
| PID_ARF6_PATHWAY | 35 | -0.4917 | -1.4398 | 0.0798 | 0.0890 |
| PID_P38_ALPHA_BETA_PATHWAY | 30 | -0.5059 | -1.4257 | 0.1089 | 0.0950 |
| PID_AVB3_OPN_PATHWAY | 31 | -0.5348 | -1.4238 | 0.1006 | 0.0952 |
| PID_FAS_PATHWAY | 38 | -0.5080 | -1.4212 | 0.0872 | 0.0959 |
| PID_AVB3_INTEGRIN_PATHWAY | 74 | -0.4865 | -1.4191 | 0.1260 | 0.0960 |
| PID_TRAIL_PATHWAY | 28 | -0.5180 | -1.4188 | 0.1104 | 0.0953 |
| PID_ERA_GENOMIC_PATHWAY | 64 | -0.4058 | -1.4188 | 0.0693 | 0.0946 |
| PID_INTEGRIN_CS_PATHWAY | 26 | -0.5512 | -1.4186 | 0.1242 | 0.0939 |
| PID_TCR_PATHWAY | 65 | -0.5545 | -1.4066 | 0.1508 | 0.0999 |
| PID_HIV_NEF_PATHWAY | 35 | -0.4982 | -1.3970 | 0.1495 | 0.1043 |
| PID_BCR_5PATHWAY | 63 | -0.5100 | -1.3961 | 0.1374 | 0.1039 |
| PID_PTP1B_PATHWAY | 52 | -0.4628 | -1.3848 | 0.1300 | 0.1090 |
| PID_INTEGRIN_A9B1_PATHWAY | 25 | -0.4937 | -1.3818 | 0.1366 | 0.1098 |
| PID_VEGFR1_PATHWAY | 26 | -0.5293 | -1.3793 | 0.1449 | 0.1109 |
| PID_NFKAPPAB_ATYPICAL_PATHWAY | 17 | -0.5405 | -1.3712 | 0.1201 | 0.1138 |
| PID_CD8_TCR_PATHWAY | 53 | -0.5391 | -1.3513 | 0.1794 | 0.1244 |
| PID_HES_HEY_PATHWAY | 48 | -0.4617 | -1.3509 | 0.1579 | 0.1237 |
| PID_AR_TF_PATHWAY | 53 | -0.4454 | -1.3349 | 0.1791 | 0.1321 |
| PID_EPHRINB_REV_PATHWAY | 30 | -0.4776 | -1.3326 | 0.1676 | 0.1325 |
| PID_TOLL_ENDOGENOUS_PATHWAY | 24 | -0.4745 | -1.3321 | 0.1400 | 0.1319 |
| PID_IL2_PI3K_PATHWAY | 34 | -0.4813 | -1.3309 | 0.1746 | 0.1317 |
| PID_ERBB_NETWORK_PATHWAY | 15 | -0.5490 | -1.3280 | 0.1523 | 0.1324 |
| PID_AP1_PATHWAY | 69 | -0.4184 | -1.3084 | 0.1554 | 0.1431 |
| PID_NFKAPPAB_CANONICAL_PATHWAY | 23 | -0.5248 | -1.2900 | 0.2054 | 0.1541 |
| PID_RXR_VDR_PATHWAY | 26 | -0.4413 | -1.2692 | 0.2042 | 0.1670 |
| PID_PDGFRA_PATHWAY | 22 | -0.4719 | -1.2648 | 0.1996 | 0.1686 |
| PID_LYMPH_ANGIOGENESIS_PATHWAY | 25 | -0.4904 | -1.2625 | 0.2251 | 0.1689 |
| PID_GLYPICAN_1PATHWAY | 27 | -0.4636 | -1.2620 | 0.2085 | 0.1681 |
| PID_HIF1_TFPATHWAY | 66 | -0.3892 | -1.2618 | 0.2000 | 0.1671 |
| PID_IL4_2PATHWAY | 64 | -0.4126 | -1.2592 | 0.2073 | 0.1688 |
| PID_THROMBIN_PAR4_PATHWAY | 15 | -0.5017 | -1.2491 | 0.2224 | 0.1744 |
| PID_INTEGRIN5_PATHWAY | 16 | -0.4873 | -1.2334 | 0.2147 | 0.1833 |
| PID_P38_MKK3_6PATHWAY | 26 | -0.4135 | -1.2318 | 0.2283 | 0.1834 |
| PID_NFAT_TFPATHWAY | 45 | -0.4424 | -1.2302 | 0.2369 | 0.1832 |
| PID_CD40_PATHWAY | 31 | -0.4397 | -1.2280 | 0.2540 | 0.1836 |
| PID_RETINOIC_ACID_PATHWAY | 30 | -0.4384 | -1.2214 | 0.2662 | 0.1879 |
| PID_IL12_STAT4_PATHWAY | 33 | -0.4945 | -1.2181 | 0.2788 | 0.1891 |
| PID_S1P_S1P2_PATHWAY | 24 | -0.4586 | -1.2068 | 0.2790 | 0.1955 |
| PID_UPA_UPAR_PATHWAY | 42 | -0.4149 | -1.1975 | 0.2649 | 0.2014 |
| PID_IL2_1PATHWAY | 55 | -0.4158 | -1.1920 | 0.2933 | 0.2050 |
| PID_S1P_META_PATHWAY | 21 | -0.4461 | -1.1823 | 0.2799 | 0.2106 |
| PID_ANGIOPOIETIN_RECEPTOR_PATHWAY | 49 | -0.3998 | -1.1818 | 0.2813 | 0.2096 |
| PID_IL3_PATHWAY | 26 | -0.4121 | -1.1793 | 0.2714 | 0.2106 |
| PID_TCR_CALCIUM_PATHWAY | 28 | -0.4374 | -1.1684 | 0.3116 | 0.2174 |
| PID_INTEGRIN1_PATHWAY | 66 | -0.3989 | -1.1595 | 0.2976 | 0.2230 |
| PID_ERBB4_PATHWAY | 38 | -0.3853 | -1.1503 | 0.2857 | 0.2282 |
| PID_INTEGRIN3_PATHWAY | 42 | -0.4110 | -1.1441 | 0.2874 | 0.2318 |
| PID_IL27_PATHWAY | 26 | -0.4379 | -1.1387 | 0.3352 | 0.2355 |
| PID_LPA4_PATHWAY | 15 | -0.4553 | -1.1373 | 0.3133 | 0.2351 |
| PID_AMB2_NEUTROPHILS_PATHWAY | 40 | -0.4152 | -1.1325 | 0.3437 | 0.2377 |
| PID_BMP_PATHWAY | 42 | -0.3719 | -1.1223 | 0.3285 | 0.2439 |
| PID_IL2_STAT5_PATHWAY | 30 | -0.4356 | -1.1147 | 0.3843 | 0.2491 |
| PID_CONE_PATHWAY | 22 | -0.3775 | -1.1046 | 0.3195 | 0.2562 |
| PID_EPO_PATHWAY | 33 | -0.4016 | -1.1016 | 0.3618 | 0.2571 |
| PID_ATF2_PATHWAY | 59 | -0.3369 | -1.0977 | 0.3261 | 0.2594 |
| PID_IL1_PATHWAY | 34 | -0.4051 | -1.0905 | 0.3821 | 0.2635 |
| PID_SHP2_PATHWAY | 57 | -0.3542 | -1.0361 | 0.4148 | 0.3080 |
| PID_IL6_7_PATHWAY | 47 | -0.3490 | -1.0331 | 0.4269 | 0.3087 |
| PID_RHODOPSIN_PATHWAY | 23 | -0.3249 | -0.9840 | 0.5010 | 0.3564 |
| PID_IL23_PATHWAY | 37 | -0.3472 | -0.9501 | 0.5165 | 0.3920 |
| PID_HEDGEHOG_2PATHWAY | 22 | -0.3408 | -0.9433 | 0.5229 | 0.3961 |
| PID_IL12_2PATHWAY | 63 | -0.3439 | -0.9247 | 0.5344 | 0.4141 |
| PID_HDAC_CLASSIII_PATHWAY | 26 | -0.3091 | -0.9228 | 0.5288 | 0.4139 |
| PID_INTEGRIN2_PATHWAY | 28 | -0.3319 | -0.8834 | 0.6064 | 0.4587 |

| **Signaling pathways enriched in HCC samples corresponding ADH1C expression by GSEA based on PID** | | | | | |
| --- | --- | --- | --- | --- | --- |
| **PID Name** | **Size** | **ES** | **NES** | **NOM p-value** | **FDR q-value** |
| PID_HNF3B_PATHWAY | 45 | 0.4656 | 1.5340 | 0.0333 | 0.0896 |
| PID_HNF3A_PATHWAY | 44 | 0.3900 | 1.2874 | 0.1300 | 0.1873 |
| PID_INTEGRIN2_PATHWAY | 28 | 0.3137 | 0.8542 | 0.6143 | 0.5056 |
| PID_DNA_PK_PATHWAY | 16 | -0.8355 | -2.0835 | 0.0000 | 0.0000 |
| PID_RAC1_PATHWAY | 54 | -0.7279 | -2.0147 | 0.0000 | 0.0026 |
| PID_MTOR_4PATHWAY | 69 | -0.6557 | -1.9850 | 0.0000 | 0.0035 |
| PID_MYC_PATHWAY | 25 | -0.7556 | -1.9790 | 0.0000 | 0.0037 |
| PID_ILK_PATHWAY | 45 | -0.7110 | -1.9759 | 0.0000 | 0.0029 |
| PID_CDC42_PATHWAY | 70 | -0.6571 | -1.9597 | 0.0000 | 0.0035 |
| PID_INSULIN_GLUCOSE_PATHWAY | 26 | -0.7005 | -1.9579 | 0.0022 | 0.0033 |
| PID_MYC_ACTIV_PATHWAY | 79 | -0.6670 | -1.9549 | 0.0000 | 0.0030 |
| PID_LKB1_PATHWAY | 47 | -0.6638 | -1.9461 | 0.0000 | 0.0030 |
| PID_PRL_SIGNALING_EVENTS_PATHWAY | 23 | -0.6743 | -1.9306 | 0.0000 | 0.0030 |
| PID_PLK1_PATHWAY | 46 | -0.7795 | -1.9288 | 0.0000 | 0.0028 |
| PID_FOXM1_PATHWAY | 40 | -0.7262 | -1.9246 | 0.0000 | 0.0030 |
| PID_ARF_3PATHWAY | 19 | -0.7982 | -1.9237 | 0.0000 | 0.0029 |
| PID_RB_1PATHWAY | 65 | -0.6390 | -1.9190 | 0.0021 | 0.0029 |
| PID_LIS1_PATHWAY | 28 | -0.7106 | -1.9184 | 0.0000 | 0.0027 |
| PID_WNT_NONCANONICAL_PATHWAY | 32 | -0.7065 | -1.9064 | 0.0000 | 0.0031 |
| PID_CASPASE_PATHWAY | 51 | -0.6497 | -1.8920 | 0.0021 | 0.0043 |
| PID_BARD1_PATHWAY | 29 | -0.7855 | -1.8819 | 0.0000 | 0.0048 |
| PID_ARF6_DOWNSTREAM_PATHWAY | 15 | -0.7668 | -1.8813 | 0.0000 | 0.0045 |
| PID_ATR_PATHWAY | 39 | -0.7667 | -1.8755 | 0.0020 | 0.0050 |
| PID_P53_REGULATION_PATHWAY | 59 | -0.6677 | -1.8680 | 0.0000 | 0.0053 |
| PID_NCADHERIN_PATHWAY | 33 | -0.6920 | -1.8504 | 0.0021 | 0.0072 |
| PID_PDGFRB_PATHWAY | 129 | -0.6225 | -1.8435 | 0.0022 | 0.0078 |
| PID_ERBB1_DOWNSTREAM_PATHWAY | 105 | -0.6186 | -1.8410 | 0.0000 | 0.0076 |
| PID_ERB_GENOMIC_PATHWAY | 15 | -0.6799 | -1.8332 | 0.0021 | 0.0078 |
| PID_BETA_CATENIN_NUC_PATHWAY | 78 | -0.5555 | -1.8317 | 0.0000 | 0.0080 |
| PID_PS1_PATHWAY | 45 | -0.6055 | -1.8170 | 0.0020 | 0.0094 |
| PID_P38_ALPHA_BETA_DOWNSTREAM_PATHWAY | 38 | -0.6070 | -1.8110 | 0.0041 | 0.0100 |
| PID_AJDISS_2PATHWAY | 48 | -0.6145 | -1.8073 | 0.0000 | 0.0101 |
| PID_NOTCH_PATHWAY | 59 | -0.6152 | -1.8068 | 0.0000 | 0.0099 |
| PID_AURORA_A_PATHWAY | 31 | -0.6714 | -1.7983 | 0.0085 | 0.0109 |
| PID_ECADHERIN_NASCENT_AJ_PATHWAY | 39 | -0.6497 | -1.7960 | 0.0021 | 0.0108 |
| PID_HIF1A_PATHWAY | 19 | -0.6778 | -1.7898 | 0.0064 | 0.0116 |
| PID_P73PATHWAY | 79 | -0.5544 | -1.7851 | 0.0021 | 0.0122 |
| PID_WNT_CANONICAL_PATHWAY | 20 | -0.6790 | -1.7793 | 0.0042 | 0.0128 |
| PID_FANCONI_PATHWAY | 47 | -0.7254 | -1.7745 | 0.0041 | 0.0132 |
| PID_P38_MK2_PATHWAY | 21 | -0.7070 | -1.7643 | 0.0000 | 0.0146 |
| PID_CERAMIDE_PATHWAY | 44 | -0.5890 | -1.7581 | 0.0086 | 0.0155 |
| PID_CIRCADIAN_PATHWAY | 16 | -0.7024 | -1.7568 | 0.0081 | 0.0154 |
| PID_ATM_PATHWAY | 34 | -0.7157 | -1.7564 | 0.0000 | 0.0150 |
| PID_AR_NONGENOMIC_PATHWAY | 31 | -0.6090 | -1.7538 | 0.0065 | 0.0150 |
| PID_HDAC_CLASSI_PATHWAY | 66 | -0.5821 | -1.7499 | 0.0021 | 0.0155 |
| PID_RHOA_PATHWAY | 44 | -0.6147 | -1.7497 | 0.0021 | 0.0152 |
| PID_TELOMERASE_PATHWAY | 67 | -0.6055 | -1.7460 | 0.0060 | 0.0154 |
| PID_ALPHA_SYNUCLEIN_PATHWAY | 32 | -0.6027 | -1.7325 | 0.0041 | 0.0179 |
| PID_ERBB2_ERBB3_PATHWAY | 44 | -0.5902 | -1.7257 | 0.0042 | 0.0190 |
| PID_PI3KCI_PATHWAY | 48 | -0.6288 | -1.7255 | 0.0124 | 0.0186 |
| PID_P53_DOWNSTREAM_PATHWAY | 137 | -0.5122 | -1.7252 | 0.0063 | 0.0182 |
| PID_BETA_CATENIN_DEG_PATHWAY | 18 | -0.6642 | -1.7160 | 0.0106 | 0.0198 |
| PID_CXCR3_PATHWAY | 43 | -0.5697 | -1.7142 | 0.0124 | 0.0196 |
| PID_MET_PATHWAY | 79 | -0.5860 | -1.7127 | 0.0167 | 0.0195 |
| PID_HDAC_CLASSII_PATHWAY | 34 | -0.6058 | -1.7095 | 0.0021 | 0.0196 |
| PID_REG_GR_PATHWAY | 82 | -0.5048 | -1.7051 | 0.0041 | 0.0213 |
| PID_VEGFR1_2_PATHWAY | 69 | -0.5796 | -1.6978 | 0.0106 | 0.0225 |
| PID_AURORA_B_PATHWAY | 39 | -0.6730 | -1.6978 | 0.0144 | 0.0221 |
| PID_REELIN_PATHWAY | 28 | -0.6159 | -1.6943 | 0.0063 | 0.0225 |
| PID_CXCR4_PATHWAY | 101 | -0.5780 | -1.6867 | 0.0229 | 0.0238 |
| PID_FAK_PATHWAY | 59 | -0.6211 | -1.6846 | 0.0106 | 0.0240 |
| PID_ALK1_PATHWAY | 26 | -0.6192 | -1.6837 | 0.0141 | 0.0237 |
| PID_S1P_S1P1_PATHWAY | 21 | -0.6390 | -1.6823 | 0.0205 | 0.0237 |
| PID_ANTHRAX_PATHWAY | 18 | -0.6426 | -1.6801 | 0.0126 | 0.0239 |
| PID_RHOA_REG_PATHWAY | 46 | -0.6124 | -1.6767 | 0.0042 | 0.0243 |
| PID_MAPK_TRK_PATHWAY | 34 | -0.6024 | -1.6747 | 0.0146 | 0.0243 |
| PID_EPHB_FWD_PATHWAY | 40 | -0.5832 | -1.6741 | 0.0227 | 0.0241 |
| PID_NFAT_3PATHWAY | 53 | -0.5860 | -1.6740 | 0.0063 | 0.0237 |
| PID_INSULIN_PATHWAY | 44 | -0.5902 | -1.6732 | 0.0166 | 0.0236 |
| PID_PI3KCI_AKT_PATHWAY | 35 | -0.5742 | -1.6637 | 0.0186 | 0.0253 |
| PID_SYNDECAN_2_PATHWAY | 33 | -0.5627 | -1.6634 | 0.0170 | 0.0251 |
| PID_ECADHERIN_STABILIZATION_PATHWAY | 41 | -0.5955 | -1.6598 | 0.0226 | 0.0256 |
| PID_P75_NTR_PATHWAY | 68 | -0.5183 | -1.6567 | 0.0143 | 0.0259 |
| PID_AR_PATHWAY | 61 | -0.5669 | -1.6540 | 0.0186 | 0.0263 |
| PID_A6B1_A6B4_INTEGRIN_PATHWAY | 46 | -0.5492 | -1.6516 | 0.0258 | 0.0265 |
| PID_CMYB_PATHWAY | 84 | -0.5115 | -1.6476 | 0.0105 | 0.0270 |
| PID_MYC_REPRESS_PATHWAY | 63 | -0.5020 | -1.6474 | 0.0124 | 0.0266 |
| PID_TGFBR_PATHWAY | 53 | -0.5840 | -1.6463 | 0.0148 | 0.0267 |
| PID_TRKR_PATHWAY | 61 | -0.5448 | -1.6411 | 0.0166 | 0.0274 |
| PID_E2F_PATHWAY | 73 | -0.5814 | -1.6396 | 0.0202 | 0.0274 |
| PID_IL8_CXCR2_PATHWAY | 34 | -0.6305 | -1.6377 | 0.0227 | 0.0275 |
| PID_DELTA_NP63_PATHWAY | 47 | -0.5261 | -1.6343 | 0.0021 | 0.0283 |
| PID_ERBB1_INTERNALIZATION_PATHWAY | 41 | -0.5859 | -1.6312 | 0.0187 | 0.0288 |
| PID_TNF_PATHWAY | 46 | -0.5786 | -1.6309 | 0.0255 | 0.0285 |
| PID_NECTIN_PATHWAY | 30 | -0.6291 | -1.6295 | 0.0310 | 0.0284 |
| PID_WNT_SIGNALING_PATHWAY | 28 | -0.5547 | -1.6272 | 0.0083 | 0.0288 |
| PID_FCER1_PATHWAY | 60 | -0.5681 | -1.6258 | 0.0299 | 0.0288 |
| PID_SYNDECAN_4_PATHWAY | 32 | -0.5225 | -1.6258 | 0.0265 | 0.0285 |
| PID_ERBB1_RECEPTOR_PROXIMAL_PATHWAY | 35 | -0.6076 | -1.6237 | 0.0214 | 0.0287 |
| PID_FOXO_PATHWAY | 49 | -0.5520 | -1.6236 | 0.0122 | 0.0284 |
| PID_TAP63_PATHWAY | 54 | -0.5101 | -1.6225 | 0.0228 | 0.0283 |
| PID_EPHA2_FWD_PATHWAY | 19 | -0.6227 | -1.6218 | 0.0201 | 0.0281 |
| PID_KIT_PATHWAY | 52 | -0.5718 | -1.6169 | 0.0193 | 0.0292 |
| PID_RAS_PATHWAY | 30 | -0.5692 | -1.6073 | 0.0151 | 0.0315 |
| PID_TCPTP_PATHWAY | 42 | -0.5616 | -1.5962 | 0.0354 | 0.0342 |
| PID_ENDOTHELIN_PATHWAY | 63 | -0.5343 | -1.5881 | 0.0289 | 0.0364 |
| PID_RAC1_REG_PATHWAY | 38 | -0.5746 | -1.5838 | 0.0211 | 0.0375 |
| PID_HIV_NEF_PATHWAY | 35 | -0.5684 | -1.5837 | 0.0357 | 0.0372 |
| PID_ERA_GENOMIC_PATHWAY | 64 | -0.4600 | -1.5828 | 0.0198 | 0.0371 |
| PID_HEDGEHOG_GLI_PATHWAY | 48 | -0.5591 | -1.5750 | 0.0323 | 0.0391 |
| PID_PI3K_PLC_TRK_PATHWAY | 36 | -0.5579 | -1.5748 | 0.0292 | 0.0388 |
| PID_EPHA_FWDPATHWAY | 34 | -0.5474 | -1.5653 | 0.0411 | 0.0422 |
| PID_HIF2PATHWAY | 34 | -0.5146 | -1.5603 | 0.0342 | 0.0443 |
| PID_SMAD2_3NUCLEAR_PATHWAY | 82 | -0.5191 | -1.5597 | 0.0410 | 0.0441 |
| PID_SMAD2_3PATHWAY | 16 | -0.6749 | -1.5522 | 0.0441 | 0.0463 |
| PID_TXA2PATHWAY | 56 | -0.5502 | -1.5521 | 0.0607 | 0.0459 |
| PID_AR_TF_PATHWAY | 53 | -0.5152 | -1.5512 | 0.0420 | 0.0458 |
| PID_S1P_S1P3_PATHWAY | 29 | -0.5912 | -1.5482 | 0.0593 | 0.0465 |
| PID_P38_ALPHA_BETA_PATHWAY | 30 | -0.5392 | -1.5473 | 0.0585 | 0.0464 |
| PID_NFAT_TFPATHWAY | 45 | -0.5371 | -1.5471 | 0.0696 | 0.0460 |
| PID_THROMBIN_PAR1_PATHWAY | 43 | -0.5477 | -1.5457 | 0.0346 | 0.0459 |
| PID_IFNG_PATHWAY | 40 | -0.5540 | -1.5314 | 0.0511 | 0.0507 |
| PID_IL2_PI3K_PATHWAY | 34 | -0.5471 | -1.5290 | 0.0455 | 0.0513 |
| PID_ARF6_TRAFFICKING_PATHWAY | 49 | -0.5442 | -1.5276 | 0.0538 | 0.0513 |
| PID_FAS_PATHWAY | 38 | -0.5473 | -1.5257 | 0.0517 | 0.0517 |
| PID_LYSOPHOSPHOLIPID_PATHWAY | 65 | -0.5081 | -1.5256 | 0.0679 | 0.0512 |
| PID_IGF1_PATHWAY | 29 | -0.5576 | -1.5254 | 0.0456 | 0.0508 |
| PID_CD8_TCR_DOWNSTREAM_PATHWAY | 65 | -0.5215 | -1.5226 | 0.0657 | 0.0514 |
| PID_NEPHRIN_NEPH1_PATHWAY | 31 | -0.5455 | -1.5170 | 0.0597 | 0.0534 |
| PID_S1P_S1P2_PATHWAY | 24 | -0.5720 | -1.5152 | 0.0701 | 0.0538 |
| PID_FGF_PATHWAY | 55 | -0.4801 | -1.5134 | 0.0494 | 0.0538 |
| PID_IL8_CXCR1_PATHWAY | 28 | -0.5899 | -1.5070 | 0.0538 | 0.0555 |
| PID_ECADHERIN_KERATINOCYTE_PATHWAY | 21 | -0.5814 | -1.5065 | 0.0643 | 0.0554 |
| PID_PTP1B_PATHWAY | 52 | -0.5061 | -1.4988 | 0.0769 | 0.0581 |
| PID_CDC42_REG_PATHWAY | 30 | -0.5988 | -1.4924 | 0.0649 | 0.0604 |
| PID_ER_NONGENOMIC_PATHWAY | 40 | -0.5346 | -1.4898 | 0.0819 | 0.0609 |
| PID_NFKAPPAB_CANONICAL_PATHWAY | 23 | -0.5713 | -1.4800 | 0.0934 | 0.0649 |
| PID_TCR_PATHWAY | 65 | -0.5688 | -1.4633 | 0.1125 | 0.0718 |
| PID_BCR_5PATHWAY | 63 | -0.5182 | -1.4597 | 0.0974 | 0.0730 |
| PID_AP1_PATHWAY | 69 | -0.4551 | -1.4504 | 0.0701 | 0.0764 |
| PID_AVB3_OPN_PATHWAY | 31 | -0.5391 | -1.4497 | 0.0965 | 0.0761 |
| PID_RXR_VDR_PATHWAY | 26 | -0.4980 | -1.4434 | 0.0820 | 0.0785 |
| PID_CD8_TCR_PATHWAY | 53 | -0.5662 | -1.4428 | 0.1186 | 0.0782 |
| PID_NFKAPPAB_ATYPICAL_PATHWAY | 17 | -0.5494 | -1.4417 | 0.0896 | 0.0779 |
| PID_NETRIN_PATHWAY | 32 | -0.5369 | -1.4393 | 0.1002 | 0.0784 |
| PID_INTEGRIN_A4B1_PATHWAY | 33 | -0.5002 | -1.4382 | 0.0941 | 0.0782 |
| PID_VEGFR1_PATHWAY | 26 | -0.5448 | -1.4379 | 0.1086 | 0.0778 |
| PID_BMP_PATHWAY | 42 | -0.4828 | -1.4277 | 0.0747 | 0.0818 |
| PID_RETINOIC_ACID_PATHWAY | 30 | -0.5011 | -1.4275 | 0.0978 | 0.0813 |
| PID_SYNDECAN_3_PATHWAY | 17 | -0.5599 | -1.4222 | 0.0690 | 0.0841 |
| PID_GMCSF_PATHWAY | 36 | -0.5143 | -1.4186 | 0.1048 | 0.0848 |
| PID_GLYPICAN_1PATHWAY | 27 | -0.5240 | -1.4110 | 0.1025 | 0.0884 |
| PID_HES_HEY_PATHWAY | 48 | -0.4855 | -1.4105 | 0.1025 | 0.0880 |
| PID_TRAIL_PATHWAY | 28 | -0.5134 | -1.4092 | 0.1004 | 0.0879 |
| PID_EPHRINB_REV_PATHWAY | 30 | -0.5049 | -1.4052 | 0.1043 | 0.0891 |
| PID_S1P_META_PATHWAY | 21 | -0.5251 | -1.4005 | 0.1327 | 0.0908 |
| PID_IL2_STAT5_PATHWAY | 30 | -0.5340 | -1.3956 | 0.1341 | 0.0923 |
| PID_IL1_PATHWAY | 34 | -0.5198 | -1.3939 | 0.1072 | 0.0925 |
| PID_EPO_PATHWAY | 33 | -0.5046 | -1.3931 | 0.1378 | 0.0923 |
| PID_RET_PATHWAY | 39 | -0.4833 | -1.3798 | 0.1245 | 0.0987 |
| PID_FRA_PATHWAY | 37 | -0.5020 | -1.3794 | 0.1495 | 0.0981 |
| PID_HIF1_TFPATHWAY | 66 | -0.4293 | -1.3680 | 0.1149 | 0.1036 |
| PID_PDGFRA_PATHWAY | 22 | -0.5063 | -1.3526 | 0.1626 | 0.1123 |
| PID_ARF6_PATHWAY | 35 | -0.4588 | -1.3471 | 0.1245 | 0.1147 |
| PID_TCR_CALCIUM_PATHWAY | 28 | -0.4832 | -1.3383 | 0.1721 | 0.1195 |
| PID_ANGIOPOIETIN_RECEPTOR_PATHWAY | 49 | -0.4390 | -1.3261 | 0.1879 | 0.1257 |
| PID_IL2_1PATHWAY | 55 | -0.4613 | -1.3233 | 0.1745 | 0.1263 |
| PID_IL12_STAT4_PATHWAY | 33 | -0.5221 | -1.3066 | 0.2063 | 0.1346 |
| PID_HDAC_CLASSIII_PATHWAY | 26 | -0.4354 | -1.2981 | 0.1736 | 0.1386 |
| PID_AVB3_INTEGRIN_PATHWAY | 74 | -0.4396 | -1.2950 | 0.2238 | 0.1395 |
| PID_INTEGRIN_CS_PATHWAY | 26 | -0.4902 | -1.2869 | 0.2135 | 0.1435 |
| PID_P38_MKK3_6PATHWAY | 26 | -0.4412 | -1.2845 | 0.1879 | 0.1443 |
| PID_CD40_PATHWAY | 31 | -0.4566 | -1.2751 | 0.2206 | 0.1487 |
| PID_IL12_2PATHWAY | 63 | -0.4600 | -1.2556 | 0.2641 | 0.1603 |
| PID_THROMBIN_PAR4_PATHWAY | 15 | -0.5053 | -1.2531 | 0.2045 | 0.1610 |
| PID_IL4_2PATHWAY | 64 | -0.3926 | -1.2361 | 0.2056 | 0.1709 |
| PID_SYNDECAN_1_PATHWAY | 46 | -0.4449 | -1.2358 | 0.2211 | 0.1703 |
| PID_ATF2_PATHWAY | 59 | -0.3794 | -1.2265 | 0.2046 | 0.1757 |
| PID_SHP2_PATHWAY | 57 | -0.4055 | -1.2239 | 0.2590 | 0.1767 |
| PID_LYMPH_ANGIOGENESIS_PATHWAY | 25 | -0.4537 | -1.2148 | 0.2649 | 0.1818 |
| PID_IL3_PATHWAY | 26 | -0.4176 | -1.2041 | 0.2524 | 0.1876 |
| PID_INTEGRIN_A9B1_PATHWAY | 25 | -0.4220 | -1.1935 | 0.2777 | 0.1938 |
| PID_IL23_PATHWAY | 37 | -0.4390 | -1.1895 | 0.2851 | 0.1951 |
| PID_IL27_PATHWAY | 26 | -0.4601 | -1.1767 | 0.3027 | 0.2031 |
| PID_IL6_7_PATHWAY | 47 | -0.3857 | -1.1532 | 0.2866 | 0.2201 |
| PID_ERBB4_PATHWAY | 38 | -0.3778 | -1.1333 | 0.3259 | 0.2330 |
| PID_AMB2_NEUTROPHILS_PATHWAY | 40 | -0.4047 | -1.1287 | 0.3354 | 0.2361 |
| PID_TOLL_ENDOGENOUS_PATHWAY | 24 | -0.4007 | -1.1260 | 0.3051 | 0.2367 |
| PID_ERBB_NETWORK_PATHWAY | 15 | -0.4568 | -1.1260 | 0.3354 | 0.2354 |
| PID_UPA_UPAR_PATHWAY | 42 | -0.3803 | -1.1146 | 0.3222 | 0.2430 |
| PID_LPA4_PATHWAY | 15 | -0.4127 | -1.0384 | 0.4088 | 0.3048 |
| PID_HEDGEHOG_2PATHWAY | 22 | -0.3713 | -1.0166 | 0.4255 | 0.3238 |
| PID_RHODOPSIN_PATHWAY | 23 | -0.3132 | -0.9681 | 0.5010 | 0.3725 |
| PID_INTEGRIN5_PATHWAY | 16 | -0.3693 | -0.9420 | 0.5080 | 0.3964 |
| PID_CONE_PATHWAY | 22 | -0.3103 | -0.9081 | 0.5948 | 0.4329 |
| PID_INTEGRIN3_PATHWAY | 42 | -0.3168 | -0.8802 | 0.6037 | 0.4622 |
| PID_INTEGRIN1_PATHWAY | 66 | -0.2961 | -0.8596 | 0.5918 | 0.4826 |

| **Signaling pathways enriched in HCC samples corresponding ADH4 expression by GSEA based on PID** | | | | | |
| --- | --- | --- | --- | --- | --- |
| **PID Name** | **Size** | **ES** | **NES** | **NOM p-value** | **FDR q-value** |
| PID_HNF3B_PATHWAY | 45 | 0.4575 | 1.5074 | 0.0513 | 0.0822 |
| PID_HNF3A_PATHWAY | 44 | 0.3592 | 1.1868 | 0.2302 | 0.1872 |
| PID_RAC1_PATHWAY | 54 | -0.7584 | -2.1682 | 0.0000 | 0.0020 |
| PID_LKB1_PATHWAY | 47 | -0.6814 | -2.0332 | 0.0000 | 0.0075 |
| PID_PRL_SIGNALING_EVENTS_PATHWAY | 23 | -0.7168 | -2.0260 | 0.0000 | 0.0050 |
| PID_P38_MK2_PATHWAY | 21 | -0.7973 | -1.9848 | 0.0000 | 0.0094 |
| PID_DELTA_NP63_PATHWAY | 47 | -0.6259 | -1.9812 | 0.0000 | 0.0077 |
| PID_PLK1_PATHWAY | 46 | -0.7698 | -1.9805 | 0.0000 | 0.0066 |
| PID_ATR_PATHWAY | 39 | -0.7929 | -1.9745 | 0.0000 | 0.0060 |
| PID_HIF1A_PATHWAY | 19 | -0.7279 | -1.9731 | 0.0000 | 0.0052 |
| PID_RB_1PATHWAY | 65 | -0.6500 | -1.9631 | 0.0000 | 0.0055 |
| PID_PDGFRB_PATHWAY | 129 | -0.6492 | -1.9593 | 0.0019 | 0.0052 |
| PID_P73PATHWAY | 79 | -0.5961 | -1.9579 | 0.0000 | 0.0048 |
| PID_ERBB1_DOWNSTREAM_PATHWAY | 105 | -0.6393 | -1.9511 | 0.0020 | 0.0047 |
| PID_FOXM1_PATHWAY | 40 | -0.7177 | -1.9469 | 0.0000 | 0.0045 |
| PID_ILK_PATHWAY | 45 | -0.7069 | -1.9464 | 0.0000 | 0.0043 |
| PID_ARF_3PATHWAY | 19 | -0.8029 | -1.9441 | 0.0000 | 0.0041 |
| PID_RHOA_PATHWAY | 44 | -0.6697 | -1.9413 | 0.0000 | 0.0040 |
| PID_ARF6_DOWNSTREAM_PATHWAY | 15 | -0.7857 | -1.9314 | 0.0000 | 0.0047 |
| PID_CDC42_PATHWAY | 70 | -0.6442 | -1.9102 | 0.0020 | 0.0059 |
| PID_INSULIN_GLUCOSE_PATHWAY | 26 | -0.6870 | -1.9075 | 0.0000 | 0.0056 |
| PID_BETA_CATENIN_NUC_PATHWAY | 78 | -0.5856 | -1.9015 | 0.0000 | 0.0058 |
| PID_MTOR_4PATHWAY | 69 | -0.6293 | -1.8927 | 0.0019 | 0.0062 |
| PID_TAP63_PATHWAY | 54 | -0.5912 | -1.8706 | 0.0019 | 0.0083 |
| PID_ECADHERIN_STABILIZATION_PATHWAY | 41 | -0.6716 | -1.8686 | 0.0038 | 0.0082 |
| PID_NOTCH_PATHWAY | 59 | -0.6115 | -1.8553 | 0.0000 | 0.0093 |
| PID_P53_DOWNSTREAM_PATHWAY | 137 | -0.5341 | -1.8519 | 0.0000 | 0.0100 |
| PID_CASPASE_PATHWAY | 51 | -0.6272 | -1.8490 | 0.0019 | 0.0098 |
| PID_MYC_REPRESS_PATHWAY | 63 | -0.5642 | -1.8473 | 0.0000 | 0.0096 |
| PID_TNF_PATHWAY | 46 | -0.6465 | -1.8472 | 0.0059 | 0.0093 |
| PID_TELOMERASE_PATHWAY | 67 | -0.6431 | -1.8452 | 0.0040 | 0.0091 |
| PID_ERBB2_ERBB3_PATHWAY | 44 | -0.6190 | -1.8371 | 0.0020 | 0.0094 |
| PID_ATM_PATHWAY | 34 | -0.7279 | -1.8354 | 0.0019 | 0.0092 |
| PID_MYC_ACTIV_PATHWAY | 79 | -0.6227 | -1.8345 | 0.0019 | 0.0090 |
| PID_CERAMIDE_PATHWAY | 44 | -0.6118 | -1.8334 | 0.0039 | 0.0090 |
| PID_P38_ALPHA_BETA_DOWNSTREAM_PATHWAY | 38 | -0.6061 | -1.8232 | 0.0058 | 0.0099 |
| PID_BARD1_PATHWAY | 29 | -0.7491 | -1.8214 | 0.0019 | 0.0099 |
| PID_WNT_NONCANONICAL_PATHWAY | 32 | -0.6648 | -1.8167 | 0.0059 | 0.0102 |
| PID_MAPK_TRK_PATHWAY | 34 | -0.6465 | -1.8121 | 0.0020 | 0.0107 |
| PID_LIS1_PATHWAY | 28 | -0.6666 | -1.8081 | 0.0000 | 0.0111 |
| PID_A6B1_A6B4_INTEGRIN_PATHWAY | 46 | -0.6037 | -1.8015 | 0.0038 | 0.0117 |
| PID_P53_REGULATION_PATHWAY | 59 | -0.6464 | -1.7997 | 0.0039 | 0.0115 |
| PID_CXCR3_PATHWAY | 43 | -0.6010 | -1.7983 | 0.0080 | 0.0114 |
| PID_WNT_SIGNALING_PATHWAY | 28 | -0.6099 | -1.7965 | 0.0019 | 0.0114 |
| PID_CXCR4_PATHWAY | 101 | -0.6224 | -1.7945 | 0.0101 | 0.0113 |
| PID_PS1_PATHWAY | 45 | -0.6018 | -1.7920 | 0.0097 | 0.0112 |
| PID_AURORA_A_PATHWAY | 31 | -0.6515 | -1.7861 | 0.0116 | 0.0116 |
| PID_HDAC_CLASSI_PATHWAY | 66 | -0.5830 | -1.7758 | 0.0038 | 0.0126 |
| PID_MYC_PATHWAY | 25 | -0.6878 | -1.7733 | 0.0040 | 0.0127 |
| PID_IL8_CXCR2_PATHWAY | 34 | -0.6818 | -1.7715 | 0.0039 | 0.0128 |
| PID_SYNDECAN_2_PATHWAY | 33 | -0.6018 | -1.7693 | 0.0098 | 0.0127 |
| PID_RAS_PATHWAY | 30 | -0.6153 | -1.7611 | 0.0040 | 0.0137 |
| PID_ERBB1_INTERNALIZATION_PATHWAY | 41 | -0.6213 | -1.7607 | 0.0078 | 0.0135 |
| PID_RHOA_REG_PATHWAY | 46 | -0.6342 | -1.7585 | 0.0039 | 0.0134 |
| PID_GMCSF_PATHWAY | 36 | -0.6240 | -1.7502 | 0.0121 | 0.0145 |
| PID_FANCONI_PATHWAY | 47 | -0.7057 | -1.7466 | 0.0019 | 0.0148 |
| PID_DNA_PK_PATHWAY | 16 | -0.7020 | -1.7437 | 0.0059 | 0.0150 |
| PID_EPHA2_FWD_PATHWAY | 19 | -0.6652 | -1.7431 | 0.0097 | 0.0148 |
| PID_PI3K_PLC_TRK_PATHWAY | 36 | -0.6037 | -1.7396 | 0.0095 | 0.0153 |
| PID_PI3KCI_PATHWAY | 48 | -0.6440 | -1.7393 | 0.0122 | 0.0150 |
| PID_FGF_PATHWAY | 55 | -0.5624 | -1.7332 | 0.0020 | 0.0165 |
| PID_ECADHERIN_NASCENT_AJ_PATHWAY | 39 | -0.6408 | -1.7312 | 0.0121 | 0.0164 |
| PID_TRKR_PATHWAY | 61 | -0.5798 | -1.7298 | 0.0062 | 0.0163 |
| PID_WNT_CANONICAL_PATHWAY | 20 | -0.6649 | -1.7282 | 0.0056 | 0.0162 |
| PID_ARF6_TRAFFICKING_PATHWAY | 49 | -0.6074 | -1.7281 | 0.0121 | 0.0160 |
| PID_AURORA_B_PATHWAY | 39 | -0.6736 | -1.7275 | 0.0214 | 0.0158 |
| PID_MET_PATHWAY | 79 | -0.5868 | -1.7224 | 0.0175 | 0.0164 |
| PID_P75_NTR_PATHWAY | 68 | -0.5362 | -1.7177 | 0.0019 | 0.0170 |
| PID_CMYB_PATHWAY | 84 | -0.5158 | -1.7173 | 0.0039 | 0.0167 |
| PID_VEGFR1_2_PATHWAY | 69 | -0.5908 | -1.7130 | 0.0140 | 0.0171 |
| PID_TGFBR_PATHWAY | 53 | -0.6032 | -1.7105 | 0.0229 | 0.0172 |
| PID_TRAIL_PATHWAY | 28 | -0.6086 | -1.7091 | 0.0190 | 0.0172 |
| PID_EPHB_FWD_PATHWAY | 40 | -0.6238 | -1.7087 | 0.0103 | 0.0170 |
| PID_NFAT_3PATHWAY | 53 | -0.5975 | -1.7067 | 0.0136 | 0.0170 |
| PID_PI3KCI_AKT_PATHWAY | 35 | -0.5934 | -1.7022 | 0.0097 | 0.0176 |
| PID_ERB_GENOMIC_PATHWAY | 15 | -0.6449 | -1.6914 | 0.0101 | 0.0192 |
| PID_HDAC_CLASSII_PATHWAY | 34 | -0.6020 | -1.6879 | 0.0116 | 0.0196 |
| PID_ALK1_PATHWAY | 26 | -0.6347 | -1.6844 | 0.0100 | 0.0199 |
| PID_CIRCADIAN_PATHWAY | 16 | -0.6706 | -1.6820 | 0.0096 | 0.0201 |
| PID_ERBB1_RECEPTOR_PROXIMAL_PATHWAY | 35 | -0.6235 | -1.6796 | 0.0276 | 0.0204 |
| PID_E2F_PATHWAY | 73 | -0.5851 | -1.6788 | 0.0273 | 0.0203 |
| PID_REG_GR_PATHWAY | 82 | -0.4956 | -1.6741 | 0.0079 | 0.0211 |
| PID_ALPHA_SYNUCLEIN_PATHWAY | 32 | -0.5909 | -1.6728 | 0.0136 | 0.0209 |
| PID_AR_PATHWAY | 61 | -0.5685 | -1.6664 | 0.0292 | 0.0223 |
| PID_P38_ALPHA_BETA_PATHWAY | 30 | -0.5890 | -1.6655 | 0.0116 | 0.0223 |
| PID_INTEGRIN_CS_PATHWAY | 26 | -0.6508 | -1.6652 | 0.0103 | 0.0220 |
| PID_HES_HEY_PATHWAY | 48 | -0.5662 | -1.6643 | 0.0099 | 0.0220 |
| PID_INTEGRIN_A4B1_PATHWAY | 33 | -0.5804 | -1.6572 | 0.0216 | 0.0232 |
| PID_IL8_CXCR1_PATHWAY | 28 | -0.6381 | -1.6566 | 0.0236 | 0.0230 |
| PID_S1P_S1P3_PATHWAY | 29 | -0.6378 | -1.6444 | 0.0105 | 0.0256 |
| PID_LYSOPHOSPHOLIPID_PATHWAY | 65 | -0.5350 | -1.6416 | 0.0317 | 0.0260 |
| PID_HIV_NEF_PATHWAY | 35 | -0.5880 | -1.6415 | 0.0239 | 0.0258 |
| PID_SMAD2_3PATHWAY | 16 | -0.7238 | -1.6409 | 0.0173 | 0.0257 |
| PID_KIT_PATHWAY | 52 | -0.5772 | -1.6393 | 0.0261 | 0.0258 |
| PID_ARF6_PATHWAY | 35 | -0.5465 | -1.6369 | 0.0174 | 0.0261 |
| PID_HIF2PATHWAY | 34 | -0.5354 | -1.6326 | 0.0344 | 0.0270 |
| PID_EPHA_FWDPATHWAY | 34 | -0.5613 | -1.6322 | 0.0273 | 0.0268 |
| PID_PTP1B_PATHWAY | 52 | -0.5487 | -1.6287 | 0.0198 | 0.0272 |
| PID_AR_NONGENOMIC_PATHWAY | 31 | -0.5703 | -1.6245 | 0.0148 | 0.0279 |
| PID_TCPTP_PATHWAY | 42 | -0.5704 | -1.6238 | 0.0297 | 0.0277 |
| PID_NECTIN_PATHWAY | 30 | -0.6329 | -1.6179 | 0.0262 | 0.0290 |
| PID_FAS_PATHWAY | 38 | -0.5700 | -1.6122 | 0.0307 | 0.0300 |
| PID_SMAD2_3NUCLEAR_PATHWAY | 82 | -0.5341 | -1.6072 | 0.0317 | 0.0310 |
| PID_FAK_PATHWAY | 59 | -0.5939 | -1.6054 | 0.0373 | 0.0312 |
| PID_INSULIN_PATHWAY | 44 | -0.5683 | -1.6029 | 0.0394 | 0.0317 |
| PID_ERA_GENOMIC_PATHWAY | 64 | -0.4560 | -1.6016 | 0.0133 | 0.0317 |
| PID_RAC1_REG_PATHWAY | 38 | -0.5733 | -1.5971 | 0.0215 | 0.0325 |
| PID_S1P_S1P1_PATHWAY | 21 | -0.6147 | -1.5925 | 0.0399 | 0.0335 |
| PID_REELIN_PATHWAY | 28 | -0.5891 | -1.5919 | 0.0233 | 0.0332 |
| PID_INTEGRIN_A9B1_PATHWAY | 25 | -0.5662 | -1.5883 | 0.0316 | 0.0338 |
| PID_ER_NONGENOMIC_PATHWAY | 40 | -0.5678 | -1.5858 | 0.0353 | 0.0341 |
| PID_SYNDECAN_3_PATHWAY | 17 | -0.6177 | -1.5854 | 0.0172 | 0.0338 |
| PID_AJDISS_2PATHWAY | 48 | -0.5331 | -1.5818 | 0.0235 | 0.0347 |
| PID_FRA_PATHWAY | 37 | -0.5834 | -1.5817 | 0.0222 | 0.0344 |
| PID_ANTHRAX_PATHWAY | 18 | -0.6167 | -1.5798 | 0.0420 | 0.0347 |
| PID_IL2_PI3K_PATHWAY | 34 | -0.5580 | -1.5738 | 0.0514 | 0.0365 |
| PID_P38_MKK3_6PATHWAY | 26 | -0.5278 | -1.5691 | 0.0373 | 0.0381 |
| PID_TXA2PATHWAY | 56 | -0.5534 | -1.5623 | 0.0595 | 0.0399 |
| PID_BCR_5PATHWAY | 63 | -0.5741 | -1.5572 | 0.0438 | 0.0414 |
| PID_HEDGEHOG_GLI_PATHWAY | 48 | -0.5529 | -1.5550 | 0.0499 | 0.0417 |
| PID_ECADHERIN_KERATINOCYTE_PATHWAY | 21 | -0.6013 | -1.5544 | 0.0289 | 0.0415 |
| PID_NCADHERIN_PATHWAY | 33 | -0.5890 | -1.5527 | 0.0510 | 0.0418 |
| PID_THROMBIN_PAR1_PATHWAY | 43 | -0.5381 | -1.5473 | 0.0550 | 0.0430 |
| PID_NEPHRIN_NEPH1_PATHWAY | 31 | -0.5481 | -1.5471 | 0.0474 | 0.0427 |
| PID_ENDOTHELIN_PATHWAY | 63 | -0.5148 | -1.5458 | 0.0604 | 0.0427 |
| PID_BETA_CATENIN_DEG_PATHWAY | 18 | -0.6105 | -1.5423 | 0.0475 | 0.0435 |
| PID_IGF1_PATHWAY | 29 | -0.5608 | -1.5391 | 0.0494 | 0.0441 |
| PID_FCER1_PATHWAY | 60 | -0.5416 | -1.5389 | 0.0559 | 0.0438 |
| PID_FOXO_PATHWAY | 49 | -0.5291 | -1.5367 | 0.0660 | 0.0440 |
| PID_SYNDECAN_4_PATHWAY | 32 | -0.5164 | -1.5365 | 0.0201 | 0.0437 |
| PID_CDC42_REG_PATHWAY | 30 | -0.5860 | -1.5304 | 0.0603 | 0.0454 |
| PID_RET_PATHWAY | 39 | -0.5384 | -1.5202 | 0.0535 | 0.0483 |
| PID_NFKAPPAB_ATYPICAL_PATHWAY | 17 | -0.5842 | -1.5133 | 0.0531 | 0.0501 |
| PID_CD8_TCR_PATHWAY | 53 | -0.5954 | -1.5004 | 0.1046 | 0.0549 |
| PID_AP1_PATHWAY | 69 | -0.4820 | -1.4994 | 0.0423 | 0.0549 |
| PID_HIF1_TFPATHWAY | 66 | -0.4678 | -1.4967 | 0.0502 | 0.0554 |
| PID_AVB3_INTEGRIN_PATHWAY | 74 | -0.5233 | -1.4957 | 0.0681 | 0.0554 |
| PID_S1P_S1P2_PATHWAY | 24 | -0.5609 | -1.4946 | 0.0791 | 0.0553 |
| PID_CD8_TCR_DOWNSTREAM_PATHWAY | 65 | -0.5299 | -1.4933 | 0.0913 | 0.0559 |
| PID_IFNG_PATHWAY | 40 | -0.5368 | -1.4875 | 0.0746 | 0.0577 |
| PID_AVB3_OPN_PATHWAY | 31 | -0.5588 | -1.4764 | 0.0741 | 0.0617 |
| PID_EPO_PATHWAY | 33 | -0.5377 | -1.4717 | 0.0810 | 0.0630 |
| PID_TCR_PATHWAY | 65 | -0.5826 | -1.4716 | 0.1057 | 0.0626 |
| PID_GLYPICAN_1PATHWAY | 27 | -0.5535 | -1.4662 | 0.0656 | 0.0647 |
| PID_RXR_VDR_PATHWAY | 26 | -0.4960 | -1.4574 | 0.0720 | 0.0678 |
| PID_SYNDECAN_1_PATHWAY | 46 | -0.5365 | -1.4560 | 0.1058 | 0.0679 |
| PID_VEGFR1_PATHWAY | 26 | -0.5585 | -1.4428 | 0.1155 | 0.0739 |
| PID_IL2_1PATHWAY | 55 | -0.4979 | -1.4422 | 0.1233 | 0.0736 |
| PID_LYMPH_ANGIOGENESIS_PATHWAY | 25 | -0.5673 | -1.4394 | 0.1031 | 0.0756 |
| PID_ERBB_NETWORK_PATHWAY | 15 | -0.5732 | -1.4366 | 0.1012 | 0.0768 |
| PID_NETRIN_PATHWAY | 32 | -0.5324 | -1.4296 | 0.1098 | 0.0795 |
| PID_THROMBIN_PAR4_PATHWAY | 15 | -0.5680 | -1.4190 | 0.1067 | 0.0837 |
| PID_IL12_STAT4_PATHWAY | 33 | -0.5716 | -1.4123 | 0.1481 | 0.0862 |
| PID_NFKAPPAB_CANONICAL_PATHWAY | 23 | -0.5639 | -1.4075 | 0.1373 | 0.0883 |
| PID_TOLL_ENDOGENOUS_PATHWAY | 24 | -0.4958 | -1.4068 | 0.0931 | 0.0880 |
| PID_INTEGRIN5_PATHWAY | 16 | -0.5587 | -1.3954 | 0.1142 | 0.0931 |
| PID_AR_TF_PATHWAY | 53 | -0.4644 | -1.3927 | 0.1319 | 0.0943 |
| PID_IL2_STAT5_PATHWAY | 30 | -0.5292 | -1.3713 | 0.1608 | 0.1036 |
| PID_IL3_PATHWAY | 26 | -0.4731 | -1.3656 | 0.1361 | 0.1057 |
| PID_CD40_PATHWAY | 31 | -0.4942 | -1.3632 | 0.1297 | 0.1060 |
| PID_BMP_PATHWAY | 42 | -0.4631 | -1.3632 | 0.1065 | 0.1054 |
| PID_PDGFRA_PATHWAY | 22 | -0.5163 | -1.3554 | 0.1344 | 0.1092 |
| PID_NFAT_TFPATHWAY | 45 | -0.4909 | -1.3482 | 0.1622 | 0.1131 |
| PID_IL23_PATHWAY | 37 | -0.4961 | -1.3457 | 0.1831 | 0.1138 |
| PID_IL4_2PATHWAY | 64 | -0.4292 | -1.3327 | 0.1667 | 0.1209 |
| PID_UPA_UPAR_PATHWAY | 42 | -0.4539 | -1.3308 | 0.1398 | 0.1213 |
| PID_IL6_7_PATHWAY | 47 | -0.4475 | -1.3271 | 0.1594 | 0.1230 |
| PID_EPHRINB_REV_PATHWAY | 30 | -0.4679 | -1.3189 | 0.1540 | 0.1266 |
| PID_ATF2_PATHWAY | 59 | -0.4144 | -1.3182 | 0.1594 | 0.1262 |
| PID_IL27_PATHWAY | 26 | -0.5120 | -1.3154 | 0.1913 | 0.1271 |
| PID_IL1_PATHWAY | 34 | -0.4757 | -1.2988 | 0.1859 | 0.1358 |
| PID_IL12_2PATHWAY | 63 | -0.4856 | -1.2915 | 0.2433 | 0.1391 |
| PID_ERBB4_PATHWAY | 38 | -0.4300 | -1.2801 | 0.1885 | 0.1452 |
| PID_S1P_META_PATHWAY | 21 | -0.4674 | -1.2570 | 0.2432 | 0.1586 |
| PID_HDAC_CLASSIII_PATHWAY | 26 | -0.4117 | -1.2359 | 0.2327 | 0.1714 |
| PID_AMB2_NEUTROPHILS_PATHWAY | 40 | -0.4523 | -1.2292 | 0.2510 | 0.1740 |
| PID_SHP2_PATHWAY | 57 | -0.4036 | -1.2074 | 0.2520 | 0.1877 |
| PID_CONE_PATHWAY | 22 | -0.4098 | -1.1955 | 0.2322 | 0.1954 |
| PID_HEDGEHOG_2PATHWAY | 22 | -0.4338 | -1.1913 | 0.2126 | 0.1970 |
| PID_ANGIOPOIETIN_RECEPTOR_PATHWAY | 49 | -0.4052 | -1.1857 | 0.2715 | 0.1994 |
| PID_RETINOIC_ACID_PATHWAY | 30 | -0.4281 | -1.1840 | 0.2963 | 0.1993 |
| PID_TCR_CALCIUM_PATHWAY | 28 | -0.4387 | -1.1622 | 0.3101 | 0.2126 |
| PID_INTEGRIN3_PATHWAY | 42 | -0.4114 | -1.1490 | 0.3214 | 0.2202 |
| PID_INTEGRIN1_PATHWAY | 66 | -0.4048 | -1.1418 | 0.3293 | 0.2245 |
| PID_RHODOPSIN_PATHWAY | 23 | -0.3701 | -1.1239 | 0.2927 | 0.2379 |
| PID_LPA4_PATHWAY | 15 | -0.4257 | -1.0617 | 0.3955 | 0.2889 |
| PID_INTEGRIN2_PATHWAY | 28 | -0.3432 | -0.9272 | 0.5380 | 0.4184 |

| **Signaling pathways enriched in HCC samples corresponding ADH5 expression by GSEA based on PID** | | | | | |
| --- | --- | --- | --- | --- | --- |
| **PID Name** | **Size** | **ES** | **NES** | **NOM p-value** | **FDR q-value** |
| PID_HNF3B_PATHWAY | 45 | 0.5710 | 1.8437 | 0.0020 | 0.0506 |
| PID_RETINOIC_ACID_PATHWAY | 30 | 0.5349 | 1.4622 | 0.0768 | 1.0000 |
| PID_HNF3A_PATHWAY | 44 | 0.4246 | 1.3949 | 0.0617 | 1.0000 |
| PID_AR_TF_PATHWAY | 53 | 0.4541 | 1.3357 | 0.1517 | 1.0000 |
| PID_ERBB4_PATHWAY | 38 | 0.4369 | 1.3184 | 0.1572 | 0.9339 |
| PID_FOXO_PATHWAY | 49 | 0.4366 | 1.2333 | 0.2541 | 1.0000 |
| PID_BMP_PATHWAY | 42 | 0.4144 | 1.2293 | 0.2207 | 0.9737 |
| PID_AR_PATHWAY | 61 | 0.4184 | 1.2153 | 0.2857 | 0.8977 |
| PID_BETA_CATENIN_DEG_PATHWAY | 18 | 0.4725 | 1.1639 | 0.3256 | 0.9646 |
| PID_HDAC_CLASSIII_PATHWAY | 26 | 0.3941 | 1.1625 | 0.2515 | 0.8719 |
| PID_PS1_PATHWAY | 45 | 0.4020 | 1.1609 | 0.2932 | 0.7972 |
| PID_SMAD2_3PATHWAY | 16 | 0.5194 | 1.1569 | 0.3469 | 0.7406 |
| PID_IL1_PATHWAY | 34 | 0.4204 | 1.1236 | 0.3347 | 0.7690 |
| PID_ANGIOPOIETIN_RECEPTOR_PATHWAY | 49 | 0.3634 | 1.0637 | 0.4170 | 0.8516 |
| PID_ERA_GENOMIC_PATHWAY | 64 | 0.2970 | 1.0299 | 0.3950 | 0.8799 |
| PID_HIF2PATHWAY | 34 | 0.3353 | 1.0256 | 0.4104 | 0.8347 |
| PID_EPO_PATHWAY | 33 | 0.3750 | 1.0254 | 0.4583 | 0.7862 |
| PID_PDGFRA_PATHWAY | 22 | 0.3797 | 1.0002 | 0.4625 | 0.7958 |
| PID_RET_PATHWAY | 39 | 0.3489 | 0.9930 | 0.4691 | 0.7693 |
| PID_IGF1_PATHWAY | 29 | 0.3554 | 0.9808 | 0.5020 | 0.7589 |
| PID_NFKAPPAB_CANONICAL_PATHWAY | 23 | 0.4000 | 0.9764 | 0.5202 | 0.7334 |
| PID_SHP2_PATHWAY | 57 | 0.3230 | 0.9760 | 0.4774 | 0.7008 |
| PID_S1P_S1P2_PATHWAY | 24 | 0.3642 | 0.9669 | 0.5154 | 0.6886 |
| PID_BETA_CATENIN_NUC_PATHWAY | 78 | 0.2954 | 0.9591 | 0.5050 | 0.6739 |
| PID_HEDGEHOG_2PATHWAY | 22 | 0.3481 | 0.9532 | 0.5179 | 0.6570 |
| PID_RXR_VDR_PATHWAY | 26 | 0.3221 | 0.9301 | 0.5539 | 0.6727 |
| PID_CIRCADIAN_PATHWAY | 16 | 0.3796 | 0.9300 | 0.5952 | 0.6478 |
| PID_LYMPH_ANGIOGENESIS_PATHWAY | 25 | 0.3549 | 0.9141 | 0.5738 | 0.6540 |
| PID_CD40_PATHWAY | 31 | 0.3180 | 0.8985 | 0.5560 | 0.6573 |
| PID_IFNG_PATHWAY | 40 | 0.3204 | 0.8769 | 0.5907 | 0.6737 |
| PID_AJDISS_2PATHWAY | 48 | 0.2934 | 0.8726 | 0.6008 | 0.6582 |
| PID_E2F_PATHWAY | 73 | 0.3188 | 0.8685 | 0.6017 | 0.6451 |
| PID_P53_REGULATION_PATHWAY | 59 | 0.3146 | 0.8679 | 0.6292 | 0.6266 |
| PID_IL2_1PATHWAY | 55 | 0.3046 | 0.8630 | 0.6183 | 0.6159 |
| PID_HEDGEHOG_GLI_PATHWAY | 48 | 0.3002 | 0.8408 | 0.6277 | 0.6307 |
| PID_FAS_PATHWAY | 38 | 0.2962 | 0.8304 | 0.6373 | 0.6304 |
| PID_SMAD2_3NUCLEAR_PATHWAY | 82 | 0.2801 | 0.8303 | 0.6758 | 0.6137 |
| PID_ERBB1_INTERNALIZATION_PATHWAY | 41 | 0.2770 | 0.7572 | 0.7444 | 0.7167 |
| PID_EPHA2_FWD_PATHWAY | 19 | 0.2963 | 0.7532 | 0.7602 | 0.7043 |
| PID_AURORA_B_PATHWAY | 39 | 0.2916 | 0.7277 | 0.7067 | 0.7251 |
| PID_IL2_STAT5_PATHWAY | 30 | 0.2640 | 0.6837 | 0.7760 | 0.7775 |
| PID_ATM_PATHWAY | 34 | 0.2759 | 0.6720 | 0.7702 | 0.7762 |
| PID_IL2_PI3K_PATHWAY | 34 | 0.2151 | 0.5995 | 0.9066 | 0.8626 |
| PID_HIF1A_PATHWAY | 19 | -0.6144 | -1.6547 | 0.0095 | 1.0000 |
| PID_FRA_PATHWAY | 37 | -0.5760 | -1.6052 | 0.0312 | 1.0000 |
| PID_SYNDECAN_1_PATHWAY | 46 | -0.5893 | -1.5970 | 0.0318 | 0.8925 |
| PID_RAS_PATHWAY | 30 | -0.5624 | -1.5696 | 0.0400 | 0.8215 |
| PID_ALK1_PATHWAY | 26 | -0.5808 | -1.5518 | 0.0482 | 0.7501 |
| PID_ARF6_DOWNSTREAM_PATHWAY | 15 | -0.6369 | -1.5428 | 0.0403 | 0.6687 |
| PID_LIS1_PATHWAY | 28 | -0.5685 | -1.5292 | 0.0678 | 0.6358 |
| PID_SYNDECAN_3_PATHWAY | 17 | -0.5956 | -1.5194 | 0.0430 | 0.5948 |
| PID_INSULIN_GLUCOSE_PATHWAY | 26 | -0.5457 | -1.5104 | 0.0493 | 0.5673 |
| PID_RAC1_PATHWAY | 54 | -0.5402 | -1.5067 | 0.0586 | 0.5237 |
| PID_TAP63_PATHWAY | 54 | -0.4534 | -1.4750 | 0.0740 | 0.5995 |
| PID_TCR_CALCIUM_PATHWAY | 28 | -0.5553 | -1.4713 | 0.0835 | 0.5620 |
| PID_INTEGRIN3_PATHWAY | 42 | -0.5102 | -1.4661 | 0.0865 | 0.5358 |
| PID_AP1_PATHWAY | 69 | -0.4569 | -1.4582 | 0.0584 | 0.5224 |
| PID_CERAMIDE_PATHWAY | 44 | -0.4809 | -1.4476 | 0.0968 | 0.5177 |
| PID_ALPHA_SYNUCLEIN_PATHWAY | 32 | -0.4998 | -1.4281 | 0.0888 | 0.5403 |
| PID_INTEGRIN_CS_PATHWAY | 26 | -0.5334 | -1.4249 | 0.1340 | 0.5193 |
| PID_P38_MK2_PATHWAY | 21 | -0.5492 | -1.4136 | 0.1288 | 0.5212 |
| PID_PRL_SIGNALING_EVENTS_PATHWAY | 23 | -0.5027 | -1.4055 | 0.0933 | 0.5136 |
| PID_INTEGRIN2_PATHWAY | 28 | -0.5195 | -1.3987 | 0.1168 | 0.5093 |
| PID_WNT_SIGNALING_PATHWAY | 28 | -0.4752 | -1.3890 | 0.0886 | 0.5107 |
| PID_TXA2PATHWAY | 56 | -0.4899 | -1.3660 | 0.1569 | 0.5554 |
| PID_P38_ALPHA_BETA_DOWNSTREAM_PATHWAY | 38 | -0.4612 | -1.3634 | 0.1317 | 0.5372 |
| PID_ECADHERIN_STABILIZATION_PATHWAY | 41 | -0.4878 | -1.3596 | 0.1351 | 0.5249 |
| PID_INTEGRIN_A9B1_PATHWAY | 25 | -0.4886 | -1.3486 | 0.1310 | 0.5366 |
| PID_IL8_CXCR2_PATHWAY | 34 | -0.5244 | -1.3425 | 0.1827 | 0.5318 |
| PID_ARF6_PATHWAY | 35 | -0.4563 | -1.3374 | 0.1277 | 0.5247 |
| PID_RHOA_REG_PATHWAY | 46 | -0.4807 | -1.3352 | 0.1853 | 0.5104 |
| PID_UPA_UPAR_PATHWAY | 42 | -0.4461 | -1.3107 | 0.1616 | 0.5508 |
| PID_IL8_CXCR1_PATHWAY | 28 | -0.5166 | -1.3068 | 0.2000 | 0.5422 |
| PID_ARF_3PATHWAY | 19 | -0.5290 | -1.2851 | 0.2112 | 0.5769 |
| PID_PDGFRB_PATHWAY | 129 | -0.4298 | -1.2808 | 0.2117 | 0.5686 |
| PID_NOTCH_PATHWAY | 59 | -0.4358 | -1.2786 | 0.2254 | 0.5576 |
| PID_ILK_PATHWAY | 45 | -0.4563 | -1.2659 | 0.2257 | 0.5706 |
| PID_LKB1_PATHWAY | 47 | -0.4270 | -1.2656 | 0.2158 | 0.5552 |
| PID_ENDOTHELIN_PATHWAY | 63 | -0.4292 | -1.2650 | 0.2070 | 0.5408 |
| PID_TOLL_ENDOGENOUS_PATHWAY | 24 | -0.4494 | -1.2505 | 0.1946 | 0.5559 |
| PID_ERBB1_DOWNSTREAM_PATHWAY | 105 | -0.4062 | -1.2444 | 0.2359 | 0.5563 |
| PID_CXCR4_PATHWAY | 101 | -0.4349 | -1.2442 | 0.2520 | 0.5424 |
| PID_EPHRINB_REV_PATHWAY | 30 | -0.4362 | -1.2422 | 0.2150 | 0.5325 |
| PID_INTEGRIN5_PATHWAY | 16 | -0.4927 | -1.2392 | 0.2088 | 0.5251 |
| PID_PI3KCI_PATHWAY | 48 | -0.4543 | -1.2385 | 0.2628 | 0.5148 |
| PID_P53_DOWNSTREAM_PATHWAY | 137 | -0.3616 | -1.2362 | 0.2004 | 0.5074 |
| PID_NETRIN_PATHWAY | 32 | -0.4652 | -1.2334 | 0.2540 | 0.5008 |
| PID_INTEGRIN1_PATHWAY | 66 | -0.4214 | -1.2294 | 0.2539 | 0.4977 |
| PID_NEPHRIN_NEPH1_PATHWAY | 31 | -0.4422 | -1.2290 | 0.2530 | 0.4878 |
| PID_ANTHRAX_PATHWAY | 18 | -0.4822 | -1.2243 | 0.2500 | 0.4854 |
| PID_AURORA_A_PATHWAY | 31 | -0.4612 | -1.2241 | 0.2495 | 0.4755 |
| PID_RHOA_PATHWAY | 44 | -0.4309 | -1.2231 | 0.2406 | 0.4677 |
| PID_CASPASE_PATHWAY | 51 | -0.4157 | -1.2102 | 0.2642 | 0.4822 |
| PID_DELTA_NP63_PATHWAY | 47 | -0.3724 | -1.2072 | 0.2354 | 0.4778 |
| PID_ERBB2_ERBB3_PATHWAY | 44 | -0.4140 | -1.2048 | 0.2613 | 0.4725 |
| PID_NFAT_TFPATHWAY | 45 | -0.4262 | -1.2038 | 0.2663 | 0.4650 |
| PID_SYNDECAN_2_PATHWAY | 33 | -0.4061 | -1.2010 | 0.2648 | 0.4612 |
| PID_IL6_7_PATHWAY | 47 | -0.3971 | -1.1968 | 0.2395 | 0.4604 |
| PID_ATR_PATHWAY | 39 | -0.4936 | -1.1881 | 0.3512 | 0.4681 |
| PID_ERB_GENOMIC_PATHWAY | 15 | -0.4370 | -1.1833 | 0.2630 | 0.4682 |
| PID_NECTIN_PATHWAY | 30 | -0.4566 | -1.1828 | 0.3016 | 0.4612 |
| PID_P38_MKK3_6PATHWAY | 26 | -0.4006 | -1.1792 | 0.2651 | 0.4588 |
| PID_LYSOPHOSPHOLIPID_PATHWAY | 65 | -0.3874 | -1.1646 | 0.3074 | 0.4754 |
| PID_P75_NTR_PATHWAY | 68 | -0.3665 | -1.1614 | 0.2819 | 0.4728 |
| PID_TRKR_PATHWAY | 61 | -0.3868 | -1.1450 | 0.3135 | 0.4951 |
| PID_CDC42_PATHWAY | 70 | -0.3849 | -1.1346 | 0.3400 | 0.5047 |
| PID_A6B1_A6B4_INTEGRIN_PATHWAY | 46 | -0.3711 | -1.1241 | 0.3006 | 0.5140 |
| PID_P38_ALPHA_BETA_PATHWAY | 30 | -0.3899 | -1.1121 | 0.3550 | 0.5295 |
| PID_ATF2_PATHWAY | 59 | -0.3454 | -1.1097 | 0.3359 | 0.5258 |
| PID_SYNDECAN_4_PATHWAY | 32 | -0.3654 | -1.1079 | 0.3250 | 0.5220 |
| PID_HIF1_TFPATHWAY | 66 | -0.3455 | -1.1062 | 0.3346 | 0.5170 |
| PID_EPHA_FWDPATHWAY | 34 | -0.3910 | -1.1015 | 0.3467 | 0.5173 |
| PID_S1P_S1P3_PATHWAY | 29 | -0.4113 | -1.0918 | 0.3857 | 0.5257 |
| PID_INTEGRIN_A4B1_PATHWAY | 33 | -0.3805 | -1.0879 | 0.3837 | 0.5244 |
| PID_ECADHERIN_KERATINOCYTE_PATHWAY | 21 | -0.4280 | -1.0846 | 0.4094 | 0.5226 |
| PID_GMCSF_PATHWAY | 36 | -0.3884 | -1.0801 | 0.3730 | 0.5225 |
| PID_CXCR3_PATHWAY | 43 | -0.3706 | -1.0766 | 0.3675 | 0.5212 |
| PID_IL23_PATHWAY | 37 | -0.3920 | -1.0683 | 0.3961 | 0.5292 |
| PID_GLYPICAN_1PATHWAY | 27 | -0.4035 | -1.0671 | 0.4052 | 0.5262 |
| PID_WNT_NONCANONICAL_PATHWAY | 32 | -0.3968 | -1.0649 | 0.4370 | 0.5226 |
| PID_HDAC_CLASSII_PATHWAY | 34 | -0.3738 | -1.0639 | 0.3848 | 0.5178 |
| PID_P73PATHWAY | 79 | -0.3287 | -1.0635 | 0.3907 | 0.5116 |
| PID_IL12_STAT4_PATHWAY | 33 | -0.4257 | -1.0606 | 0.4353 | 0.5091 |
| PID_HES_HEY_PATHWAY | 48 | -0.3614 | -1.0532 | 0.4212 | 0.5181 |
| PID_S1P_S1P1_PATHWAY | 21 | -0.4016 | -1.0469 | 0.4255 | 0.5204 |
| PID_HDAC_CLASSI_PATHWAY | 66 | -0.3447 | -1.0464 | 0.4222 | 0.5149 |
| PID_MTOR_4PATHWAY | 69 | -0.3436 | -1.0443 | 0.4095 | 0.5118 |
| PID_VEGFR1_2_PATHWAY | 69 | -0.3531 | -1.0386 | 0.4477 | 0.5163 |
| PID_AVB3_INTEGRIN_PATHWAY | 74 | -0.3563 | -1.0368 | 0.4420 | 0.5132 |
| PID_ER_NONGENOMIC_PATHWAY | 40 | -0.3652 | -1.0341 | 0.4172 | 0.5114 |
| PID_LPA4_PATHWAY | 15 | -0.4191 | -1.0336 | 0.4375 | 0.5067 |
| PID_CD8_TCR_DOWNSTREAM_PATHWAY | 65 | -0.3541 | -1.0327 | 0.4334 | 0.5026 |
| PID_ERBB_NETWORK_PATHWAY | 15 | -0.4177 | -1.0309 | 0.4526 | 0.4995 |
| PID_IL4_2PATHWAY | 64 | -0.3328 | -1.0308 | 0.4218 | 0.4940 |
| PID_FOXM1_PATHWAY | 40 | -0.3887 | -1.0307 | 0.4425 | 0.4888 |
| PID_REELIN_PATHWAY | 28 | -0.3850 | -1.0163 | 0.4462 | 0.5063 |
| PID_IL27_PATHWAY | 26 | -0.3856 | -1.0149 | 0.4382 | 0.5027 |
| PID_AMB2_NEUTROPHILS_PATHWAY | 40 | -0.3665 | -1.0118 | 0.4589 | 0.5015 |
| PID_MAPK_TRK_PATHWAY | 34 | -0.3722 | -1.0116 | 0.4659 | 0.4965 |
| PID_S1P_META_PATHWAY | 21 | -0.3758 | -0.9940 | 0.4567 | 0.5172 |
| PID_FCER1_PATHWAY | 60 | -0.3494 | -0.9934 | 0.4943 | 0.5127 |
| PID_PI3KCI_AKT_PATHWAY | 35 | -0.3479 | -0.9904 | 0.4673 | 0.5114 |
| PID_THROMBIN_PAR1_PATHWAY | 43 | -0.3480 | -0.9886 | 0.4711 | 0.5086 |
| PID_EPHB_FWD_PATHWAY | 40 | -0.3510 | -0.9854 | 0.4870 | 0.5076 |
| PID_KIT_PATHWAY | 52 | -0.3485 | -0.9853 | 0.5000 | 0.5029 |
| PID_AR_NONGENOMIC_PATHWAY | 31 | -0.3350 | -0.9837 | 0.4922 | 0.4999 |
| PID_REG_GR_PATHWAY | 82 | -0.2881 | -0.9763 | 0.4915 | 0.5052 |
| PID_ARF6_TRAFFICKING_PATHWAY | 49 | -0.3344 | -0.9753 | 0.4901 | 0.5017 |
| PID_RB_1PATHWAY | 65 | -0.3200 | -0.9582 | 0.5110 | 0.5221 |
| PID_CDC42_REG_PATHWAY | 30 | -0.3695 | -0.9552 | 0.5474 | 0.5228 |
| PID_NFKAPPAB_ATYPICAL_PATHWAY | 17 | -0.3681 | -0.9435 | 0.5500 | 0.5349 |
| PID_TNF_PATHWAY | 46 | -0.3294 | -0.9435 | 0.5475 | 0.5302 |
| PID_PTP1B_PATHWAY | 52 | -0.3194 | -0.9425 | 0.5300 | 0.5268 |
| PID_NFAT_3PATHWAY | 53 | -0.3316 | -0.9350 | 0.5580 | 0.5340 |
| PID_RAC1_REG_PATHWAY | 38 | -0.3335 | -0.9313 | 0.5383 | 0.5332 |
| PID_ERBB1_RECEPTOR_PROXIMAL_PATHWAY | 35 | -0.3376 | -0.9025 | 0.5788 | 0.5684 |
| PID_PI3K_PLC_TRK_PATHWAY | 36 | -0.3185 | -0.8986 | 0.6027 | 0.5693 |
| PID_AVB3_OPN_PATHWAY | 31 | -0.3344 | -0.8957 | 0.5843 | 0.5684 |
| PID_MYC_REPRESS_PATHWAY | 63 | -0.2681 | -0.8928 | 0.5984 | 0.5671 |
| PID_WNT_CANONICAL_PATHWAY | 20 | -0.3440 | -0.8795 | 0.5927 | 0.5854 |
| PID_INSULIN_PATHWAY | 44 | -0.3157 | -0.8789 | 0.6282 | 0.5814 |
| PID_MYC_ACTIV_PATHWAY | 79 | -0.2983 | -0.8771 | 0.5889 | 0.5796 |
| PID_TRAIL_PATHWAY | 28 | -0.3178 | -0.8756 | 0.6089 | 0.5768 |
| PID_ECADHERIN_NASCENT_AJ_PATHWAY | 39 | -0.3190 | -0.8739 | 0.6245 | 0.5750 |
| PID_IL3_PATHWAY | 26 | -0.3029 | -0.8615 | 0.6287 | 0.5887 |
| PID_CMYB_PATHWAY | 84 | -0.2629 | -0.8611 | 0.6534 | 0.5848 |
| PID_MET_PATHWAY | 79 | -0.2946 | -0.8578 | 0.6353 | 0.5847 |
| PID_IL12_2PATHWAY | 63 | -0.3196 | -0.8578 | 0.5927 | 0.5800 |
| PID_FAK_PATHWAY | 59 | -0.3164 | -0.8569 | 0.6252 | 0.5765 |
| PID_TGFBR_PATHWAY | 53 | -0.2988 | -0.8381 | 0.6612 | 0.5981 |
| PID_THROMBIN_PAR4_PATHWAY | 15 | -0.3451 | -0.8369 | 0.6693 | 0.5950 |
| PID_NCADHERIN_PATHWAY | 33 | -0.3127 | -0.8258 | 0.6407 | 0.6066 |
| PID_FGF_PATHWAY | 55 | -0.2649 | -0.8231 | 0.7002 | 0.6056 |
| PID_TCR_PATHWAY | 65 | -0.3221 | -0.8135 | 0.6511 | 0.6149 |
| PID_RHODOPSIN_PATHWAY | 23 | -0.2665 | -0.8050 | 0.7611 | 0.6213 |
| PID_MYC_PATHWAY | 25 | -0.2989 | -0.7763 | 0.6841 | 0.6579 |
| PID_TCPTP_PATHWAY | 42 | -0.2742 | -0.7733 | 0.7318 | 0.6582 |
| PID_CONE_PATHWAY | 22 | -0.2592 | -0.7539 | 0.8098 | 0.6821 |
| PID_DNA_PK_PATHWAY | 16 | -0.2991 | -0.7492 | 0.7525 | 0.6838 |
| PID_BCR_5PATHWAY | 63 | -0.2579 | -0.7172 | 0.7547 | 0.7297 |
| PID_VEGFR1_PATHWAY | 26 | -0.2752 | -0.7072 | 0.7588 | 0.7409 |
| PID_TELOMERASE_PATHWAY | 67 | -0.2405 | -0.6911 | 0.7806 | 0.7600 |
| PID_HIV_NEF_PATHWAY | 35 | -0.2450 | -0.6859 | 0.8121 | 0.7626 |
| PID_CD8_TCR_PATHWAY | 53 | -0.2649 | -0.6686 | 0.7765 | 0.7847 |
| PID_BARD1_PATHWAY | 29 | -0.2792 | -0.6610 | 0.7843 | 0.7911 |
| PID_FANCONI_PATHWAY | 47 | -0.2656 | -0.6425 | 0.8055 | 0.8110 |
| PID_PLK1_PATHWAY | 46 | -0.2502 | -0.6186 | 0.7885 | 0.8382 |

| **Signaling pathways enriched in HCC samples corresponding ADH6 expression by GSEA based on PID** | | | | | |
| --- | --- | --- | --- | --- | --- |
| **PID Name** | **Size** | **ES** | **NES** | **NOM p-value** | **FDR q-value** |
| PID_HNF3B_PATHWAY | 45 | 0.4976 | 1.6226 | 0.0141 | 0.0328 |
| PID_HNF3A_PATHWAY | 44 | 0.4580 | 1.5044 | 0.0359 | 0.0409 |
| PID_IL8_CXCR2_PATHWAY | 34 | -0.7530 | -1.9456 | 0.0000 | 0.0298 |
| PID_WNT_SIGNALING_PATHWAY | 28 | -0.6608 | -1.9370 | 0.0000 | 0.0176 |
| PID_RAC1_PATHWAY | 54 | -0.6987 | -1.9298 | 0.0000 | 0.0143 |
| PID_IL8_CXCR1_PATHWAY | 28 | -0.7275 | -1.8876 | 0.0020 | 0.0246 |
| PID_INSULIN_GLUCOSE_PATHWAY | 26 | -0.6736 | -1.8765 | 0.0079 | 0.0234 |
| PID_RHOA_PATHWAY | 44 | -0.6535 | -1.8447 | 0.0000 | 0.0343 |
| PID_CASPASE_PATHWAY | 51 | -0.6272 | -1.8269 | 0.0000 | 0.0419 |
| PID_P38_MK2_PATHWAY | 21 | -0.7331 | -1.8234 | 0.0000 | 0.0386 |
| PID_P53_DOWNSTREAM_PATHWAY | 137 | -0.5401 | -1.8140 | 0.0000 | 0.0386 |
| PID_CXCR4_PATHWAY | 101 | -0.6272 | -1.8104 | 0.0081 | 0.0362 |
| PID_PDGFRB_PATHWAY | 129 | -0.6114 | -1.8066 | 0.0039 | 0.0351 |
| PID_PI3KCI_PATHWAY | 48 | -0.6658 | -1.8008 | 0.0041 | 0.0347 |
| PID_RHOA_REG_PATHWAY | 46 | -0.6606 | -1.7912 | 0.0020 | 0.0360 |
| PID_ERBB1_DOWNSTREAM_PATHWAY | 105 | -0.5984 | -1.7850 | 0.0155 | 0.0351 |
| PID_ERBB2_ERBB3_PATHWAY | 44 | -0.6196 | -1.7801 | 0.0059 | 0.0350 |
| PID_FOXM1_PATHWAY | 40 | -0.6768 | -1.7719 | 0.0123 | 0.0367 |
| PID_NOTCH_PATHWAY | 59 | -0.6065 | -1.7640 | 0.0040 | 0.0376 |
| PID_RAS_PATHWAY | 30 | -0.6249 | -1.7469 | 0.0080 | 0.0438 |
| PID_MAPK_TRK_PATHWAY | 34 | -0.6494 | -1.7457 | 0.0061 | 0.0419 |
| PID_CDC42_PATHWAY | 70 | -0.5977 | -1.7454 | 0.0098 | 0.0398 |
| PID_ATR_PATHWAY | 39 | -0.7254 | -1.7403 | 0.0080 | 0.0404 |
| PID_P38_ALPHA_BETA_DOWNSTREAM_PATHWAY | 38 | -0.6053 | -1.7370 | 0.0080 | 0.0401 |
| PID_ILK_PATHWAY | 45 | -0.6433 | -1.7346 | 0.0080 | 0.0390 |
| PID_ARF6_PATHWAY | 35 | -0.5791 | -1.7285 | 0.0097 | 0.0401 |
| PID_LKB1_PATHWAY | 47 | -0.5976 | -1.7238 | 0.0118 | 0.0405 |
| PID_PRL_SIGNALING_EVENTS_PATHWAY | 23 | -0.6263 | -1.7176 | 0.0060 | 0.0414 |
| PID_TXA2PATHWAY | 56 | -0.6107 | -1.7176 | 0.0165 | 0.0398 |
| PID_FCER1_PATHWAY | 60 | -0.6070 | -1.7167 | 0.0138 | 0.0389 |
| PID_ARF6_DOWNSTREAM_PATHWAY | 15 | -0.7078 | -1.7135 | 0.0061 | 0.0388 |
| PID_CERAMIDE_PATHWAY | 44 | -0.5894 | -1.7105 | 0.0160 | 0.0389 |
| PID_INTEGRIN_A9B1_PATHWAY | 25 | -0.6121 | -1.7056 | 0.0102 | 0.0395 |
| PID_CXCR3_PATHWAY | 43 | -0.5779 | -1.7055 | 0.0101 | 0.0383 |
| PID_NEPHRIN_NEPH1_PATHWAY | 31 | -0.6249 | -1.7047 | 0.0079 | 0.0373 |
| PID_P38_ALPHA_BETA_PATHWAY | 30 | -0.6075 | -1.7032 | 0.0143 | 0.0367 |
| PID_DELTA_NP63_PATHWAY | 47 | -0.5447 | -1.7028 | 0.0041 | 0.0358 |
| PID_KIT_PATHWAY | 52 | -0.6110 | -1.7002 | 0.0081 | 0.0357 |
| PID_MYC_PATHWAY | 25 | -0.6515 | -1.6992 | 0.0060 | 0.0350 |
| PID_ARF_3PATHWAY | 19 | -0.7035 | -1.6920 | 0.0061 | 0.0383 |
| PID_TRKR_PATHWAY | 61 | -0.5728 | -1.6815 | 0.0161 | 0.0414 |
| PID_RB_1PATHWAY | 65 | -0.5611 | -1.6802 | 0.0119 | 0.0408 |
| PID_INTEGRIN_CS_PATHWAY | 26 | -0.6535 | -1.6798 | 0.0142 | 0.0399 |
| PID_P73PATHWAY | 79 | -0.5178 | -1.6759 | 0.0136 | 0.0402 |
| PID_WNT_NONCANONICAL_PATHWAY | 32 | -0.6198 | -1.6719 | 0.0234 | 0.0410 |
| PID_AR_NONGENOMIC_PATHWAY | 31 | -0.5750 | -1.6701 | 0.0176 | 0.0408 |
| PID_ERB_GENOMIC_PATHWAY | 15 | -0.6323 | -1.6701 | 0.0104 | 0.0399 |
| PID_BCR_5PATHWAY | 63 | -0.6135 | -1.6690 | 0.0103 | 0.0395 |
| PID_PLK1_PATHWAY | 46 | -0.6849 | -1.6686 | 0.0277 | 0.0387 |
| PID_SYNDECAN_1_PATHWAY | 46 | -0.6148 | -1.6666 | 0.0299 | 0.0388 |
| PID_EPHA_FWDPATHWAY | 34 | -0.5885 | -1.6648 | 0.0161 | 0.0387 |
| PID_CD8_TCR_DOWNSTREAM_PATHWAY | 65 | -0.5783 | -1.6602 | 0.0271 | 0.0400 |
| PID_ERBB1_INTERNALIZATION_PATHWAY | 41 | -0.6080 | -1.6572 | 0.0254 | 0.0406 |
| PID_TELOMERASE_PATHWAY | 67 | -0.5837 | -1.6502 | 0.0250 | 0.0425 |
| PID_PI3KCI_AKT_PATHWAY | 35 | -0.5896 | -1.6490 | 0.0160 | 0.0422 |
| PID_BARD1_PATHWAY | 29 | -0.6894 | -1.6483 | 0.0270 | 0.0417 |
| PID_GMCSF_PATHWAY | 36 | -0.5912 | -1.6437 | 0.0196 | 0.0424 |
| PID_PI3K_PLC_TRK_PATHWAY | 36 | -0.5887 | -1.6430 | 0.0281 | 0.0418 |
| PID_PTP1B_PATHWAY | 52 | -0.5535 | -1.6405 | 0.0236 | 0.0421 |
| PID_ALPHA_SYNUCLEIN_PATHWAY | 32 | -0.5760 | -1.6400 | 0.0223 | 0.0415 |
| PID_HDAC_CLASSI_PATHWAY | 66 | -0.5548 | -1.6388 | 0.0199 | 0.0414 |
| PID_RAC1_REG_PATHWAY | 38 | -0.5883 | -1.6375 | 0.0315 | 0.0412 |
| PID_ATM_PATHWAY | 34 | -0.6730 | -1.6312 | 0.0120 | 0.0432 |
| PID_TCR_PATHWAY | 65 | -0.6382 | -1.6279 | 0.0373 | 0.0437 |
| PID_CD8_TCR_PATHWAY | 53 | -0.6486 | -1.6258 | 0.0377 | 0.0438 |
| PID_TCPTP_PATHWAY | 42 | -0.5783 | -1.6241 | 0.0362 | 0.0440 |
| PID_NETRIN_PATHWAY | 32 | -0.6172 | -1.6213 | 0.0292 | 0.0445 |
| PID_EPHB_FWD_PATHWAY | 40 | -0.5926 | -1.6195 | 0.0354 | 0.0447 |
| PID_TAP63_PATHWAY | 54 | -0.5214 | -1.6188 | 0.0203 | 0.0442 |
| PID_FANCONI_PATHWAY | 47 | -0.6702 | -1.6188 | 0.0339 | 0.0436 |
| PID_S1P_S1P3_PATHWAY | 29 | -0.6157 | -1.6173 | 0.0258 | 0.0436 |
| PID_MET_PATHWAY | 79 | -0.5664 | -1.6100 | 0.0329 | 0.0460 |
| PID_EPHA2_FWD_PATHWAY | 19 | -0.6259 | -1.6077 | 0.0312 | 0.0463 |
| PID_SYNDECAN_2_PATHWAY | 33 | -0.5602 | -1.6023 | 0.0234 | 0.0480 |
| PID_LIS1_PATHWAY | 28 | -0.6046 | -1.6023 | 0.0262 | 0.0473 |
| PID_MTOR_4PATHWAY | 69 | -0.5483 | -1.6022 | 0.0250 | 0.0467 |
| PID_ENDOTHELIN_PATHWAY | 63 | -0.5427 | -1.6020 | 0.0395 | 0.0461 |
| PID_EPHRINB_REV_PATHWAY | 30 | -0.5657 | -1.6011 | 0.0277 | 0.0460 |
| PID_HEDGEHOG_GLI_PATHWAY | 48 | -0.5745 | -1.5971 | 0.0410 | 0.0468 |
| PID_LYSOPHOSPHOLIPID_PATHWAY | 65 | -0.5323 | -1.5930 | 0.0355 | 0.0478 |
| PID_IL6_7_PATHWAY | 47 | -0.5299 | -1.5737 | 0.0406 | 0.0554 |
| PID_A6B1_A6B4_INTEGRIN_PATHWAY | 46 | -0.5450 | -1.5735 | 0.0559 | 0.0549 |
| PID_AURORA_A_PATHWAY | 31 | -0.5920 | -1.5714 | 0.0458 | 0.0552 |
| PID_HDAC_CLASSII_PATHWAY | 34 | -0.5658 | -1.5599 | 0.0290 | 0.0598 |
| PID_THROMBIN_PAR1_PATHWAY | 43 | -0.5418 | -1.5589 | 0.0697 | 0.0597 |
| PID_S1P_S1P1_PATHWAY | 21 | -0.6046 | -1.5583 | 0.0403 | 0.0592 |
| PID_ARF6_TRAFFICKING_PATHWAY | 49 | -0.5473 | -1.5576 | 0.0659 | 0.0588 |
| PID_IL12_2PATHWAY | 63 | -0.5725 | -1.5572 | 0.0863 | 0.0590 |
| PID_FAS_PATHWAY | 38 | -0.5547 | -1.5553 | 0.0566 | 0.0589 |
| PID_NFAT_3PATHWAY | 53 | -0.5561 | -1.5546 | 0.0519 | 0.0585 |
| PID_HES_HEY_PATHWAY | 48 | -0.5186 | -1.5522 | 0.0454 | 0.0588 |
| PID_IL3_PATHWAY | 26 | -0.5335 | -1.5521 | 0.0502 | 0.0582 |
| PID_ERBB1_RECEPTOR_PROXIMAL_PATHWAY | 35 | -0.5973 | -1.5499 | 0.0298 | 0.0586 |
| PID_UPA_UPAR_PATHWAY | 42 | -0.5300 | -1.5495 | 0.0299 | 0.0581 |
| PID_ECADHERIN_STABILIZATION_PATHWAY | 41 | -0.5772 | -1.5487 | 0.0517 | 0.0578 |
| PID_GLYPICAN_1PATHWAY | 27 | -0.5773 | -1.5464 | 0.0543 | 0.0581 |
| PID_IL12_STAT4_PATHWAY | 33 | -0.6279 | -1.5455 | 0.0736 | 0.0579 |
| PID_ERBB_NETWORK_PATHWAY | 15 | -0.6374 | -1.5447 | 0.0362 | 0.0576 |
| PID_INTEGRIN3_PATHWAY | 42 | -0.5507 | -1.5432 | 0.0560 | 0.0575 |
| PID_SMAD2_3NUCLEAR_PATHWAY | 82 | -0.5109 | -1.5381 | 0.0442 | 0.0594 |
| PID_AURORA_B_PATHWAY | 39 | -0.6104 | -1.5359 | 0.1018 | 0.0597 |
| PID_SYNDECAN_3_PATHWAY | 17 | -0.5927 | -1.5354 | 0.0413 | 0.0593 |
| PID_ER_NONGENOMIC_PATHWAY | 40 | -0.5465 | -1.5286 | 0.0791 | 0.0618 |
| PID_SMAD2_3PATHWAY | 16 | -0.6821 | -1.5242 | 0.0421 | 0.0628 |
| PID_CD40_PATHWAY | 31 | -0.5543 | -1.5241 | 0.0730 | 0.0622 |
| PID_SYNDECAN_4_PATHWAY | 32 | -0.5072 | -1.5235 | 0.0500 | 0.0618 |
| PID_INTEGRIN5_PATHWAY | 16 | -0.6023 | -1.5218 | 0.0562 | 0.0620 |
| PID_MYC_REPRESS_PATHWAY | 63 | -0.4714 | -1.5200 | 0.0430 | 0.0622 |
| PID_VEGFR1_2_PATHWAY | 69 | -0.5298 | -1.5183 | 0.0696 | 0.0624 |
| PID_FAK_PATHWAY | 59 | -0.5657 | -1.5138 | 0.0838 | 0.0636 |
| PID_TNF_PATHWAY | 46 | -0.5271 | -1.5105 | 0.0737 | 0.0645 |
| PID_ANTHRAX_PATHWAY | 18 | -0.6054 | -1.5032 | 0.0587 | 0.0669 |
| PID_TGFBR_PATHWAY | 53 | -0.5362 | -1.5020 | 0.0669 | 0.0669 |
| PID_CMYB_PATHWAY | 84 | -0.4593 | -1.4998 | 0.0481 | 0.0673 |
| PID_P75_NTR_PATHWAY | 68 | -0.4809 | -1.4989 | 0.0528 | 0.0672 |
| PID_MYC_ACTIV_PATHWAY | 79 | -0.5022 | -1.4939 | 0.0819 | 0.0688 |
| PID_HIF1_TFPATHWAY | 66 | -0.4762 | -1.4920 | 0.0509 | 0.0690 |
| PID_AP1_PATHWAY | 69 | -0.4800 | -1.4916 | 0.0699 | 0.0686 |
| PID_IL4_2PATHWAY | 64 | -0.4808 | -1.4891 | 0.0736 | 0.0691 |
| PID_CDC42_REG_PATHWAY | 30 | -0.5801 | -1.4890 | 0.0619 | 0.0686 |
| PID_LYMPH_ANGIOGENESIS_PATHWAY | 25 | -0.5716 | -1.4847 | 0.0885 | 0.0701 |
| PID_REG_GR_PATHWAY | 82 | -0.4395 | -1.4813 | 0.0614 | 0.0711 |
| PID_E2F_PATHWAY | 73 | -0.5233 | -1.4768 | 0.1175 | 0.0725 |
| PID_TCR_CALCIUM_PATHWAY | 28 | -0.5420 | -1.4729 | 0.0855 | 0.0739 |
| PID_ALK1_PATHWAY | 26 | -0.5529 | -1.4723 | 0.0927 | 0.0736 |
| PID_ECADHERIN_NASCENT_AJ_PATHWAY | 39 | -0.5442 | -1.4713 | 0.0953 | 0.0735 |
| PID_NFAT_TFPATHWAY | 45 | -0.5237 | -1.4681 | 0.0918 | 0.0744 |
| PID_NECTIN_PATHWAY | 30 | -0.5767 | -1.4668 | 0.0895 | 0.0747 |
| PID_IL27_PATHWAY | 26 | -0.5632 | -1.4590 | 0.1109 | 0.0774 |
| PID_CIRCADIAN_PATHWAY | 16 | -0.5957 | -1.4555 | 0.0798 | 0.0784 |
| PID_FRA_PATHWAY | 37 | -0.5398 | -1.4496 | 0.1093 | 0.0803 |
| PID_IFNG_PATHWAY | 40 | -0.5266 | -1.4415 | 0.0921 | 0.0837 |
| PID_HIV_NEF_PATHWAY | 35 | -0.5220 | -1.4405 | 0.0784 | 0.0834 |
| PID_IL23_PATHWAY | 37 | -0.5321 | -1.4326 | 0.1189 | 0.0861 |
| PID_TRAIL_PATHWAY | 28 | -0.5154 | -1.4247 | 0.1174 | 0.0898 |
| PID_EPO_PATHWAY | 33 | -0.5222 | -1.4246 | 0.1016 | 0.0891 |
| PID_HIF1A_PATHWAY | 19 | -0.5322 | -1.4236 | 0.1064 | 0.0889 |
| PID_AVB3_INTEGRIN_PATHWAY | 74 | -0.5045 | -1.4193 | 0.1089 | 0.0903 |
| PID_IL2_STAT5_PATHWAY | 30 | -0.5526 | -1.4192 | 0.1381 | 0.0897 |
| PID_INTEGRIN_A4B1_PATHWAY | 33 | -0.4956 | -1.4101 | 0.1417 | 0.0939 |
| PID_IL2_1PATHWAY | 55 | -0.4898 | -1.3899 | 0.1426 | 0.1033 |
| PID_INSULIN_PATHWAY | 44 | -0.5072 | -1.3858 | 0.1403 | 0.1046 |
| PID_BETA_CATENIN_NUC_PATHWAY | 78 | -0.4323 | -1.3838 | 0.1220 | 0.1049 |
| PID_INTEGRIN1_PATHWAY | 66 | -0.4780 | -1.3724 | 0.1494 | 0.1103 |
| PID_P53_REGULATION_PATHWAY | 59 | -0.4984 | -1.3707 | 0.1641 | 0.1110 |
| PID_FOXO_PATHWAY | 49 | -0.4883 | -1.3668 | 0.1404 | 0.1124 |
| PID_ATF2_PATHWAY | 59 | -0.4260 | -1.3616 | 0.1278 | 0.1150 |
| PID_AMB2_NEUTROPHILS_PATHWAY | 40 | -0.4961 | -1.3558 | 0.1455 | 0.1171 |
| PID_NCADHERIN_PATHWAY | 33 | -0.5208 | -1.3540 | 0.1617 | 0.1172 |
| PID_AJDISS_2PATHWAY | 48 | -0.4598 | -1.3527 | 0.1533 | 0.1172 |
| PID_PS1_PATHWAY | 45 | -0.4677 | -1.3475 | 0.1634 | 0.1193 |
| PID_FGF_PATHWAY | 55 | -0.4412 | -1.3420 | 0.1135 | 0.1214 |
| PID_S1P_S1P2_PATHWAY | 24 | -0.5126 | -1.3417 | 0.1637 | 0.1207 |
| PID_VEGFR1_PATHWAY | 26 | -0.5287 | -1.3411 | 0.1800 | 0.1203 |
| PID_REELIN_PATHWAY | 28 | -0.5046 | -1.3407 | 0.1590 | 0.1197 |
| PID_IL2_PI3K_PATHWAY | 34 | -0.4916 | -1.3374 | 0.1565 | 0.1208 |
| PID_SHP2_PATHWAY | 57 | -0.4442 | -1.3345 | 0.1544 | 0.1214 |
| PID_AVB3_OPN_PATHWAY | 31 | -0.5115 | -1.3338 | 0.1713 | 0.1210 |
| PID_IGF1_PATHWAY | 29 | -0.4939 | -1.3322 | 0.1693 | 0.1211 |
| PID_TOLL_ENDOGENOUS_PATHWAY | 24 | -0.4864 | -1.3287 | 0.1449 | 0.1223 |
| PID_RET_PATHWAY | 39 | -0.4706 | -1.3176 | 0.1732 | 0.1280 |
| PID_WNT_CANONICAL_PATHWAY | 20 | -0.5215 | -1.3153 | 0.1804 | 0.1283 |
| PID_ANGIOPOIETIN_RECEPTOR_PATHWAY | 49 | -0.4443 | -1.3080 | 0.1942 | 0.1312 |
| PID_ECADHERIN_KERATINOCYTE_PATHWAY | 21 | -0.5191 | -1.3002 | 0.1811 | 0.1356 |
| PID_S1P_META_PATHWAY | 21 | -0.4874 | -1.2942 | 0.1935 | 0.1380 |
| PID_AR_PATHWAY | 61 | -0.4477 | -1.2893 | 0.2144 | 0.1408 |
| PID_CONE_PATHWAY | 22 | -0.4350 | -1.2774 | 0.1525 | 0.1477 |
| PID_INTEGRIN2_PATHWAY | 28 | -0.4734 | -1.2733 | 0.1915 | 0.1489 |
| PID_NFKAPPAB_ATYPICAL_PATHWAY | 17 | -0.4996 | -1.2686 | 0.2052 | 0.1506 |
| PID_NFKAPPAB_CANONICAL_PATHWAY | 23 | -0.5118 | -1.2623 | 0.2353 | 0.1539 |
| PID_P38_MKK3_6PATHWAY | 26 | -0.4346 | -1.2496 | 0.2056 | 0.1606 |
| PID_HIF2PATHWAY | 34 | -0.4149 | -1.2437 | 0.2191 | 0.1633 |
| PID_DNA_PK_PATHWAY | 16 | -0.5004 | -1.2431 | 0.2578 | 0.1630 |
| PID_THROMBIN_PAR4_PATHWAY | 15 | -0.5052 | -1.2424 | 0.2454 | 0.1623 |
| PID_ERBB4_PATHWAY | 38 | -0.4118 | -1.2087 | 0.2599 | 0.1829 |
| PID_RXR_VDR_PATHWAY | 26 | -0.4137 | -1.1681 | 0.2871 | 0.2099 |
| PID_PDGFRA_PATHWAY | 22 | -0.4300 | -1.1479 | 0.3487 | 0.2236 |
| PID_ERA_GENOMIC_PATHWAY | 64 | -0.3333 | -1.1473 | 0.2838 | 0.2227 |
| PID_LPA4_PATHWAY | 15 | -0.4288 | -1.0697 | 0.4016 | 0.2826 |
| PID_BMP_PATHWAY | 42 | -0.3603 | -1.0646 | 0.4012 | 0.2851 |
| PID_BETA_CATENIN_DEG_PATHWAY | 18 | -0.4171 | -1.0437 | 0.4359 | 0.3017 |
| PID_RHODOPSIN_PATHWAY | 23 | -0.3362 | -1.0214 | 0.4232 | 0.3212 |
| PID_IL1_PATHWAY | 34 | -0.3714 | -1.0213 | 0.4535 | 0.3196 |
| PID_HDAC_CLASSIII_PATHWAY | 26 | -0.3438 | -1.0051 | 0.4512 | 0.3316 |
| PID_HEDGEHOG_2PATHWAY | 22 | -0.3565 | -0.9878 | 0.4627 | 0.3466 |
| PID_RETINOIC_ACID_PATHWAY | 30 | -0.3540 | -0.9846 | 0.4892 | 0.3482 |
| PID_AR_TF_PATHWAY | 53 | -0.3295 | -0.9484 | 0.5372 | 0.3821 |
